# Synthesis of 5,6-Dihydrophenanthridines via Palladium-Catalyzed Intramolecular Dehydrogenative Coupling of Two Aryl C−H Bonds

**DOI:** 10.3390/molecules28062498

**Published:** 2023-03-09

**Authors:** Meng-Yue Wang, Xue-Qing Zhu, Bao-Yin Zhao, Hong-Xia Zhang, Yong-Qiang Wang, Qiong Jia

**Affiliations:** Key Laboratory of Synthetic and Natural Functional Molecule Chemistry of Ministry of Education, School of Foreign Languages, College of Chemistry & Materials Science, Northwest University, Xi’an 710069, China

**Keywords:** 5,6-dihydrophenanthridines, dehydrogenative coupling, palladium catalysis, C−H bond activation

## Abstract

5,6-Dihydrophenanthridines are common aza heterocycle frameworks of natural products and pharmaceuticals. Herein, we reported the first palladium-catalyzed intramolecular C−H/C−H dehydrogenative coupling reaction of two simple arenes to generate 5,6-dihydrophenanthridines. The approach features a broad substrate scope and good tolerance of functional groups, offering an efficient alternative synthesis route for important 5,6-dihydrophenanthridine compounds.

## 1. Introduction

**Figure 1:**
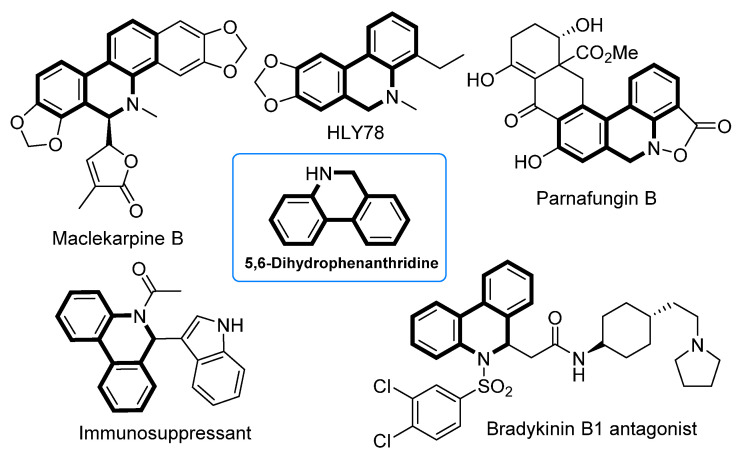
Natural products and drugs containing 5,6-dihydrophenidine skeleton.

**Scheme 1:**
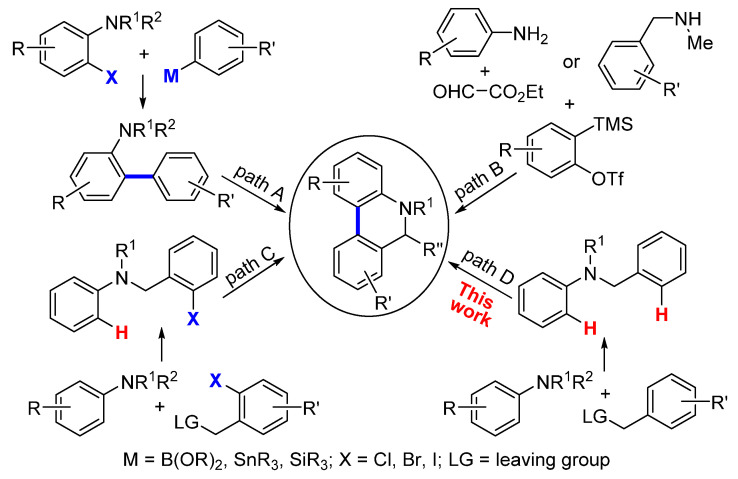
The main strategies to forge aryl−aryl bond of 5,6-dihydrophenanthridines.

5,6-Dihydrophenanthridines are common aza heterocycle frameworks of natural products and pharmaceuticals [1,2,3,4,5,6,7,8,9,10,11,12], exhibiting various biological activities including antibiotic, anti-inflammatory, and anticancer activity (Figure 1) [13,14,15,16,17,18,19]. More recently, studies have shown that the current COVID-19 pandemic is caused by the severe acute respiratory syndrome coronavirus 2 (SARS-CoV-2), and 5,6-dihydrophenanthridine derivatives can interact tightly with SARS-CoV-2 nucleocapsid protein and inhibit the replication of SARS-CoV-2 in vitro [20,21]. Owing to the synthetic challenges in their unique polycyclic skeleton structures, as well as their potential druggability, 5,6-dihydrophenanthridines have aroused considerable interest in synthetic chemists [22,23]. In view of the structural feature of 5,6-dihydrophenanthridines, the formation of their aryl−aryl bond is undoubtedly the key step. To date, there are three main strategies to forge the aryl−aryl bond of 5,6-dihydrophenanthridines (Scheme 1): (1) transition-metal-catalyzed cross-couplings of organometallic aryls with aryl halides (path A) [24,25,26,27,28,29,30]; (2) annulation via benzyne intermediates (path B) [31,32,33]; and (3) the direct arylation of nonactivated aryl C−H bonds with aryl halides (path C) [34,35,36,37,38,39,40,41,42,43,44,45]. Compared to paths A and B, the biggest advantage of path C is that the more expensive and difficult-to-prepare organometallic coupling partner is replaced in this transformation. Nevertheless, the simplest and ideal approach to access 5,6-dihydrophenanthridines is the dehydrogenative coupling of two nonactivated aryl C−H bonds (path D), particularly when aryl halides are not readily available. However, so far there has been no report on using the ideal strategy in the construction of 5,6-dihydrophenanthridine. The major hindrance to the ideal strategy lies in three challenges: (1) the low reactivity of the aryl C−H bond [46]; (2) the regioselectivity issue, especially when there are several reactive sites; and (3) the strong coordinative nitrogen atom of the substrate and product that can easily poison the metal catalyst [23]. In order to accomplish the ideal approach, we hypothesized that an appropriate directing group could be introduced into the substrate to control the regioselectivity whilst at the same time enhancing the reactivity of the aryl C−H bond. Meanwhile, the nitrogen atom of the substrate should be protected by a proper protecting group. In addition, an efficient catalytic system should undoubtedly be sought. Herein, we document the successful execution of this hypothesis to realize the first palladium-catalyzed intramolecular dehydrogenative coupling of two aryl C−H bonds to construct 5,6-dihydrophenanthridines.

## 2. Results and Discussion

### 2.1. Optimization of Reaction Conditions

We commenced this research by investigating the phenol-protecting group, which had the potential directing feature for the *ortho*-functionalization of arenes. A series of phenol-protecting groups were tested with 10 mol% Pd(OAc)_2_ as the catalyst and acetyl as the protecting group of the nitrogen atom in dimethyl sulfoxide (DMSO) under an air atmosphere (Table 1). The reaction did not occur without the phenol-protecting group (entry 1). The protecting groups CONMe_2_ and *O*-(2-pyridyl)carbonyl are excellent directing groups in many C-H functionalization reactions of arenes, but they almost did not work in this reaction (entries 2 and 3). Gratifyingly, when the *O*-(2-pyridyl)sulfonyl group was used as the protecting group, the desired aryl C−H/C−H coupling product **2a** was generated with a 9% yield (entry 4). The structure of **2a** was confirmed by single-crystal X-ray diffraction. We considered that the reactivity of the *O*-(2-pyridyl)-sulfonyl group should lie in it being not only a great directing group but also a good activating group to facilitate the formation of a phenyl−Pd complex through the *ortho* C−H bond activation of phenol [47,48]. Encouraged by the preliminary result, we then carefully examined other parameters of the reaction. The influence of the solvent showed that CF_3_CH_2_OH was the best option, providing **2a** with a 12% yield (entries 5–7). After screening the oxidant, copper(II) trifluoroacetate hydrate provided the highest yield (31%, entries 8–12). Note that without any oxidant the reaction gave **2a** with a 8% yield under an argon atmosphere (entry 13). Next, the reaction temperature was checked (entries 14–17). Increasing the temperature benefitted the reaction, and the yield was increased to 45% at 100 °C. It turned out that the *N*-protecting group was crucial for this reaction. As speculated, the replacement of the *N*-protecting group with Ts, Boc, methyl, or phenyl all led to inferior results (entries 18–21). Then, the palladium source was investigated, indicating that Pd(TFA)_2_ was the best catalyst (entries 22–25). The investigation of the amount of Pd(TFA)_2_ and Cu(TFA)_2_·H_2_O indicated that Pd(TFA)_2_ (15 mol%) and Cu(TFA)_2_·H_2_O (2.2 equiv.) were the best choices (entries 26–31). It is noteworthy that the reaction proceeded similarly under an argon atmosphere (entry 31). Accordingly, the optimized reaction conditions were identified as the following: Pd(TFA)_2_ (15 mol%) and Cu(TFA)_2_·H_2_O (2.2 equiv.) in CF_3_CH_2_OH at 100 °C under an air atmosphere for 20 h (entry 29).

### 2.2. Substrate Scope

**Scheme 2:**
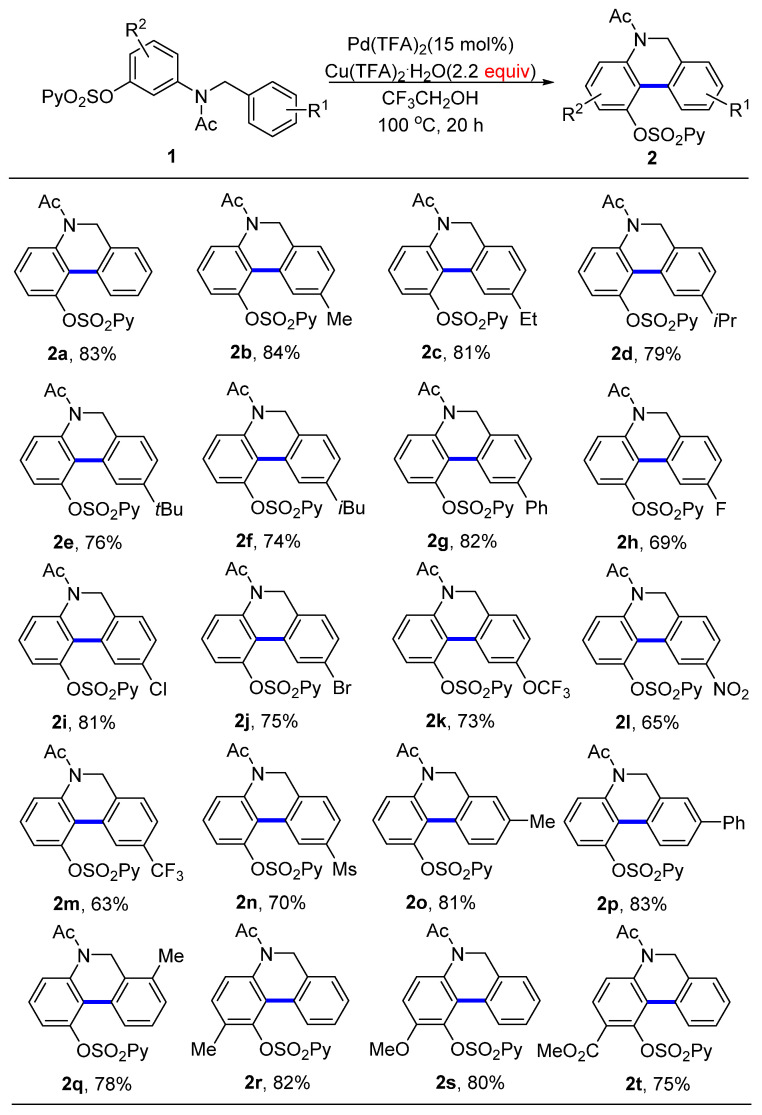
Scope of the two-aryl C−H/C−H coupling reaction. Reaction conditions: **1** (0.5 mmol), Pd(TFA)_2_ (15 mol%) and Cu(TFA)_2_·H_2_O (1.1 mmol) in CF_3_CH_2_OH (2 mL) under air atmosphere at 100 °C for 20 h.

With the optimal conditions in hand, we first examined the effect of the substituents on the right aromatic ring for the aryl C−H/C−H coupling (Scheme 2). A variety of aryls with both electron-donating and electron-withdrawing groups could be engaged in this transformation, providing the desired 5,6-dihydrophenanthridines with good-to-excellent yields. A broad range of functional groups such as alkyls (**2b**−**g**), halides (F, Cl, Br, **2h**−**j**), trifluoromethoxy (**2k**), nitro (**2l**), trifluoromethyl (**2m**), and methyl sulfonyl group (Ms, **3n**) were compatible with this process. These provided synthetically interesting results because such substituents acted as versatile handles for further transformations. The position of the substituent on the aromatic rings had almost no effect on the reactivity (**2o**−**q**). Next, we investigated the substituents on the left phenol ring. Again, the aryl C−H/C−H dehydrogenative coupling reaction was insensitive to the electronic property of the substituent groups such as electron-donating methyl and methoxy, and electron-withdrawing ester groups; all reactions proceeded successfully, affording the desired products with 75–82% yields (**2r**−**t**).

### 2.3. Large-Scale Experiment and Synthetic Application

**Figure 2:**
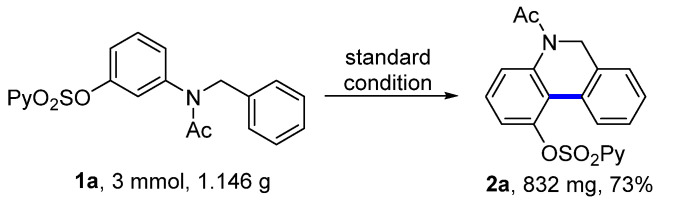
Large-scale experiment.

To test the practicality of this dehydrogenative coupling, a large-scale experiment was carried out. With the above standard reaction conditions, **1a** (1.146 g, 3.0 mmol) provided 5,6-dihydrophenanthridine **2a** (832 mg) with a 73% yield (Figure 2).

**Scheme 3:**
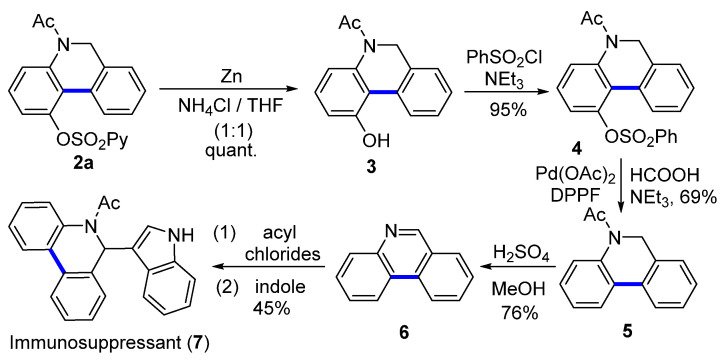
The synthesis of immunosuppressant **7**.

To demonstrate the synthetic application of this methodology, we employed dehydrogenative coupling as the key step to synthesize an inhibitor of potassium channels K_V_1.3 and IK-1 (**7**) (Scheme 3) [6]. First, the 2-pyridysulfonyl group was readily removed by zinc in NH_4_Cl (aq)/THF (1:1) at room temperature, affording **3** in a quantitative yield. Then, the hydroxy group was transformed into a benzenesulfonate group (**4**), which was further removed to give product **5** [49,50]. Next, the acetyl group in product **5** could be readily eliminated using H_2_SO_4_ in MeOH, affording the product phenanthridine **6** with a 76% yield. Finally, the inhibitor dihydrophenanthridine **7** was achieved by the activation of the imine structure in phenanthridine with acyl chlorides to give an intermediary imminium ion followed by in situ nucleophilic attack with indole [6].

### 2.4. Mechanistic Investigations

**Figure 3:**
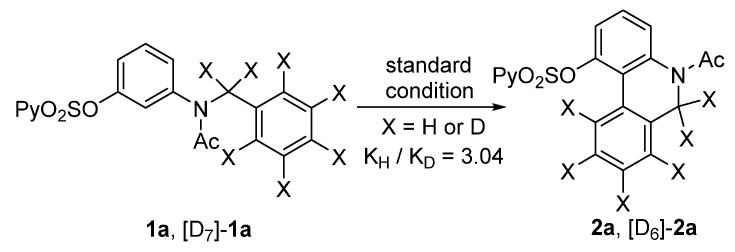
KIE experiment.

To gain insight into the mechanism of this intramolecular dehydrogenative coupling of the two aryl C−H bonds, a kinetic isotope effect (KIE) experiment was performed. The KIE value of the two parallel competition reactions of **1a** and [D_7_]-**1a** was found to be 3.04 (Figure 3). This implied that an electrophilic aromatic palladation mechanism was unlikely, and the cleavage of the C−H bond on the right aromatic ring should be involved in the rate-determining step. In addition, a radical-trapping experiment was performed. In the presence of 1.0 equiv. of 2,2,6,6-tetramethylpiperidine oxide (TEMPO), under the above standard conditions, the reaction of **1a** proceeded well to give **2a** with almost the same yield, illustrating a low possibility for a free radical pathway.

**Scheme 4:**
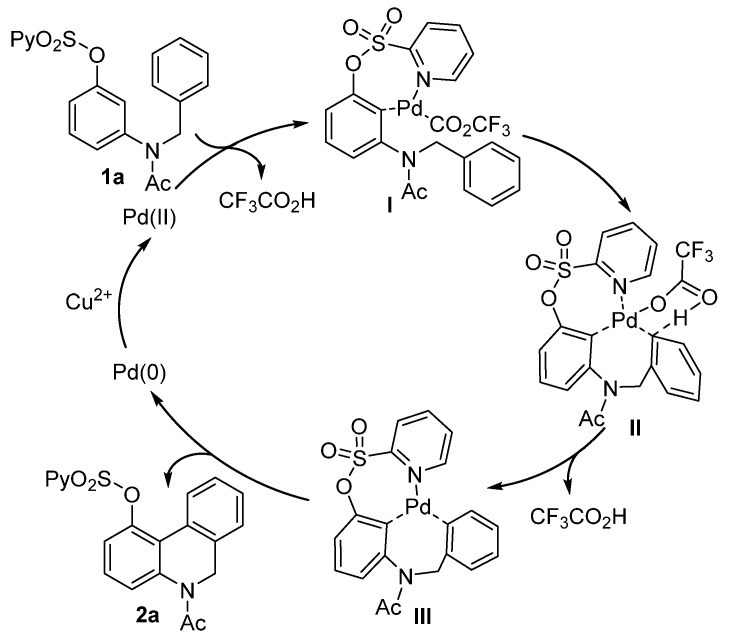
Plausible mechanism.

On the basis of the above results and the relevant literature [51,52,53,54,55], a plausible mechanism is proposed in Scheme 4. Initially, the *O*-(2-pyridyl)sulfonyl group-directed palladation formed complex **I**, which then underwent an intramolecular concerted metalation−deprotonation (CMD) step assisted by trifluoroacetate via a six-membered transition state (**II**) to afford intermediate **III**. Finally, the reductive elimination of intermediate **III** produced the 5,6-dihydrophenanthridine product along with Pd(0), which was reoxidized by Cu^2+^ to regenerate the Pd(II) species to complete the catalytic cycle.

## 3. Materials and Methods

### 3.1. General Information

All reactions were carried out under an air atmosphere. Unless noted otherwise, commercially available chemicals were used without further purification. Flash chromatography was performed with silica gel (200–300 mesh). An oil bath served as the heat source. NMR spectra were acquired on either a Bruker 400 MHz (^1^H at 400 MHz, ^13^C at 100 MHz) or Jeol 400 MHz (^1^H at 400 MHz, ^13^C at 100 MHz) device, and NMR spectra were recorded in CDCl_3_ (TMS, δ = 0.00 ppm for ^1^H and δ = 77.10 ppm for ^13^C), DMSO-d_6_ (δ = 2.50 ppm for ^1^H and δ = 39.52 ppm for ^13^C), or CD_3_OD (δ = 3.31 ppm for ^1^H and δ = 49.00 ppm for ^13^C) using the solvent residue peaks as the internal references. Coupling constants were reported in hertz (Hz). Data for ^1^H NMR spectra were reported as follows: chemical shift (ppm, referenced to protium, s = singlet, d = doublet, t = triplet, q = quartet, dd = doublet of doublets, td = triplet of doublets, ddd = doublet of doublet of doublets, m = multiplet, coupling constant (Hz), and integration). Infrared (IR) data were acquired on a Bruker Invenio-RFT-IR spectrometer. Absorbance frequencies were reported in reciprocal centimeters (cm^−1^). Mass spectra were acquired on a BrukerDaltonics S2 MicroTof-Q II mass spectrometer. X-ray crystal structure analyses were measured on a Bruker Smart APEXIICCD instrument using Mo-K*α* radiation. The structures were solved and refined using the SHELXTL software package.

### 3.2. General Procedure A for Preparation of Substrates

To a solution of *N*-(3-hydroxyphenyl)acetamide (30.0 mmol, 4.51g) in CH_2_Cl_2_ (50 mL), Et_3_N (40.0 mmol, 4.04 g) was added. The mixture was stirred at room temperature for 30 min, and then pyridine-2-sulfonyl chloride (30.0 mmol, 5.31 g) was added. The mixture was stirred overnight. The solvent was removed by distillation, and then EtOAc (50 mL) was added. The resulting solution was washed with water (3 × 10 mL) and brine (3 × 5 mL), dried over MgSO_4_, and concentrated. The residue was used without further purification in the next step.

To a solution of residue (1 mmol) in anhydrous tetrahydrofuran (10 mL), NaH (60 mg, 1.5 mmol, 60%) was added. The mixture was stirred at room temperature for 30 min, and then benzyl bromide (1.2 mmol) was added. The mixture was stirring at room temperature until completion of the reaction (monitored by TLC). Then, the reaction mixture was filtered and purified by column chromatography (eluent: PET (petroleum ether):EA (ethyl acetate) = 2:1).

### 3.3. General Procedure B for Preparation of Substrates

To a solution of 3-aminophenol (30.0 mmol, 4.51 g) in CH_2_Cl_2_ (50 mL), NEt_3_ (40.0 mmol, 4.04 g) was added. The mixture was stirred at room temperature for 30 min, and then pyridine-2-sulfonyl chloride (30.0 mmol, 5.31 g) was added. The mixture was stirred overnight. The solvent was removed by distillation, and then EtOAc (50 mL) was added. The resulting solution was washed with water (3 × 10 mL) and brine (3 × 5 mL), dried over MgSO_4_, and concentrated. The residue was used without further purification in the next step.

To a solution of residue (1 mmol) in CH_2_Cl_2_ (10 mL), acetic anhydride (122 mg, 1.2 mmol) was added. The mixture was stirred overnight. The solvent was removed by distillation, and then CH_2_Cl_2_ (15 mL) was added. The resulting solution was washed with water (3 × 5 mL) and brine (3 × 5 mL), dried over MgSO_4_, and concentrated. The residue was purified by column chromatography (eluent: PET:EA = 2:1) to give the product.

To a solution of above product (1 mmol) in anhydrous tetrahydrofuran (10 mL), NaH (60 mg, 1.5 mmol, 60%) was added. The mixture was stirred at room temperature for 30 min, and then benzyl bromide (1.2 mmol) was added. The mixture was stirring at room temperature until completion of the reaction (monitored by TLC). Then, the reaction mixture was filtered and purified by column chromatography (eluent: PET:EA = 2:1).

### 3.4. General Procedure C for Preparation of Products

3-(*N*-benzylacetamido)phenyl pyridine-2-sulfonate derivatives **1** (0.2 mmol), Pd(TFA)_2_ (15 mol %), and Cu(TFA)_2_·H_2_O (2.2 equiv.) were added to a 10 mL round-bottom flask in CF_3_CH_2_OH, and these were held at 100 °C under an air atmosphere until completion of the reaction (monitored by TLC). Then, purification by flash chromatography afforded product **2**.

### 3.5. The Synthesis of Immunosuppressant 7

*1-Hydroxyphenanthridin-5(6H)-yl)ethan-1-one* (**3**)*:* A suspension of 5-acetyl-5,6-dihydrophenanthridin-1-yl pyridine-2-sulfonate **2** (152mg, 0.4 mmol) and Zn powder (not activated, 1.3 g, 20 mmol) in a 1:1 mixture of THF and saturated NH_4_Cl solution in water (20 mL) was stirred at 30 °C until consumption of the starting material (monitored by TLC). The mixture was filtered over a pad of celite to remove the Zn. The filtrate was extracted with EA (15 mL) and washed with a saturated aqueous solution of ammonium chloride and brine. The combined organic phase was dried over MgSO_4_ and concentrated. The residue was purified by flash chromatography to afford the corresponding product **3** (eluent: PET:EA = 1:1, Rf = 0.2) as a white solid. Quant.: m. p. = 212–213 °C; ^1^H NMR (400 MHz, CD_3_OD) δ 8.46 (d, *J* = 7.9 Hz, 1H), 7.33–7.29 (m, 1H), 7.28–7.19 (m, 2H), 7.16–7.12 (m, 1H), 6.86 (d, *J* = 7.4 Hz, 2H), 4.61 (d, *J* = 9.8 Hz, 2H), 2.09 (s, 3H); ^13^C NMR (100 MHz, CD_3_OD) δ 170.4, 155.3, 139.7, 135.2, 130.5, 128.1, 127.7, 126.9, 126.6, 125.0, 117.0, 115.8, 114.2, 45.3, 20.9; HRMS (ESI) m/z calculated for C_15_H_13_NO_2_Na [M+Na]^+^: 262.0838; found 262.0830.

*5-Acetyl-5,6-dihydrophenanthridin-1-yl benzenesulfonate* (**4**): The compound **3** (0.4 mmol, 96 mg) was dissolved in DCM (10 mL) in a bottle filled under an argon atmosphere. The solution was cooled to 0 °C. Then, NEt_3_ (0.078 mL, 0.56 mmol, 1.4 equiv) was added dropwise to the solution, which was followed by the addition of benzenesulfonyl chloride (84.5 mg, 0.48 mmol, 1.2 equiv). After 5 min, the ice bath was removed, and the reaction was monitored by TLC. Once the phenol was completely consumed, the reaction was stopped. The solvent was evaporated under a vacuum, and the residue was purified by flash column chromatography to give **4** as a colorless gel (144mg, 95%). ^1^H NMR (400 MHz, CDCl_3_) δ 8.01 (dd, *J* = 7.9, 1.3 Hz, 1H), 7.32–7.41 (m, 5H), 7.29–7.25 (m, 1H), 7.24–7.19 (m, 2H), 7.14–7.07 (m, 3H), 4.27 (s, 2H), 2.14 (s, 3H); ^13^C NMR (100 MHz, CDCl_3_) δ 168.9, 146.39, 140.2, 136.1, 133.9, 129.3, 128.4, 128.3 (3C), 128.2 (2C), 127.8, 127.7, 125.7, 123.5, 122.0, 45.0, 21.7.

*1-(Phenanthridin-5(6H)-yl)ethan-1-one* (**5**) [56]: To a bottle were added **4** (144 mg, 0.38 mmol), Pd(OAc)_2_ (8.84 mg 0.039 mmol), DPPF (7.3 mg, 0.039 mmol), Et_3_N (119 mg 1.18 mmol), HCOOH (36 mg 0.78 mmol), and DMF (4 mL). The atmosphere was replaced by an argon atmosphere. The mixture was stirred at 80 °C for 6 h. After the usual workup, the residue was purified by flash column chromatography to give **5** as a yellow gel (58.5 mg, 69%). H NMR (400 MHz, CDCl_3_) δ 7.75 (dd, *J* = 8.6, 6.1 Hz, 2H), 7.45–7.14 (m, 6H), 4.90 (s, 2H), 2.14 (s, 3H); ^13^C NMR (100 MHz, CDCl_3_) δ 169.3, 137.8, 135.0, 131.6, 129.6, 127.9 (2C), 127.6, 126.2, 126.0, 124.5, 124.3, 123.2, 44.8, 22.0.

*Phenanthridin* (**6**) [57]: A stock solution was prepared by dropwise addition of concentrated sulfuric acid (1 mL) to reagent-grade methanol (5 mL) in a scintillation vial (20 mL in volume). The stock solution was stirred for 5 min, and then the solution was cooled to room temperature. A bottle equipped with a spin bar was charged with 1-(phenanthridin-5(6H)-yl)ethan-1-one **5** (0.26 mmol). A fraction of the stock solution (0.5 mL) was added slowly to the solid reagent, and the reaction mixture was stirred at 60 °C for 15 min. Then, the reaction mixture was cooled to room temperature, and distilled water (2 mL), EtOAc (5 mL), and saturated Na_2_CO_3_ solution (2 mL) were added dropwise to the reaction mixture. The aqueous phase was extracted with EA (2 × 8 mL), and the combined organic fractions were dried over anhydrous Na_2_SO_4_ or MgSO_4_. The combined organic fractions were filtered through celite, and the filtrate was concentrated. The residue was purified by flash column chromatography to give phenanthridine **6** as a white solid (35.7 mg, 76%). m. p. = 106–109 °C; ^1^H NMR (400 MHz, CDCl_3_) δ 9.30 (s, 1H), 8.61 (dd, *J* = 12.6, 7.4 Hz, 2H), 8.20 (d, *J* = 9.5 Hz, 1H), 8.06 (d, *J* = 7.9 Hz, 1H), 7.90–7.85 (m, 1H), 7.79–7.67 (m, 3H); ^13^C NMR (100 MHz, CDCl_3_) δ 153.7, 144.5, 132.6, 131.1, 130.2, 128.9, 128.8, 127.6, 127.2, 126.5, 124.1, 122.3, 122.0.

*Immunosuppressant* (**7**) [58]: Phenanthridine (0.2 mmol) was dissolved in dry tetrahydrofuran (2.0 mL) under an argon atmosphere. After cooling to 0°C, the appropriate acetyl chloride (1.2 mmol) was added dropwise. The mixture was stirred for 2 h at room temperature and then cooled to 0 °C again. Triethylamine (0.3 mmol) was added followed by 1*H*-indole (1.5 mmol). After stirring for 3 h at room temperature, water was added, and the mixture was extracted several times with ethyl acetate. After washing the combined organic phases with brine and drying over Na_2_SO_4_, the solvent was removed in vacuo. The residue was purified by flash column chromatography to give immunosuppressant **7** as a white solid (35.7 mg, 76%). m. p. = 211–213 °C; ^1^H NMR (400 MHz, DMSO-*d*6) δ 10.68 (s, 1H), 7.99 (dd, *J* = 26.9, 7.7 Hz, 2H), 7.73 (d, *J* = 7.7 Hz, 1H), 7.52–7.47 (m, 2H), 7.41–7.37 (m, 1H), 7.33 (s, 1H), 7.26 (d, *J* = 7.8 Hz, 2H), 7.17 (d, *J* = 4.4 Hz, 2H), 7.05–7.01 (m, 2H), 6.11 (s, 1H), 2.19 (s, 3H); ^13^C NMR (100 MHz, DMSO-*d*_6_) δ 168.6, 137.4, 136.3, 135.7, 130.8, 128.8, 128.2, 128.0, 127.8, 126.9, 126.00, 125.96, 125.8, 124.6, 124.2, 123.8, 121.4, 119.0 (2C), 113.9, 111.6, 49.7, 22.6; IR (KBr): 3016, 2961, 2927, 1662, 1455, 1426, 1455, 1426, 1378.9, 1213, 1186, 1118, 1049, 1049, 987, 874, 753, 667, 594 cm^−1^; HRMS (ESI) *m*/*z* calculated for C_23_H_18_N_2_ONa [M+Na]^+^: 361.1311; found 361.1307.

### 3.6. Characterization Data of Substrates and Products

3-(*N*-Benzylacetamido)phenyl pyridine-2-sulfonate (**1a**): The product was synthesized by general method A (eluent: EA:PE = 1:2, R_f_ = 0.23); 278.9 mg, 73% yield; white solid; m. p. = 78–79 °C; ^1^H NMR (400 MHz, CDCl_3_) δ 8.73 (s, 1H), 7.92–7.88 (m, 2H), 7.58 (d, *J* = 3.0 Hz, 1H), 7.38–7.18 (m, 4H), 7.15–7.07 (m, 3H), 6.99–6.71 (m, 2H), 4.80 (s, 2H), 1.81 (s, 3H); ^13^C NMR (100 MHz, CDCl_3_) δ 169.9, 153.2, 150.6, 149.9, 143.9, 138.3, 136.9, 130.5, 128.5 (2C), 128.3, 127.5, 127.0, 124.2, 122.4, 121.9, 52.7, 22.7; IR (KBr): 3063, 2924, 1658, 1601, 1581, 1485, 1428, 1376, 1196, 1117, 938, 616, 593 cm^−1^; HRMS (ESI) m/z calculated for C_20_H_18_N_2_O_4_SNa [M+Na]^+^: 405.0879; found 405.0871.

3-(*N*-(4-Methylbenzyl)acetamido)phenyl pyridine-2-sulfonate (**1b**): The product was synthesized by general method A (eluent: EA:PE = 1:2, R_f_ = 0.22); 332.7 mg, 84% yield; white solid; m. p. = 83–94 °C; ^1^H NMR (400 MHz, CDCl_3_) δ 8.73 (s, 1H), 7.92–7.88 (m, 2H), 7.58 (d, *J* = 3.3 Hz, 1H), 7.34–7.21 (m, 1H), 7.11 (d, *J* = 7.0 Hz, 1H), 7.01 (dd, *J* = 16.4, 7.4 Hz, 4H), 6.88 (d, *J* = 7.3 Hz, 1H), 6.83 (s, 1H), 4.75 (s, 2H), 2.28 (s, 3H), 1.78 (s, 3H); ^13^C NMR (100 MHz, CDCl_3_) δ 169.8, 153.2, 150.6, 149.9, 143.9, 138.3, 137.1, 133.9, 130.5, 129.2, 128.5, 128.3, 127.1, 124.2, 122.5, 121.9, 52.4, 22.8, 21.1; IR (KBr): 2924, 1731, 1620, 1572, 1490, 1467, 1378, 1195, 1067, 1032, 840 cm^−1^; HRMS (ESI) m/z calculated for C_21_H_20_N_2_O_4_SNa [M+Na]^+^: 419.1036; found 419.1033.

3-(*N*-(4-Ethylbenzyl)acetamido)phenyl pyridine-2-sulfonate (**1c**): The product was synthesized by general method A (eluent: EA:PE = 1:1, R_f_ = 0.25); 369.9 mg, 91% yield; colorless gel; ^1^H NMR (400 MHz, CDCl_3_) δ 8.69 (d, *J* = 4.6 Hz, 1H), 7.85 (d, *J* = 2.1 Hz, 2H), 7.54 (ddd, *J* = 6.7, 4.7, 2.4 Hz, 1H), 7.25–7.19 (m, 1H), 7.12–6.94 (m, 5H), 6.86 (d, *J* = 8.1 Hz, 1H), 6.80 (s, 1H), 4.72 (s, 2H), 2.54 (q, *J* = 7.6 Hz, 2H), 1.74 (s, 3H), 1.13 (t, *J* = 7.6 Hz, 3H); ^13^C NMR (100 MHz, CDCl_3_) δ 169.7, 152.9, 150.4, 149.7, 143.8, 143.2, 138.2, 133.9, 130.3, 128.3, 128.1, 127.8, 126.9, 124.0, 122.3, 121.7, 52.2, 28.3, 22.5, 15.3; IR (KBr): 3057, 2926, 1914, 1854, 1806, 1529, 1428, 1379, 1197, 1139, 1118, 926, 867, 772, 594, 553 cm^−1^; HRMS (ESI) m/z calculated for C_22_H_23_N_2_O_4_S [M+H]^+^: 411.1373; found 411.1384.

3-(*N*-(4-Isopropylbenzyl)acetamido)phenyl pyridine-2-sulfonate (**1d**): The product was synthesized by general method A (eluent: EA:PE = 1:1, R_f_ = 0.25); 361.3 mg, 85% yield; colorless gel; ^1^H NMR (400 MHz, CDCl_3_) δ 8.75 (d, *J* = 4.7 Hz, 1H), 7.93 (d, *J* = 7.9 Hz, 2H), 7.61 (ddd, *J* = 6.8, 4.6, 2.2 Hz, 1H), 7.32–7.26 (m, 1H), 7.19–7.03 (m, 5H), 6.94 (d, *J* = 8.0 Hz, 1H), 6.88 (s, 1H), 4.79 (s, 2H), 2.87 (p, *J* = 7.0 Hz, 1H), 1.81 (s, 3H), 1.21 (d, *J* = 2.0 Hz, 6H); ^13^C NMR (100 MHz, CDCl_3_) δ 13C NMR (101 MHz, CDCl_3_) δ 169.6, 152.8, 150.4, 149.7, 147.8, 143.8, 138.2, 134.0, 130.3, 128.2, 128.1, 126.9, 126.3, 124.0, 122.2, 121.6, 52.2, 33.5, 23.8, 22.5; IR (KBr): 3551, 2959, 1643, 1601, 1485, 1428, 1380, 1197, 1138, 1117, 1085, 1017, 926, 865, 768, 751, 592, 548 cm^−1^; HRMS (ESI) m/z calculated for C_23_H_25_N_2_O_4_S [M+H]^+^: 425.1530; found 425.1546.

3-(*N*-(4-(*tert*-Butyl)benzyl)acetamido)phenyl pyridine-2-sulfonate (**1e**): The product was synthesized by general method A (eluent: EA:PE = 1:2, R_f_ = 0.28); 363.7 mg, 83% yield; white solid; m. p. = 82–93 °C; ^1^H NMR (400 MHz, CDCl_3_) δ 8.76 (d, *J* = 4.5 Hz, 1H), 7.93 (d, *J* = 4.5 Hz, 2H), 7.60 (dd, *J* = 8.8, 4.6 Hz, 1H), 7.29 (dd, *J* = 11.3, 5.4 Hz, 3H), 7.15 (d, *J* = 7.8 Hz, 1H), 7.07 (d, *J* = 8.2 Hz, 2H), 6.94 (d, *J* = 7.5 Hz, 1H), 6.89 (s, 1H), 4.78 (s, 2H), 1.81 (s, 3H), 1.29 (s, 9H); ^13^C NMR (100 MHz, CDCl_3_) δ 169.9, 153.3, 150.6, 150.4, 149.9, 144.1, 138.2, 133.8, 130.4, 128.2 (2C), 127.1, 125.4, 124.2, 122.5, 121.8, 52.4, 34.5, 31.3, 22.7; IR (KBr): 2960, 1660, 1601, 1513, 1428, 1377, 1295, 1196, 1117, 926, 865, 801, 722, 550 cm^−1^; HRMS (ESI) m/z calculated for C_24_H_26_N_2_O_4_SNa [M+Na]^+^: 461.1505; found 461.1515.

3-(*N*-(4-Isobutylbenzyl)acetamido)phenyl pyridine-2-sulfonate (**1f**): The product was synthesized by general method A (eluent: EA:PE = 1:1, R_f_ = 0.25); 403.9 mg, 92% yield; colorless gel; ^1^H NMR (400 MHz, CDCl_3_) δ 8.76 (d, *J* = 4.6 Hz, 1H), 7.93 (d, *J* = 3.6 Hz, 2H), 7.63–7.57 (m, 1H), 7.29–7.25 (m, 1H), 7.13 (d, *J* = 7.7 Hz, 1H), 7.06–7.00 (m, 4H), 6.93–6.84 (m, 2H), 4.79 (s, 2H), 2.43 (d, *J* = 7.2 Hz, 2H), 1.81 (s, 3H), 0.87 (d, *J* = 6.6 Hz, 6H); ^13^C NMR (100 MHz, CDCl_3_) δ 169.7, 153.1, 150.5, 149.8, 143.8, 140.8, 138.2, 134.0, 130.3, 129.1, 128.2, 128.1, 127.0, 124.1, 122.4, 121.7, 52.3, 44.9, 30.0, 22.6, 22.2; IR (KBr): 2955, 2925, 1659, 1601, 1581, 1485, 1428, 1379, 1197, 1138, 1117, 926, 866, 771, 736, 594, 550 cm^−1^; HRMS (ESI) m/z calculated for C_24_H_27_N_2_O_4_S [M+H]^+^: 439.1686; found 439.1683.

3-(*N*-([1,1’-Biphenyl]-4-ylmethyl)acetamido)phenyl pyridine-2-sulfonate (**1g**): The product was synthesized by general method A (eluent: EA:PE = 1:2, R_f_ = 0.24); 348.2 mg, 76% yield; white solid; m. p. = 102–103 °C; ^1^H NMR (400 MHz, CDCl_3_) δ 8.69 (d, *J* = 4.6 Hz, 1H), 7.89 (d, *J* = 7.8 Hz, 1H), 7.85–7.81 (m, 1H), 7.63–7.54 (m, 2H), 7.50 (dd, *J* = 8.2, 4.6 Hz, 3H), 7.43–7.39 (m, 2H), 7.33–7.26 (m, 2 H), 7.21 (d, *J* = 8.1 Hz, 2H), 7.14 (d, *J* = 7.9 Hz, 1H), 6.96 (d, *J* = 7.5 Hz, 1H), 6.91 (s, 1H), 4.86 (s, 2H), 1.84 (s, 3H); ^13^C NMR (100 MHz, CDCl_3_) δ 169.8, 153.0, 150.4, 149.8, 143.8, 140.3, 140.0, 138.1, 135.8, 130.4, 128.8, 128.6, 128.1, 127.2, 127.0, 126.9, 126.8, 123.9, 122.3, 121.8, 52.2, 22.6; IR (KBr): 3058, 2926, 1661, 1601, 1581, 1486, 1428, 1379, 1294, 1197, 801, 766, 616, 594 cm^−1^; HRMS (ESI) m/z calculated for C_26_H_22_N_2_O_4_SNa [M+Na]^+^: 481.1192; found 481.1190.

3-(*N*-(4-Fluorobenzyl)acetamido)phenyl pyridine-2-sulfonate (**1h**): The product was synthesized by general method A (eluent: EA:PE = 1:2, R_f_ = 0.27); 336.1 mg, 84% yield; white solid; m. p. = 83–84 °C; ^1^H NMR (400 MHz, CDCl_3_) δ 8.81–8.74 (m, 1H), 8.04–7.90 (m, 2H), 7.62 (ddd, *J* = 6.8, 4.7, 2.1 Hz, 1H), 7.33–7.28 (m, 1H), 7.19 (d, *J* = 8.2 Hz, 1H), 7.16–7.10 (m, 2H), 6.93 (dd, *J* = 17.8, 9.0 Hz, 3H), 6.86 (s, 1H), 4.80 (s, 2H), 1.83 (s, 3H); ^13^C NMR (100 MHz, CDCl_3_) δ 169.9, 162.2 (*J*_C-F_ = 244.0 Hz), 153.6, 150.7, 150.1, 143.8, 138.3, 132.8 (*J*_C-F_ = 3.0 Hz), 130.62, 130.58, 130.5, 128.2, 127.1, 124.1, 122.4 (*J*_C-F_ = 45.0 Hz), 115.4 (*J*_C-F_ = 22.0 Hz), 51.9, 22.7; IR (KBr): 2922, 1660, 1602, 1581, 1509, 1428, 1378, 1303, 1197, 1139, 1086, 945, 773 cm^−1^; HRMS (ESI) m/z calculated for C_20_H_17_FN_2_O_4_SNa [M+Na]^+^: 423.0785; found 423.0795.

3-(*N*-(4-Chlorobenzyl)acetamido)phenyl pyridine-2-sulfonate (**1i**): The product was synthesized by general method A (eluent: EA: PE = 1:2, R_f_ = 0.23); 332.8 mg, 80% yield; white solid; m. p. = 84–85 °C; ^1^H NMR (400 MHz, CDCl_3_) δ 8.73 (d, *J* = 4.3 Hz, 1H), 7.93 (d, *J* = 3.7 Hz, 2H), 7.60 (dd, *J* = 8.9, 4.3 Hz, 1H), 7.32–7.28 (m, 1H), 7.20 (d, *J* = 8.0 Hz, 2H), 7.15 (d, *J* = 8.1 Hz, 1H), 7.07 (d, *J* = 8.1 Hz, 2H), 6.91 (d, *J* = 7.7 Hz, 1H), 6.85 (s, 1H), 4.77 (s, 2H), 1.81 (s, 3H); ^13^C NMR (100 MHz, CDCl_3_) δ 169.9, 153.3, 150.6, 150.0, 143.6, 138.3, 135.5, 133.2, 130.6, 130.0, 128.6, 128.2, 126.9, 124.1, 122.4, 122.0, 51.9, 22.6; IR (KBr): 2923, 1661, 1601, 1486, 1428, 1379, 1291, 1197, 1139, 1117, 925, 802, 773 cm^−1^; HRMS (ESI) m/z calculated for C_20_H_17_ClN_2_O_4_SNa [M+Na]^+^: 439.0490; found 439.0492.

3-(*N*-(4-Bromobenzyl)acetamido)phenyl pyridine-2-sulfonate (**1j**): The product was synthesized by general method A (eluent: EA:PE = 1:1, R_f_ = 0.23); 291.1 mg, 76% yield; yellow gel; ^1^H NMR (400 MHz, CDCl_3_) δ 8.75 (d, *J* = 4.7 Hz, 1H), 7.95 (d, *J* = 6.5 Hz, 2H), 7.75–7.54 (m, 1H), 7.52–7.26 (m, 3H), 7.16 (d, *J* = 8.6 Hz, 1H), 7.03 (d, *J* = 8.1 Hz, 2H), 6.94 (d, *J* = 8.0 Hz, 1H), 6.87 (s, 1H), 4.78 (s, 2H), 1.83 (s, 3H); ^13^C NMR (100 MHz, CDCl_3_) δ 169.9, 153.1, 150.5, 149.9, 143.5, 138.2, 135.9, 131.4, 130.6, 130.2, 128.2, 126.8, 124.0, 122.2, 121.9, 121.3, 51.9, 22.5; IR (KBr): 2959, 2920, 1660, 1602, 1582, 1486, 1428, 1379, 1197, 1139, 1118, 926, 866, 801, 773, 594, 551 cm^−1^; HRMS (ESI) m/z calculated for C_20_H_17_BrN_2_O_4_SNa [M+Na]^+^: 482.9985; found 482.9979.

3-(*N*-(4-(Trifluoromethoxy)benzyl)acetamido)phenyl pyridine-2-sulfonate (**1k**): The product was synthesized by general method A (eluent: EA:PE = 1:2, R_f_ = 0.24); 358.8 mg, 77% yield; white solid; m. p. = 77–78 °C; ^1^H NMR (400 MHz, CDCl_3_) δ 8.75 (d, *J* = 4.4 Hz, 1H), 8.03–7.84 (m, 2H), 7.67–7.58 (m, 1H), 7.35–7.30 (m, 1H), 7.19 (d, *J* = 8.5 Hz, 3H), 7.11 (d, *J* = 8.1 Hz, 2H), 6.92 (d, *J* = 8.9 Hz, 2H), 4.82 (s, 2H), 1.84 (s, 3H); ^13^C NMR (100 MHz, CDCl_3_) δ 170.1, 153.4, 150.6, 150.1, 148.5, 143.8, 138.3, 135.7, 130.7, 130.1, 128.2, 126.9, 124.1, 122.5, 122.1, 120.9, 120.4 (*J*_C-F_ = 255.0 Hz), 52.0, 22.7; IR (KBr): 2924, 1662, 1602, 1582, 1429, 1380, 1255, 1227, 1196, 991, 867, 801 cm^−1^; HRMS (ESI) m/z calculated for C_21_H_17_F_3_N_2_O_5_SNa [M+Na]^+^: 489.0702; found 489.0700.

3-(*N*-(4-Nitrobenzyl)acetamido)phenyl pyridine-2-sulfonate (**1l**): The product was synthesized by general method A (eluent: EA:PE = 1:2, R_f_ = 0.20); 354.5 mg, 83% yield; white solid; m. p. = 100–101 °C; ^1^H NMR (400 MHz, CDCl_3_) δ 8.75 (d, *J* = 4.6 Hz, 1H), 8.11 (d, *J* = 8.6 Hz, 2H), 8.01–7.93 (m, 2H), 7.66–7.57 (m, 1H), 7.36–7.32 (m, 3H), 7.16 (d, *J* = 7.8 Hz, 1H), 6.96 (d, *J* = 6.5 Hz, 2H), 4.92 (s, 2H), 1.87 (s, 3H); ^13^C NMR (100 MHz, CDCl_3_) δ 170.2, 153.4, 150.6, 150.2, 147.3, 144.4, 143.5, 138.4, 130.8, 129.3, 128.3, 126.7, 124.1, 123.8, 122.4, 122.2, 52.2, 22.6; IR (KBr): 3077, 2923, 1662, 1601, 1518, 1428, 1378, 1344, 1197, 1140, 931, 861 cm^−1^; HRMS (ESI) *m*/*z* calculated for C_20_H_17_N_3_O_6_SNa [M+Na]^+^: 450.0730; found 450.0731.

3-(*N*-(4-(Trifluoromethyl)benzyl)acetamido)phenyl pyridine-2-sulfonate (**1m**): The product was synthesized by general method A (eluent: EA:PE = 1:2, R_f_ = 0.26); 342.1 mg, 76% yield; white solid; m. p. = 85–86 °C; ^1^H NMR (400 MHz, CDCl_3_) δ 8.78–8.65 (m, 1H), 7.99–7.87 (m, 2H), 7.71–7.56 (m, 1H), 7.52 (d, *J* = 8.1 Hz, 2H), 7.36–7.25 (m, 3H), 7.16 (d, *J* = 7.7 Hz, 1H), 6.94 (d, *J* = 8.2 Hz, 2H), 4.88 (s, 2H), 1.85 (s, 3H); ^13^C NMR (100 MHz, CDCl_3_) δ 170.1, 153.4, 150.6, 150.1, 143.7, 141.0, 138.3, 130.7, 129.7 (*J*_C-F_ = 32.0 Hz), 128.8, 128.2, 126.8, 125.5, 124.08, 124.07 (*J*_C-F_ = 270.0 Hz), 122.4, 122.1, 52.4, 22.6; IR (KBr): 2925, 1663, 1602, 1486, 1380, 1324, 1198, 1115, 1066, 930, 802, 593 cm^−1^; HRMS (ESI) m/z calculated for C_21_H_17_F_3_N_2_O_4_SNa [M+Na]^+^: 473.0753; found 473.0750.

3-(*N*-(4-(Methylsulfonyl)benzyl)acetamido)phenyl pyridine-2-sulfonate (**1n**): The product was synthesized by general method A (eluent: EA:PE = 1:1, R_f_ = 0.22); 358.8 mg, 78% yield; yellow solid; m. p. = 132–137 °C; ^1^H NMR (400 MHz, CDCl_3_) δ 8.75 (d, *J* = 4.6 Hz, 1H), 8.00–7.95 (m, 2H), 7.86 (d, *J* = 8.2 Hz, 2H), 7.67–7.59 (m, 1H), 7.44–7.35 (m, 3H), 7.18 (d, *J* = 8.9 Hz, 1H), 6.98 (d, *J* = 7.4 Hz, 1H), 6.91 (s, 1H), 4.93 (s, 2H), 3.06 (s, 3H), 1.89 (s, 3H); ^13^C NMR (100 MHz, CDCl_3_) δ 167.0, 152.7, 150.4, 149.7, 143.2, 143.0, 139.3, 138.3, 130.6, 129.0, 128.3, 127.3, 126.5, 123.9, 122.1, 121.9, 52.0, 44.2, 22.3; IR (KBr): 2954, 2923, 1659, 1600, 1548, 1378, 1302, 1197, 1147, 1089, 958, 931, 764, 594, 553, 523 cm^−1^; HRMS (ESI) m/z calculated for C_21_H_20_N_2_O_6_S_2_Na [M+Na]^+^: 483.0655; found 483.0652.

3-(*N*-(3-Methylbenzyl)acetamido)phenyl pyridine-2-sulfonate (**1o**): The product was synthesized by general method A (eluent: EA:PE = 1:2, R_f_ = 0.22); 281.2 mg, 71% yield; white solid; m. p. = 68–69 °C; ^1^H NMR (400 MHz, CDCl_3_) δ 8.78–8.74 (m, 1H), 7.95–7.88 (m, 2H), 7.64–7.56 (m, 1H), 7.29 (dd, *J* = 9.8, 6.4 Hz, 1H), 7.16–7.12 (m, 2H), 7.05 (d, *J* = 7.5 Hz, 1H), 6.98 (s, 1H), 6.90 (dd, *J* = 14.3, 6.7 Hz, 3H), 4.79 (s, 2H), 2.30 (s, 3H), 1.83 (s, 3H); ^13^C NMR (100 MHz, CDCl_3_) δ 169.9, 153.4, 150.6, 149.9, 144.0, 138.2 (2C), 136.8, 130. 4, 129.3, 128.3 (2C), 128.2, 127.1, 125.6, 124.2, 122.5, 121.9, 52.7, 22.7, 21.4; IR (KBr): 2923, 1660, 1602, 1515, 1486, 1428, 1294, 1197, 1139, 1117, 1039, 948, 801, 773cm^−1^; HRMS (ESI) m/z calculated for C_21_H_20_N_2_O_4_SNa [M+Na]^+^: 419.1036; found 419.1026.

3-(*N*-([1,1’-Biphenyl]-3-ylmethyl)acetamido)phenyl pyridine-2-sulfonate (**1p**): The product was synthesized by general method A (eluent: EA:PE = 1:1, R_f_ = 0.24); 394.7 mg, 86% yield; yellow gel; ^1^H NMR (400 MHz, CDCl_3_) δ 8.64 (d, *J* = 4.1 Hz, 1H), 7.84–7.72 (m, 2H), 7.48 (dd, *J* = 19.3, 7.3 Hz, 4H), 7.42–7.22 (m, 6H), 7.01–6.86 (m, 2H), 7.15–7.07(m, 2H), 4.89 (s, 2H), 1.83 (s, 3H); ^13^C NMR (100 MHz, CDCl_3_) δ 170.0, 153.2, 150.6, 150.0, 143.8, 141.3, 140.7, 138.2, 137.4, 130.6, 129.0, 128.8, 128.2, 127.5, 127.4, 127.3, 127.1(2C), 126.4, 124.2, 122.5, 122.1, 52.7, 22.8; IR (KBr): 2920, 2851, 1660, 1601, 1582, 1484, 1428, 1378, 1261, 1197, 1139, 1117, 936, 761, 737, 699, 593, 550 cm^−1^; HRMS (ESI) m/z calculated for C_26_H_23_N_2_O_4_S [M+H]^+^: 459.1373; found 459.1357.

3-(*N*-(2-Methylbenzyl)acetamido)phenyl pyridine-2-sulfonate (**1q**): The product was synthesized by general method A (eluent: EA:PE = 1:2, R_f_ = 0.24); 308.9 mg, 78% yield; white solid; m. p. = 104–105 °C; ^1^H NMR (400 MHz, CDCl_3_) δ 8.75 (d, *J* = 4.5 Hz, 1H), 7.94–7.87 (m, 2H), 7.59 (dd, *J* = 8.5, 4.9 Hz, 1H), 7.27–7.23 (m, 1H), 7.18–6.98 (m, 5H), 6.90–6.86 (m, 2H), 4.87 (s, 2H), 2.13 (s, 3H), 1.84 (s, 3H); ^13^C NMR (100 MHz, CDCl_3_) δ 169.7, 153.3, 150.6, 149.9, 143.7, 138.2, 136.3, 134.5, 130.3 (2C), 129.2, 128.2, 127.5, 127.0, 125.9, 124.2, 122.4, 121.9, 49.9, 22.7, 19.0; IR (KBr): 2924, 1662, 1602, 1486, 1379, 1302, 1198, 1140, 1117, 925, 803, 770, 738, 617 cm^−1^; HRMS (ESI) m/z calculated for C_21_H_20_N_2_O_4_SNa [M+Na]^+^: 419.1036; found 419.1046.

5-(*N*-Benzylacetamido)-2-methylphenyl pyridine-2-sulfonate (**1r**): The product was synthesized by general method B (eluent: EA:PE = 1:1, R_f_ = 0.22); 333.5 mg, 84% yield; yellow gel; ^1^H NMR (400 MHz, CDCl_3_) δ 8.76 (d, *J* = 4.6 Hz, 1H), 7.93 (d, *J* = 3.4 Hz, 2H), 7.64–7.58 (m, 1H), 7.26 (d, *J* = 6.7 Hz, 3H), 7.19–7.11 (m, 3H), 6.90 (s, 1H), 6.80 (d, *J* = 8.1 Hz, 1H), 4.82 (s, 2H), 2.22 (s, 3H), 1.87 (s, 3H); ^13^C NMR (100 MHz, CDCl_3_) δ 170.0, 153.4, 150.5, 148.2, 141.1, 138.2, 136.9, 132.0, 131.3, 128.4, 128.3, 128.1, 127.2, 126.6, 123.8, 122.0, 52.4, 22.5, 16.0; IR (KBr): 2955, 2924, 2853, 1659, 15613, 1579, 1504, 1428, 1197, 1104, 1087, 941, 867, 801, 751, 715, 596, 552cm^−1^; HRMS (ESI) m/z calculated for C_21_H_21_N_2_O_4_S [M+H]^+^: 397.1217; found 397.1207.

5-(*N*-Benzylacetamido)-2-methoxyphenyl pyridine-2-sulfonate (**1s**): The product was synthesized by general method B (eluent: EA:PE = 1:1, R_f_ = 0.22); 370.8 mg, 90% yield; yellow gel; ^1^H NMR (400 MHz, CDCl_3_) δ 8.75 (d, *J* = 5.1 Hz, 1H), 8.03–7.86 (m, 2H), 7.62 (dd, *J* = 4.4, 1.9 Hz, 1H), 7.36–7.11 (m, 5H), 6.96–6.92 (m, 1H), 6.86–6.77 (m, 2H), 4.82 (s, 2H), 3.59 (s, 3H), 1.88 (s, 3H); ^13^C NMR (100 MHz, CDCl_3_) δ 170.4, 154.0, 151.2, 150.2, 138.3, 138.0, 137.0, 135.0, 128.6, 128.3, 127.9, 127.8, 127.3, 124.1, 123.7, 112.7, 55.8, 52.6, 22.6; IR (KBr): 2925, 2850, 1654, 1509, 1380, 1295, 1271, 1197, 1112, 780, 764, 599, 545 cm^−1^; HRMS (ESI) m/z calculated for C_21_H_20_N_2_O_5_SNa [M+Na]^+^: 435.0985; found 435.0984.

Methyl 4-(*N*-benzylacetamido)-2-((pyridin-2-ylsulfonyl)oxy)benzoate (**1t**): The product was synthesized by general method B (eluent: EA:PE = 1:1, R_f_ = 0.22); 295.5 mg, 67% yield; colorless gel; ^1^H NMR (400 MHz, CDCl_3_) δ 8.68 (d, *J* = 4.7 Hz, 1H), 8.03–7.93 (m, 2H), 7.88 (d, *J* = 8.3 Hz, 1H), 7.63–7.57 (m, 1H), 7.26 (d, *J* = 6.8 Hz, 3H), 7.18–7.08 (m, 3H), 7.04 (d, *J* = 8.3 Hz, 1H), 4.89 (s, 2H), 3.80 (s, 3H), 1.96 (s, 3H); ^13^C NMR (100 MHz, CDCl_3_) δ 169.8, 164.3, 154.0, 150.4, 148.8, 147.1, 138.4, 136.6, 132.8, 128.6, 128.3, 128.1, 127.6, 126.4, 124.3, 123.9, 123.8, 52.7, 52.5, 22.9; IR (KBr): 2954, 2924, 2853, 1726, 1665, 1462, 1378, 1264, 1199, 1153, 740, 593 cm^−1^; HRMS (ESI) m/z calculated for C_22_H_21_N_2_O_6_S [M+H]^+^: 441.1115; found 441.1104.

5-Acetyl-5,6-dihydrophenanthridin-1-yl pyridine-2-sulfonate (**2a**): The product was synthesized by general method C (eluent: EA:PE = 1:1, R_f_ = 0.22); 63.9 mg, 83% yield; white solid; m. p. = 151–152 °C; ^1^H NMR (400 MHz, CDCl_3_) δ 8.37 (d, *J* = 3.1 Hz, 1H), 8.00 (d, *J* = 7.4 Hz, 1H), 7.70 (d, *J* = 7.7 Hz, 1H), 7.66–7.62 (m, 1H), 7.11–7.52 (m, 7H), 4.43 (s, 2H), 2.12 (s, 3H); ^13^C NMR (100 MHz, CDCl_3_) δ 168.8, 153.0, 149.6, 146.8, 140.3, 137.5, 135.9, 131.9, 128.2, 128.1, 127.9, 127.8, 127.8 (2C), 125.7, 123.8, 123.7, 121.9, 45.1, 22.1; IR (KBr): 2924, 2853, 1663, 1444, 1377, 1218, 1187, 1048, 960, 765, 735, 553 cm^−1^; HRMS (ESI) m/z calculated for C_20_H_16_N_2_O_4_SNa [M+Na]^+^: 403.0723; found 403.0720.

5-Acetyl-9-methyl-5,6-dihydrophenanthridin-1-yl pyridine-2-sulfonate (**2b**): The product was synthesized by general method C (eluent: EA:PE = 1:1, R_f_ = 0.24); 67.8 mg, 84% yield; white solid; m. p. = 145–146 °C; ^1^H NMR (400 MHz, CDCl_3_) δ 8.39 (d, *J* = 2.4 Hz, 1H), 7.83–7.72 (m, 2H), 7.69–7.65 (m, 1H), 7.46–7.19 (m, 4H), 6.99 (dd, *J* = 20.2, 7.5 Hz, 2H), 4.40 (s, 2H), 2.28 (s, 3H), 2.11 (s, 3H); ^13^C NMR (100 MHz, CDCl_3_) δ 168.8, 153.3, 149.6, 146.7, 140.4, 137.5, 137.1, 133.1, 128.7, 128.6, 127.8, 127.7, 125.5, 123.8, 123.7, 123.5, 121.9, 120.4, 44.8, 22.1, 21.4; IR (KBr): 2824, 1664, 1608, 1454, 1427, 1378, 1340, 1207, 1190, 1086, 1058, 767 cm^−1^; HRMS (ESI) m/z calculated for C_21_H_18_N_2_O_4_SNa [M+Na]^+^: 417.0879; found 417.0887.

5-Acetyl-9-ethyl-5,6-dihydrophenanthridin-1-yl pyridine-2-sulfonate (**2c**): The product was synthesized by general method C (eluent: EA:PE = 1:1, R_f_ = 0.20); 66.3 mg, 81% yield; white solid; m. p. = 123–125 °C; ^1^H NMR (400 MHz, CDCl_3_) 8.31 (s, 1H), 7.77 (s, 1H), 7.65 (d, *J* = 7.8 Hz, 1H), 7.60–7.53 (m, 1H), 7.36–7.12 (m, 4H), 6.99–6.90 (m, 2H), 4.33 (s, 2H), 2.52 (q, *J* = 7.6 Hz, 2H), 2.04 (s, 3H), 1.17 (t, *J* = 7.6 Hz, 3H).; ^13^C NMR (100 MHz, CDCl_3_) δ 168.8, 153.1, 149.6, 146.8, 143.5, 140.3, 137.4, 133.3, 128.0, 127.8, 127.7, 127.6 (2C), 125.6, 124.0, 123.6 (2C), 121.8, 44.8, 28.8, 22.1, 15.6; IR (KBr): 3055, 2958, 2854, 1662, 1454, 1426, 1375, 1188, 1118, 1044, 948, 902, 878, 826, 810, 765, 724, 593, 564 cm^−1^; HRMS (ESI) m/z calculated for C_22_H_21_N_2_O_4_S [M+H]^+^: 409.1217; found 409.1201

5-Acetyl-9-isopropyl-5,6-dihydrophenanthridin-1-yl pyridine-2-sulfonate (**2d**): The product was synthesized by general method C (eluent: EA:PE = 1:1, R_f_ = 0.3); 72.6mg, 79% yield; yellow solid; m. p. = 163–165 °C; ^1^H NMR (400 MHz, CDCl_3_) δ 8.39 (s, 1H), 7.91 (s, 1H), 7.71 (d, *J* = 7.8 Hz, 1H), 7.67–7.59 (m, 1H), 7.46–7.13 (m, 4H), 7.09–7.01 (m, 2H), 4.39 (s, 2H), 2.89 (p, *J* = 7.1, 5.8 Hz, 1H), 2.12 (s, 3H), 1.27 (d, *J* = 7.0 Hz, 6H); ^13^C NMR (100 MHz, CDCl_3_) δ 168.8, 152.9, 149.5, 148.1, 146.7, 140.3, 137.4, 133.4, 127.8, 127.7 (2C), 126.4, 126.3, 125.5, 124.1, 123.7, 123.6, 121.6, 44.8, 33.9, 23.9, 22.1; IR (KBr): 3016, 2961, 2927, 1662, 1455, 1426, 1379, 1213, 1186, 1118, 1049, 987, 949, 753, 667, 594 cm^−1^; HRMS (ESI) m/z calculated for C_22_H_22_N_2_O_4_SNa [M+Na]^+^: 445.1192; found 445.1188.

5-Acetyl-9-(*tert*-butyl)-5,6-dihydrophenanthridin-1-yl pyridine-2-sulfonate (**2e**): The product was synthesized by general method C (eluent: EA:PE = 1:1, R_f_ = 0.21); 73.3 mg, 84% yield; white solid; m. p. = 181–182 °C; ^1^H NMR (400 MHz, CDCl_3_) δ 8.37 (s, 1H), 8.11 (s, 1H), 7.68 (d, *J* = 7.2 Hz, 1H), 7.66–7.56 (m, 1H), 7.48–7.20 (m, 5H), 7.06 (d, *J* = 7.9 Hz, 1H), 4.37 (s, 2H), 2.12 (s, 3H), 1.36 (s, 9H); ^13^C NMR (100 MHz, CDCl_3_) δ 168.8, 152.9, 150.6, 150.5, 149.5, 146.8, 140.3, 138.2, 137.4, 133.1, 127.7 (2C), 125.4, 125.3, 124.4 123.8, 123.6, 121.5, 44.8, 34.8, 31.3, 22.2; IR (KBr): 2960, 1664, 1455, 1427, 1377, 1340, 1207, 1187, 1085, 1055, 882, 766 cm^−1^; HRMS (ESI) m/z calculated for C_24_H_24_N_2_O_4_SNa [M+Na]^+^: 459.1349; found 459.1345.

5-Acetyl-9-isobutyl-5,6-dihydrophenanthridin-1-yl pyridine-2-sulfonate (**2f**): The product was synthesized by general method C (eluent: EA:PE = 1:1, R_f_ = 0.22); 64.5 mg, 74% yield; yellow solid; m. p. = 148–152 °C; ^1^H NMR (400 MHz, CDCl_3_) δ 8.42 (s, 1H), 7.80 (s, 1H), 7.73 (d, *J* = 7.8 Hz, 1H), 7.69–7.62 (m, 1H), 7.40–7.29 (m, 3H), 7.22 (s, 1H), 7.04 (d, *J* = 7.6 Hz, 1H), 6.96 (d, *J* = 7.7 Hz, 1H), 4.49 (s, 2H), 2.42 (d, *J* = 7.1 Hz, 2H), 2.13 (s, 3H), 2.01–1.59 (m, 1H), 0.91 (d, *J* = 6.7 Hz, 6H); ^13^C NMR (100 MHz, CDCl_3_) δ 168.9, 153.4, 149.7, 146.8, 141.2, 140.4, 137.5, 133.3, 129.0, 128.8, 127.9, 127.7 (2C), 125.4, 124.1, 123.7 (2C), 121.7, 45.3, 44.9, 30.1, 22.4, 22.2; IR (KBr): 2955, 2925, 2868, 1663, 1579, 1455, 1418, 1377, 1340, 1206, 1190, 1118, 1052, 987, 950, 909, 871, 812, 768, 727, 593, 555 cm^−1^; HRMS (ESI) m/z calculated for C_24_H_24_N_2_O_4_SNa [M+Na]^+^: 459.1349; found 459.1345.

5-Acetyl-9-phenyl-5,6-dihydrophenanthridin-1-yl pyridine-2-sulfonate (**2g**): The product was synthesized by general method C (eluent: EA:PE = 1:1, R_f_ = 0.20); 76.5 mg, 82% yield; white solid; m. p. = 141–142 °C; ^1^H NMR (400 MHz, CDCl_3_) δ 8.30 (d, *J* = 3.3 Hz, 1H), 8.24 (d, *J* = 1.2 Hz, 1H), 7.69 (d, *J* = 7.8 Hz, 1H), 7.63 (d, *J* = 7.3 Hz, 2H), 7.55–7.49 (m, 1H), 7.51–7.36 (m, 6H), 7.31–7.19 (m, 3H), 4.52 (s, 2H), 2.16 (s, 3H); ^13^C NMR (100 MHz, CDCl_3_) δ 168.9, 153. 1, 149.7, 146.7, 140.5, 140.3, 137.5, 134.9, 128.9 (2C), 128.5, 128.2, 127.9, 127.7, 127.5, 127.1, 126.9, 126.7, 126.2, 123.8, 123.5, 121.9, 44.8, 22.2; IR (KBr): 3057, 2924, 1664, 1605, 1452, 1376, 1340, 1217, 1205, 1186, 765, 742 cm^−1^; HRMS (ESI) m/z calculated for C_26_H_20_N_2_O_4_SNa [M+Na]^+^: 479.1036; found 479.1030.

5-Acetyl-9-fluoro-5,6-dihydrophenanthridin-1-yl pyridine-2-sulfonate (**2h**): The product was synthesized by general method C (eluent: EA:PE = 1:1, R_f_ = 0.26); 53.7 mg, 69% yield; white solid; m. p. = 151–152 °C; ^1^H NMR (400 MHz, CDCl_3_) δ 8.46 (d, *J* = 3.4 Hz, 1H), 7.87 (d, *J* = 7.8 Hz, 1H), 7.80–7.76 (m, 1H), 7.75–7.64 (m, 1H), 7.52–7.37 (m, 3H), 7.30 (s, 1H), 7.13 (dd, *J* = 8.0, 5.9 Hz, 1H), 6.90–6.86 (m, 1H), 4.58 (s, 2H), 2.14 (s, 3H); ^13^C NMR (100 MHz, CDCl_3_) δ 168.9, 161.6 (*J*_C-F_ = 243.0 Hz), 153.2, 149.8, 146.7, 140.2, 137.9, 131.7, 129.7 (*J*_C-F_ = 5.0 Hz), 128.7, 128.0, 126.9 (*J*_C-F_ = 4.0 Hz), 123.7 (2C), 122.8, 122.0, 115.0 (*J*_C-F_ = 16.2 Hz), 114.8 (*J*_C-F_ = 13.3 Hz), 44.4, 22.0; IR (KBr): 3728, 2361, 2341, 1665, 1606, 1591, 1454, 1420, 1381, 1340, 1219, 1184, 814 cm^−1^; HRMS (ESI) m/z calculated for C_20_H_15_FN_2_O_4_SNa [M+Na]^+^: 421.0629; found 421.0620.

5-Acetyl-9-chloro-5,6-dihydrophenanthridin-1-yl pyridine-2-sulfonate (**2i**): The product was synthesized by general method C (eluent: EA:PE = 1:1, R_f_ = 0.22); 63.8 mg, 81% yield; white solid; m. p. = 172–173 °C; ^1^H NMR (400 MHz, CDCl_3_) δ 8.44 (d, *J* = 3.9 Hz, 1H), 7.92 (d, *J* = 7.7 Hz, 2H), 7.81–7.77 (m, 1H), 7.39 (ddd, *J* = 12.4, 11.3, 6.3 Hz, 3H), 7.27 (d, *J* = 7.3 Hz, 1H), 7.18–7.06 (m, 2H), 4.60 (s, 2H), 2.13 (s, 3H); ^13^C NMR (100 MHz, CDCl_3_) δ 168.8, 153.5, 149.9, 146.7, 140.4, 137.9, 134.4, 133.3, 129.8, 128.8, 128.0, 127.9 (2C), 126.9, 123.8, 123.7, 122.5, 122.2, 44.5, 22.1; IR (KBr): 2923, 2852, 2361, 1665, 1606, 1467, 1452, 1403, 1380, 1338, 1218, 1186, 816,764 cm^−1^; HRMS (ESI) m/z calculated for C_20_H_15_ClN_2_O_4_SNa [M+Na]^+^: 437.0333; 437.0330.

5-Acetyl-9-bromo-5,6-dihydrophenanthridin-1-yl pyridine-2-sulfonate (**2j**): The product was synthesized by general method C (eluent: EA:PE = 1:1, R_f_ = 0.22); 68.5mg, 75% yield; white solid; m. p. = 167–169 °C; ^1^H NMR (400 MHz, CDCl_3_) δ 8.40 (d, *J* = 5.2 Hz, 1H), 8.01 (d, *J* = 3.3 Hz, 1H), 7.91 (d, *J* = 8.0 Hz, 1H), 7.82–7.74 (m, 1H), 7.46–7.30 (m, 3H), 7.29–7.18 (m, 2H), 7.01 (d, *J* = 8.1 Hz, 1H), 4.42 (s, 2H), 2.10 (s, 3H); ^13^C NMR (100 MHz, CDCl_3_) δ 168.9, 153.7, 149.9, 146.8, 140.4, 138.1, 134.9, 130.9, 130.8, 130.3 128.8, 128.0, 127.2 (2C), 123.8 (2C), 122.3 (2C), 121.4, 44.7, 22.2; IR (KBr): 3055, 2924, 2853, 1661, 1578, 1451, 1373, 1337, 1217, 1185, 1117, 871, 842, 763, 735, 721, 592, 556 cm^−1^; HRMS (ESI) m/z calculated for C_20_H_15_N_2_O_4_SBrNa [M+Na]^+^: 480.9828; found 480.9827.

5-Acetyl-9-(trifluoromethoxy)-5,6-dihydrophenanthridin-1-yl pyridine- 2-sulfonate (**2k**): The product was synthesized by general method C (eluent: EA:PE = 1:1, R_f_ = 0.21); 77.0 mg, 83% yield; white solid; m. p. = 145–146 °C; ^1^H NMR (400 MHz, CDCl_3_) δ 8.44 (d, *J* = 4.1 Hz, 1H), 7.87 (d, *J* = 7.7 Hz, 2H), 7.78–7.74 (m, 1H), 7.50–7.37 (m, 3H), 7.30 (s, 1H), 7.21 (d, *J* = 8.3 Hz, 1H), 7.06 (d, *J* = 8.2 Hz, 1H), 4.59 (s, 2H), 2.16 (s, 3H); ^13^C NMR (100 MHz, CDCl_3_) δ 168.8, 153.2, 149.7, 148.2, 146.8, 140.2, 137.9, 134.5, 134.4, 129.9, 128.9, 127.9, 126.9, 123.7, 122.5, 121.8, 120.8, 120.4, 120.3 (*J*_C-F_ = 255.0 Hz), 44.5, 22.1; IR (KBr): 2923, 2360, 1666, 1455, 1427, 1382, 1340, 1253, 1215, 1118, 872, 811, 765 cm^−1^; HRMS (ESI) m/z calculated for C_21_H_15_F_3_N_2_O_5_SNa [M+Na]^+^: 487.0546; found 487.0556.

5-Acetyl-9-nitro-5,6-dihydrophenanthridin-1-yl pyridine-2-sulfonate (**2l**): The product was synthesized by general method C (eluent: EA:PE = 1:1, R_f_ = 0.23); 60.9 mg, 68% yield; white solid; m. p. = 167–168 °C; ^1^H NMR (400 MHz, CDCl_3_) δ 8.81 (d, *J* = 1.8 Hz, 1H), 8.47 (d, *J* = 3.9 Hz, 1H), 8.09–8.03 (m, 1H), 7.99 (d, *J* = 7.8 Hz, 1H), 7.89–7.77 (m, 1H), 7.51–7.41 (m, 4H), 7.37–7.26 (m, 1H), 4.82 (s, 2H), 2.17 (s, 3H); ^13^C NMR (100 MHz, CDCl_3_) δ 168.9, 153.6, 150.1, 147.4, 146.8, 142.7, 140.4, 138.2, 129.8, 129.5, 128.3, 126.7, 123.9, 123.8, 123.0, 122.8, 122.1, 121.9, 44.9, 22.1; IR (KBr): 2922, 2852, 2361, 1667, 1608, 1524, 1383, 1347, 1219, 1187, 872, 835, 811 cm^−1^; HRMS (ESI) m/z calculated for C_20_H_15_N_3_O_6_SNa [M+Na]^+^: 448.0574; found 448.0581.

5-Acetyl-9-(trifluoromethyl)-5,6-dihydrophenanthridin-1-yl pyridine-2-sulfonate (**2m**): The product was synthesized by general method C (eluent: EA:PE = 1:1, R_f_ = 0.22); 65.4 mg, 73% yield; white solid; m. p. = 153–154 °C; ^1^H NMR (400 MHz, CDCl_3_) δ 8.41 (d, *J* = 4.1 Hz, 1H), 8.24 (s, 1H), 7.89 (d, *J* = 7.9 Hz, 1H), 7.78–7.74 (m, 1H), 7.45 (dd, *J* = 16.0, 8.3 Hz, 3H), 7.40–7.35 (m, 1H), 7.34–7.27 (m, 2H), 4.70 (s, 2H), 2.16 (s, 3H); ^13^C NMR (100 MHz, CDCl_3_) δ 168.9, 153.4, 149.8, 146.9, 140.4, 139.6, 138.0, 129.8 (*J*_C-F_ = 32.4 Hz), 129.1, 129.0, 128.0, 126.3 (*J*_C-F_ = 1.8 Hz), 124.8 (*J*_C-F_ = 4.2 Hz, 2C), 123.8 (2C), 123.7 (*J*_C-F_ = 270.8 Hz), 122.4, 122.0, 44.9, 22.1; IR (KBr): 2924, 2361, 1667, 1455, 1422, 1382, 1331, 1254, 1167, 1119, 1078, 810, 764 cm^−1^; HRMS (ESI) m/z calculated for C_21_H_15_F_3_N_2_O_4_SNa [M+Na]^+^: 471.0597; found 471.0591.

5-Acetyl-9-(methylsulfonyl)-5,6-dihydrophenanthridin-1-yl pyridine-2-sulfonate (**2n**): The product was synthesized by general method C (eluent: EA:PE = 1.5:1, R_f_ = 0.20); 64.1 mg, 70% yield; yellow solid; m. p. = 132–137 °C; ^1^H NMR (400 MHz, CDCl_3_) δ 8.69 (s, 1H), 8.49 (d, *J* = 4.7 Hz, 1H), 7.98 (d, *J* = 7.9 Hz, 1H), 7.94–7.76 (m, 2H), 7.51–7.37 (m, 4H), 7.29 (s, 1H), 4.77 (s, 2H), 3.17 (s, 3H), 2.17 (s, 3H); ^13^C NMR (100 MHz, CDCl_3_) δ 169.0, 153.0, 149.9, 146.9, 141.4, 140.4, 140.1, 138.4, 129.7, 129.3, 128.2, 127.2, 126.7 (2C), 124.4, 123.8, 122.5, 121.8, 44.9, 44.3, 22.1; IR (KBr): 3019, 2925, 1664, 1377, 1317, 1214, 1148, 1118, 745, 667, 562 cm^−1^; HRMS (ESI) m/z calculated for C_21_H_18_N_2_O_6_S_2_Na [M+Na]^+^: 481.0498; found 481.0496.

5-Acetyl-8-methyl-5,6-dihydrophenanthridin-1-yl pyridine-2-sulfonate (**2o**): The product was synthesized by general method C (eluent: EA:PE = 1:1, R_f_ = 0.23); 62.3 mg, 79% yield; white solid; m. p. = 190–191 °C; ^1^H NMR (400 MHz, CDCl_3_) δ 8.41 (s, 1H), 7.90 (d, *J* = 8.0 Hz, 1H), 7.73 (d, *J* = 7.8 Hz, 1H), 7.68–7.64 (m, 1H), 7.39–7.30 (m, 3H), 7.22 (s, 1H), 7.03 (d, *J* = 7.6 Hz, 1H), 6.96 (s, 1H), 4.48 (s, 2H), 2.32 (s, 3H), 2.12 (s, 3H); ^13^C NMR (100 MHz, CDCl_3_) δ 169.0, 153.2, 149.7, 146.7, 140.2, 138.4, 137.5, 135.9, 128.5, 128.1, 127.7, 127.5, 126.4, 125.3, 123.9 (2C), 123.7, 121.9, 45.2, 22.2, 21.2; IR (KBr): 2923, 2853, 1664, 1456, 1378, 1212, 1188, 1118, 1054, 986, 959, 866 cm^−1^; HRMS (ESI) m/z calculated for C_21_H_18_N_2_O_4_SNa [M+Na]^+^: 417.0879; found 417.0883.

5-Acetyl-8-phenyl-5,6-dihydrophenanthridin-1-yl pyridine-2-sulfonate (**2p**): The product was synthesized by general method C (eluent: EA:PE = 1:1, R_f_ = 0.22); 75.9mg, 83% yield; white solid; m. p. = 209–210 °C; ^1^H NMR (400 MHz, CDCl_3_) δ 8.36 (d, *J* = 4.7 Hz, 1H), 8.05 (d, *J* = 8.2 Hz, 1H), 7.74 (d, *J* = 7.9 Hz, 1H), 7.60 (dd, *J* = 19.8, 7.8 Hz, 3H), 7.42 (ddd, *J* = 30.2, 13.9, 7.7 Hz, 7H), 7.30–7.21 (m, 2H), 4.48 (s, 2H), 2.15 (s, 3H); ^13^C NMR (100 MHz, CDCl_3_) δ 169.1, 153.1, 149.7, 146.9, 140.8, 140.3, 139.8, 137.5, 136.5, 129.0, 128.7, 128.0, 127.9, 127.8, 127.0, 126.9, 126.3, 124.1, 124.0, 123.7 (2C), 122.2, 45.2, 22.2; IR (KBr): 3010, 2924, 2853, 1660, 1466, 1377, 1336, 1214, 1188, 1118, 986, 957, 866, 809, 748, 699, 594, 557, 541cm^−1^; HRMS (ESI) m/z calculated for C_26_H_21_N_2_O_4_S [M+H]^+^: 457.1217; found 457.1198.

5-Acetyl-7-methyl-5,6-dihydrophenanthridin-1-yl pyridine-2-sulfonate (**2q**): The product was synthesized by general method C (eluent: EA:PE = 1:1, R_f_ = 0.25); 63.1 mg, 80% yield; white solid; m. p. = 159–160 °C; ^1^H NMR (400 MHz, CDCl_3_) δ 8.39 (s, 1H), 7.84 (d, *J* = 7.7 Hz, 1H), 7.74–7.56 (m, 2H), 7.48–7.21 (m, 4H), 7.13–7.09 (m, 1H), 7.03 (d, *J* = 7.3 Hz, 1H), 4.43 (s, 2H), 2.34 (s, 3H), 2.13 (s, 3H); ^13^C NMR (100 MHz, CDCl_3_) δ 168.9, 153.1, 149.5, 146.7, 140.3, 137.4, 135.2, 133.5, 129.9, 127.9, 127.4, 127.1, 125.9 (2C), 124.4, 123.9, 123.5, 122.0, 41.7, 22.1, 19.1; IR (KBr): 2924, 1664, 1480, 1454, 1427, 1377, 1339, 1189, 1118, 813, 766, 621 cm^−1^; HRMS (ESI) m/z calculated for C_21_H_18_N_2_O_4_SNa [M+Na]^+^: 417.0879; found 417.0870.

5-Acetyl-2-methyl-5,6-dihydrophenanthridin-1-yl pyridine-2-sulfonate (**2r**): The product was synthesized by general method C (eluent: EA:PE = 1:1, R_f_ = 0.22); 71.7 mg, 91% yield; white solid; m. p. = 206–208 °C; ^1^H NMR (400 MHz, CDCl_3_) δ 8.47 (s, 1H), 7.92 (d, *J* = 7.9 Hz, 1H), 7.80 (d, *J* = 7.8 Hz, 1H), 7.76–7.69 (m, 1H), 7.47–7.32 (m, 1H), 7.22–7.07 (m, 3H), 7.04–6.97 (m, 1H), 6.95 (d, *J* = 8.8 Hz, 1H), 4.54 (s, 2H), 3.76 (s, 3H), 2.09 (s, 3H); ^13^C NMR (100 MHz, CDCl_3_) δ 169.0, 153.8, 151.1, 149.6, 145.6, 138.1, 137.4, 135.7, 131.8, 130.3, 128.9, 127.9, 127.7, 127.4, 125.6, 124.1, 123.5, 123.0, 45.3, 22.1, 17.7; IR (KBr): 3019, 2361, 1654, 1481, 1452, 1377, 1214, 1117, 1049, 868, 743, 667, 591, 546 cm^−1^; HRMS (ESI) m/z calculated for C_21_H_19_N_2_O_4_S [M+H]^+^: 395.1060; found 395.1061.

5-Acetyl-2-methoxy-5,6-dihydrophenanthridin-1-yl pyridine-2-sulfonate (**2s**): The product was synthesized by general method C (eluent: EA:PE = 1:1, R_f_ = 0.22); 65.9 mg, 80% yield; yellow solid; m. p. = 210–213 °C; ^1^H NMR (400 MHz, CDCl_3_) δ 8.36 (d, *J* = 4.7 Hz, 1H), 7.75 (d, *J* = 7.9 Hz, 1H), 7.59 (dd, *J* = 16.3, 7.7 Hz, 2H), 7.27 (d, *J* = 7.7 Hz,2H), 7.15 (s, 1H), 7.10–7.00 (m, 2H), 6.94 (d, *J* = 7.8 Hz, 1H), 4.62 (s, 2H), 2.55 (s, 3H), 2.10 (s, 3H); ^13^C NMR (100 MHz, CDCl_3_) δ 169.0, 153.8, 149.6, 145.7, 138.2, 137.4, 135.8, 131.8, 130.4, 128.9, 127.9, 127.7 (2C), 127.4, 125.7, 124.1, 123.5, 123.0, 45.3, 22.2, 17.8; IR (KBr): 3008, 2926, 2848, 1652, 1481, 1429, 1376, 1236, 1196, 1111, 1049, 1019, 986, 964, 867, 790, 746, 703, 666, 587, 553 cm^−1^; HRMS (ESI) m/z calculated for C_21_H_20_N_2_O_5_SNa [M+Na]^+^: 435.0985; found 435.0984.

Methyl 5-acetyl-1-((pyridin-2-ylsulfonyl)oxy)-5,6-dihydrophenanthridine-2-carb- oxylate (**2t**): The product was synthesized by general method C (eluent: EA:PE = 1:1, R_f_ = 0.22); 65.7 mg, 75% yield; white solid; m. p. = 200–203 °C; ^1^H NMR (400 MHz, CDCl_3_) δ 8.20 (dd, *J* = 4.7, 1.9 Hz, 1H), 7.97 (dd, *J* = 14.3, 8.9 Hz, 2H), 7.68 (d, *J* = 7.8 Hz, 1H), 7.61–7.57 (m, 1H), 7.29 (s, 1H), 7.20 (ddd, *J* = 7.6, 4.7, 1.2 Hz, 1H), 7.12 (dd, *J* = 5.8, 3.3 Hz, 2H), 7.08–7.01 (m, 1H), 4.53 (s, 2H), 4.01 (s, 3H), 2.17 (s, 3H); ^13^C NMR (100 MHz, CDCl_3_) δ 168.7, 165.5, 153.3, 149.6, 145.6, 143.3, 137.5, 135.5, 130.7, 128.5, 128.4, 128.1, 127.9, 127.7, 125.5, 124.9 (2C), 123.6, 123.1, 52.7, 45.0, 22.4; IR (KBr): 2925, 2851, 1656, 1496, 1481, 1452, 1429, 1376, 1237, 1197, 1164, 1112, 1050, 1019, 988, 965, 867, 808, 790, 725, 702, 615, 587, 554 cm^−1^; HRMS (ESI) m/z calculated for C_22_H_18_N_2_O_6_SNa [M+Na]^+^: 461.0778; found 461.0772.

## 4. Conclusions

In summary, we developed the first palladium-catalyzed intramolecular C−H/C−H dehydrogenative coupling between two simple arenes to construct 5,6-dihydrophenanthridines. The approach featured a broad substrate scope and good tolerance of functional groups, and it offers an efficient alternative synthesis route for the important 5,6-dihydrophenanthridine compounds. Further application and mechanistic studies are currently ongoing in our laboratory.

## Figures and Tables

**Table 1:** Optimization of conditions for the synthesis of 5,6-dihydrophenanthridine *^a^*.

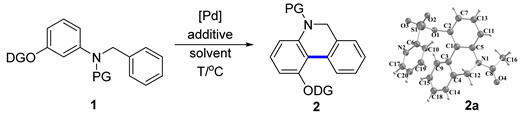							
Entry	DG	PG	Pd cat.	Oxidant	T /°C	Solvent	Yield (%) *^b^*
1	-H	Ac	Pd(OAc)_2_	air	70	DMSO	0
2	-CONMe_2_	Ac	Pd(OAc)_2_	air	70	DMSO	trace
3	-CO(2-Py)	Ac	Pd(OAc)_2_	air	70	DMSO	trace
4	-SO_2_(2-Py)	Ac	Pd(OAc)_2_	air	70	DMSO	9
5	-SO_2_(2-Py)	Ac	Pd(OAc)_2_	air	70	HFIP	11
6	-SO_2_(2-Py)	Ac	Pd(OAc)_2_	air	70	CF_3_CH_2_OH	12
7 *^c^*	-SO_2_(2-Py)	Ac	Pd(OAc)_2_	air	70	Other solvents	<6
8	-SO_2_(2-Py)	Ac	Pd(OAc)_2_	Cu(TFA)_2_·H_2_O	70	CF_3_CH_2_OH	31
9	-SO_2_(2-Py)	Ac	Pd(OAc)_2_	Cu(OAc)_2_·H_2_O	70	CF_3_CH_2_OH	22
10	-SO_2_(2-Py)	Ac	Pd(OAc)_2_	Cu(OAc)_2_	70	CF_3_CH_2_OH	21
11	-SO_2_(2-Py)	Ac	Pd(OAc)_2_	CuF_2_·2H_2_O	70	CF_3_CH_2_OH	29
12 *^c^*	-SO_2_(2-Py)	Ac	Pd(OAc)_2_	Other oxidants	70	CF_3_CH_2_OH	<20
13	-SO_2_(2-Py)	Ac	Pd(OAc)_2_	Argon	70	CF_3_CH_2_OH	8
14	-SO_2_(2-Py)	Ac	Pd(OAc)_2_	Cu(TFA)_2_·H_2_O	60	CF_3_CH_2_OH	23
15	-SO_2_(2-Py)	Ac	Pd(OAc)_2_	Cu(TFA)_2_·H_2_O	80	CF_3_CH_2_OH	35
16	-SO_2_(2-Py)	Ac	Pd(OAc)_2_	Cu(TFA)_2_·H_2_O	100	CF_3_CH_2_OH	45
17	-SO_2_(2-Py)	Ac	Pd(OAc)_2_	Cu(TFA)_2_·H_2_O	120	CF_3_CH_2_OH	46
18	-SO_2_(2-Py)	Ts	Pd(OAc)_2_	Cu(TFA)_2_·H_2_O	100	CF_3_CH_2_OH	trace
19	-SO_2_(2-Py)	Boc	Pd(OAc)_2_	Cu(TFA)_2_·H_2_O	100	CF_3_CH_2_OH	trace
20	-SO_2_(2-Py)	Me	Pd(OAc)_2_	Cu(TFA)_2_·H_2_O	100	CF_3_CH_2_OH	3
21	-SO_2_(2-Py)	Ph	Pd(OAc)_2_	Cu(TFA)_2_·H_2_O	100	CF_3_CH_2_OH	18
22	-SO_2_(2-Py)	Ac	Pd(PPh_3_)_4_	Cu(TFA)_2_·H_2_O	100	CF_3_CH_2_OH	53
23	-SO_2_(2-Py)	Ac	Pd(TFA)_2_	Cu(TFA)_2_·H_2_O	100	CF_3_CH_2_OH	62
24 *^c^*	-SO_2_(2-Py)	Ac	Other [Pd]	Cu(TFA)_2_·H_2_O	100	CF_3_CH_2_OH	trace
25	-SO_2_(2-Py)	Ac	Pd(PPh_3_)_2_Cl_2_	Cu(TFA)_2_·H_2_O	100	CF_3_CH_2_OH	36
26 *^d^*	-SO_2_(2-Py)	Ac	Pd(TFA)_2_	Cu(TFA)_2_·H_2_O	100	CF_3_CH_2_OH	74
27 *^e^*	-SO_2_(2-Py)	Ac	Pd(TFA)_2_	Cu(TFA)_2_·H_2_O	100	CF_3_CH_2_OH	75
28 *^d,f^*	-SO_2_(2-Py)	Ac	Pd(TFA)_2_	Cu(TFA)_2_·H_2_O	100	CF_3_CH_2_OH	16
29 *^d,g^*	-SO_2_(2-Py)	Ac	Pd(TFA)_2_	Cu(TFA)_2_·H_2_O	100	CF_3_CH_2_OH	83
30 *^d,h^*	-SO_2_(2-Py)	Ac	Pd(TFA)_2_	Cu(TFA)_2_·H_2_O	100	CF_3_CH_2_OH	82
31 *^d,i^*	-SO_2_(2-Py)	Ac	Pd(TFA)_2_	Cu(TFA)_2_·H_2_O	100	CF_3_CH_2_OH	83

*^a^* Reaction conditions: **1** (0.5 mmol), catalyst (10 mol%), and oxidant (0.55 mmol) in solvent (2 mL) for 20 h. *^b^* Isolated yield. *^c^* Others, see Table S1 in the Supplementary Materials. *^d^* Pd(TFA)_2_ (15 mol%) was used. *^e^* Pd(TFA)_2_ (30 mol%) was used. *^f^* Cu(TFA)_2_·H_2_O (0.25 mmol) was used. *^g^* Cu(TFA)_2_·H_2_O (1.1 mmol) was used. *^h^* Cu(TFA)_2_·H_2_O (2.0 mmol) was used. *^i^* Under argon atmosphere.

## Data Availability

Not applicable.

## Supplementary Materials

The following supporting information can be downloaded at: https://www.mdpi.com/article/10.3390/molecules28062498/s1, Reference [59] is cited in the Supplementary Materials.
