# Supplementary material for: Synthesis of 5,6-Dihydrophenanthridines via Palladium-Catalyzed Intramolecular Dehydrogenative Coupling of Two Aryl C−H Bonds
Source: Molecules. 2023 Mar 9;28(6):2498. doi: 10.3390/molecules28062498 (PMC10055664; doi:10.3390/molecules28062498)
Supplement: Supplementary file 1 [file molecules-28-02498-s001.zip › molecules-2251241-supplementary.pdf]

Supporting Information

Synthesis of 5,6-Dihydrophenanthridines via Palladium Catalyzed Intramolecular Dehydrogenative Coupling of Two Aryl C–H Bonds

Meng-Yue Wang †, Xue-Qing Zhu †, Bao-Yin Zhao, Hong-Xia Zhang, Yong-Qiang Wang * and Qiong Jia *

Key Laboratory of Synthetic and Natural Functional Molecule Chemistry of Ministry of Education, School of Foreign Languages, College of Chemistry & Materials Science, Northwest University, Xi'an 710069, China
* Correspondence: wangyq@nwu.edu.cn (Y.-Q.W.); jiaqiong@nwu.edu.cn (Q.J.)
† These authors contributed equally to this work.

Table of Contents

1. **General information··························S1**
2. **Condition optimization for preparation of product 2··························S2**
3. **Experimental procedure for the KIE study··························S4**
4. **Spectral copies of substrates ^{1}H NMR and ^{13}C NMR··························S5**
5. **Spectral copies of products ^{1}H NMR and ^{13}C NMR··························S25**
6. **Spectral copies of 3-7 ^{1}H NMR and ^{13}C NMR··························S45**
7. **X-ray Crystal Structure of Compounds 2a··························S50**

1. General information

All reactions were carried out under an air atmosphere. Unless noted otherwise, commercially available chemicals were used without further purification. Flash chromatography was performed with silica gel (200-300 mesh). Oil bath served as the heat source. NMR spectra were acquired on Bruker 400 MHz (^{1}H at 400 MHz, ^{13}C at 101 MHz) or Jeol 400 MHz (^{1}H at 400 MHz, ^{13}C at 100 MHz). NMR spectra were recorded in $CDCl_3$ (TMS, δ = 0.00 ppm for ^{1}H NMR and δ = 77.10 ppm for ^{13}C NMR) or DMSO-d_6 (δ = 2.50 ppm for ^{1}H NMR and δ = 39.52 ppm for ^{13}C NMR) or CD_3OD (δ = 3.31 ppm for ^{1}H NMR and δ = 49.00 ppm for ^{13}C NMR) using the solvent residue peaks as the internal references. Coupling constants were reported in Hertz (Hz). Data for ^{1}H NMR spectra were reported as follows: chemical shift (ppm, referenced to protium, s = singlet, d = doublet, t = triplet, q = quartet, dd = doublet of doublets, td = triplet of doublets, ddd = doublet of doublet of doublets, m = multiplet, coupling constant (Hz), and integration). Infrared (IR) data were acquired on a Bruker Invenio-RFT-IR spectrometer. Absorbance frequencies are reported in reciprocal centimeters (cm^{-1}). Mass spectra were acquired on a BrukerDaltonics S2 MicroTof-Q II mass spectrometer. X-ray crystal structure analyses were measured on Bruker Smart APEXIICCD instrument using Mo-Kα radiation. The structures were solved and refined using the SHELXTL software package.

2. Condition optimization for preparation of product **2**

Table S1. Optimization of conditions for the synthesis of 5,6-dihydrophenanthridine

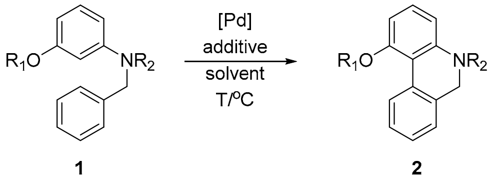

	R^1	R^2	Pd cat. (equiv)	Additive (equiv)	T /°C	Solvent	Yield (%)[a]
1	-H	Ac	$Pd(OAc)_2$ (0.1)	/	70	DMSO	0
2	$-CONMe_2$	Ac	$Pd(OAc)_2$ (0.1)	/	70	DMSO	trace
3	-CO(2-Py)	Ac	$Pd(OAc)_2$ (0.1)	/	70	DMSO	trace
4	$-SO_2$(2-Py)	Ac	$Pd(OAc)_2$ (0.1)	/	70	DMSO	9
5	$-SO_2$(2-Py)	Ac	$Pd(OAc)_2$ (0.1)	/	70	DCE	5
6	$-SO_2$(2-Py)	Ac	$Pd(OAc)_2$ (0.1)	/	70	HFIP	11
7	$-SO_2$(2-Py)	Ac	$Pd(OAc)_2$ (0.1)	/	70	CF_3CH_2OH	12
8	$-SO_2$(2-Py)	Ac	$Pd(OAc)_2$ (0.1)	/	70	$CF_2HCF_2CH_2OH$	4
9	$-SO_2$(2-Py)	Ac	$Pd(OAc)_2$ (0.1)	/	70	CF_2HCH_2OH	5
10	$-SO_2$(2-Py)	Ac	$Pd(OAc)_2$ (0.1)	/	70	EA	2
11	$-SO_2$(2-Py)	Ac	$Pd(OAc)_2$ (0.1)	/	70	PhMe	3
12	$-SO_2$(2-Py)	Ac	$Pd(OAc)_2$ (0.1)	/	70	Other solvents	N.R.
13	$-SO_2$(2-Py)	Ac	$Pd(OAc)_2$ (0.1)	$Cu(TFA)_2\cdot H_2O$ (1.1)	70	CF_3CH_2OH	31
14	$-SO_2$(2-Py)	Ac	$Pd(OAc)_2$ (0.1)	$Cu(OAc)_2\cdot H_2O$ (1.1)	70	CF_3CH_2OH	22
15	$-SO_2$(2-Py)	Ac	$Pd(OAc)_2$ (0.1)	$Cu(OAc)_2$ (1.1)	70	CF_3CH_2OH	21
16	$-SO_2$(2-Py)	Ac	$Pd(OAc)_2$ (0.1)	CuOAc (1.1)	70	CF_3CH_2OH	11
17	$-SO_2$(2-Py)	Ac	$Pd(OAc)_2$ (0.1)	CuI (1.1)	70	CF_3CH_2OH	10
18	$-SO_2$(2-Py)	Ac	$Pd(OAc)_2$ (0.1)	CuO (1.1)	70	CF_3CH_2OH	12
19	$-SO_2$(2-Py)	Ac	$Pd(OAc)_2$ (0.1)	$CuSO_4$ (1.1)	70	CF_3CH_2OH	18
20	$-SO_2$(2-Py)	Ac	$Pd(OAc)_2$ (0.1)	$CuF_2\cdot 2H_2O$ (1.1)	70	CF_3CH_2OH	29
21	$-SO_2$(2-Py)	Ac	$Pd(OAc)_2$ (0.1)	AgTFA (1.1)	70	CF_3CH_2OH	9
22	$-SO_2$(2-Py)	Ac	$Pd(OAc)_2$ (0.1)	$AgNO_3$ (1.1)	70	CF_3CH_2OH	8

23	$-SO_2(2\text{-}Py)$	Ac	$Pd(OAc)_2$ (0.1)	Other additives	70	CF_3CH_2OH	N.R.
24[b]	$-SO_2(2\text{-}Py)$	Ac	$Pd(OAc)_2$ (0.1)	-	70	CF_3CH_2OH	8
25	$-SO_2(2\text{-}Py)$	Ac	$Pd(OAc)_2$ (0.1)	$Cu(TFA)_2{\cdot}H_2O$ (1.1)	40	CF_3CH_2OH	N.R.
26	$-SO_2(2\text{-}Py)$	Ac	$Pd(OAc)_2$ (0.1)	$Cu(TFA)_2{\cdot}H_2O$ (1.1)	60	CF_3CH_2OH	23
27	$-SO_2(2\text{-}Py)$	Ac	$Pd(OAc)_2$ (0.1)	$Cu(TFA)_2{\cdot}H_2O$ (1.1)	80	CF_3CH_2OH	35
28	$-SO_2(2\text{-}Py)$	Ac	$Pd(OAc)_2$ (0.1)	$Cu(TFA)_2{\cdot}H_2O$ (1.1)	100	CF_3CH_2OH	45
29	$-SO_2(2\text{-}Py)$	Ac	$Pd(OAc)_2$ (0.1)	$Cu(TFA)_2{\cdot}H_2O$ (1.1)	120	CF_3CH_2OH	46
30	$-SO_2(2\text{-}Py)$	Ts	$Pd(OAc)_2$ (0.1)	$Cu(TFA)_2{\cdot}H_2O$ (1.1)	100	CF_3CH_2OH	trace
31	$-SO_2(2\text{-}Py)$	Boc	$Pd(OAc)_2$ (0.1)	$Cu(TFA)_2{\cdot}H_2O$ (1.1)	100	CF_3CH_2OH	trace
32	$-SO_2(2\text{-}Py)$	Me	$Pd(OAc)_2$ (0.1)	$Cu(TFA)_2{\cdot}H_2O$ (1.1)	100	CF_3CH_2OH	3
33	$-SO_2(2\text{-}Py)$	Ph	$Pd(OAc)_2$ (0.1)	$Cu(TFA)_2{\cdot}H_2O$ (1.1)	100	CF_3CH_2OH	18
34	$-SO_2(2\text{-}Py)$	Ac	$Pd(PPh_3)_4$ (0.1)	$Cu(TFA)_2{\cdot}H_2O$ (1.1)	100	CF_3CH_2OH	53
35	$-SO_2(2\text{-}Py)$	Ac	$Pd(TFA)_2$ (0.1)	$Cu(TFA)_2{\cdot}H_2O$ (1.1)	100	CF_3CH_2OH	62
36	$-SO_2(2\text{-}Py)$	Ac	$Pd_2(dba)_3$ (0.1)	$Cu(TFA)_2{\cdot}H_2O$ (1.1)	100	CF_3CH_2OH	trace
37	$-SO_2(2\text{-}Py)$	Ac	$Pd(OH)_2$ (0.1)	$Cu(TFA)_2{\cdot}H_2O$ (1.1)	100	CF_3CH_2OH	N.R.
38	$-SO_2(2\text{-}Py)$	Ac	PdI_2 (0.1)	$Cu(TFA)_2{\cdot}H_2O$ (1.1)	100	CF_3CH_2OH	N.R.
39	$-SO_2(2\text{-}Py)$	Ac	$Pd(PPh_3)_2Cl_2$ (0.1)	$Cu(TFA)_2{\cdot}H_2O$ (1.1)	100	CF_3CH_2OH	36
40	$-SO_2(2\text{-}Py)$	Ac	$Pd(TFA)_2$ (0.15)	$Cu(TFA)_2{\cdot}H_2O$ (1.1)	100	CF_3CH_2OH	74
41	$-SO_2(2\text{-}Py)$	Ac	$Pd(TFA)_2$ (0.3)	$Cu(TFA)_2{\cdot}H_2O$ (1.1)	100	CF_3CH_2OH	75
42	$-SO_2(2\text{-}Py)$	Ac	/	$Cu(TFA)_2{\cdot}H_2O$ (1.1)	100	CF_3CH_2OH	/
43	$-SO_2(2\text{-}Py)$	Ac	$Pd(TFA)_2$ (0.15)	$Cu(TFA)_2{\cdot}H_2O$ (0.5)	100	CF_3CH_2OH	16
44	$-SO_2(2\text{-}Py)$	Ac	$Pd(TFA)_2$ (0.15)	$Cu(TFA)_2{\cdot}$H2O (2.2)	100	CF_3CH_2OH	83
45	$-SO_2(2\text{-}Py)$	Ac	$Pd(TFA)_2$ (0.15)	$Cu(TFA)_2{\cdot}H_2O$ (4.0)	100	CF_3CH_2OH	82
46[b]	$-SO_2(2\text{-}Py)$	Ac	$Pd(TFA)_2$ (0.15)	$Cu(TFA)_2{\cdot}$H2O (2.2)	100	CF_3CH_2OH	83

[a] Isolated yield. [b] Under argon atmosphere.

3. Experimental procedure for the KIE study

3.1 **Starting material:** The synthesis of [D_7]-benzyl bromide was synthesized according to the literature [59]. The synthesis of [D_7]-**1a** and [D_6]-**2a** were the same as the synthesis of above mentioned materials **1a** and **2a**.

KIE experiment: We use **1a** and [D_7]-**1a** as starting materials and two parallel reacting at standard conditions. The conversion was measured by ^{1}H NMR analysis for three times (1.5 h, 2.0 h, 3.0 h) to compare the initial reaction rates.

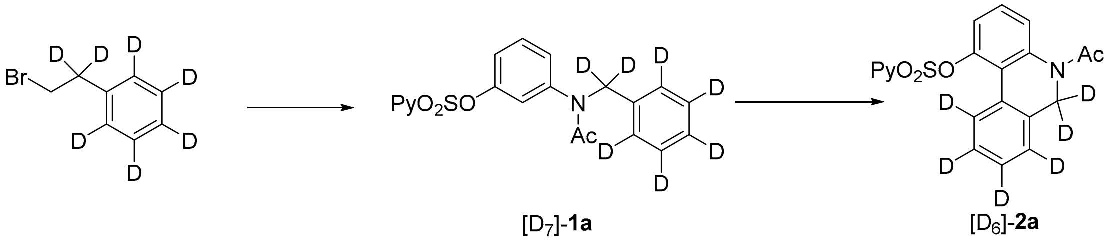

Table S2 **Conversion (%) of the reaction of 1a** (7-H or 7-D).

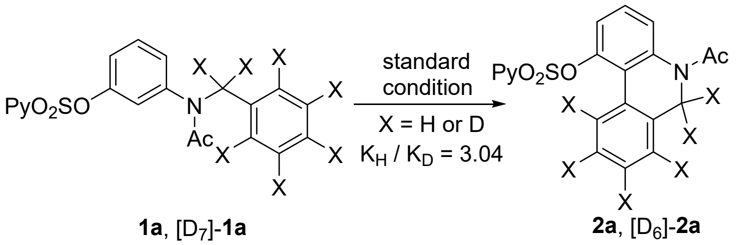

reaction time (h)	H-Conversion (%)	D-Conversion (%)
0	0	0
1.5	3.18	1.47
2.0	3.66	2.91
3.0	9.09	3.67

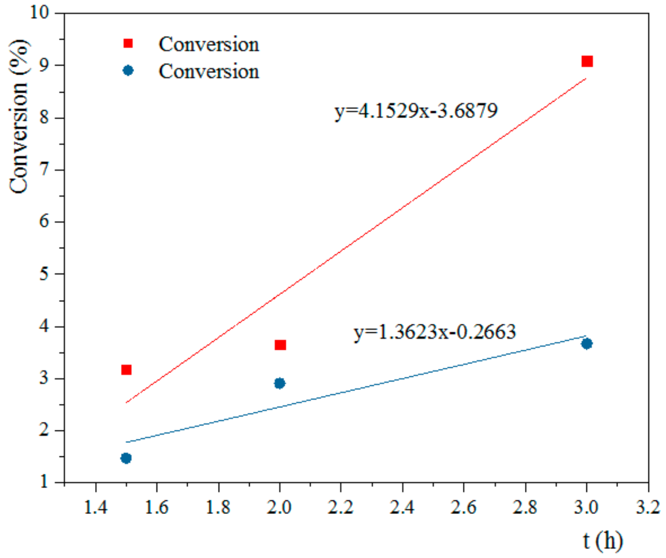

Figure S1. Conversion (%) of the reaction of **1a** (7-H or 7-D).

4. Spectral copies of substrates ^{1}H NMR and ^{13}C NMR

1^{1}H NMR spectra of compound **1a**

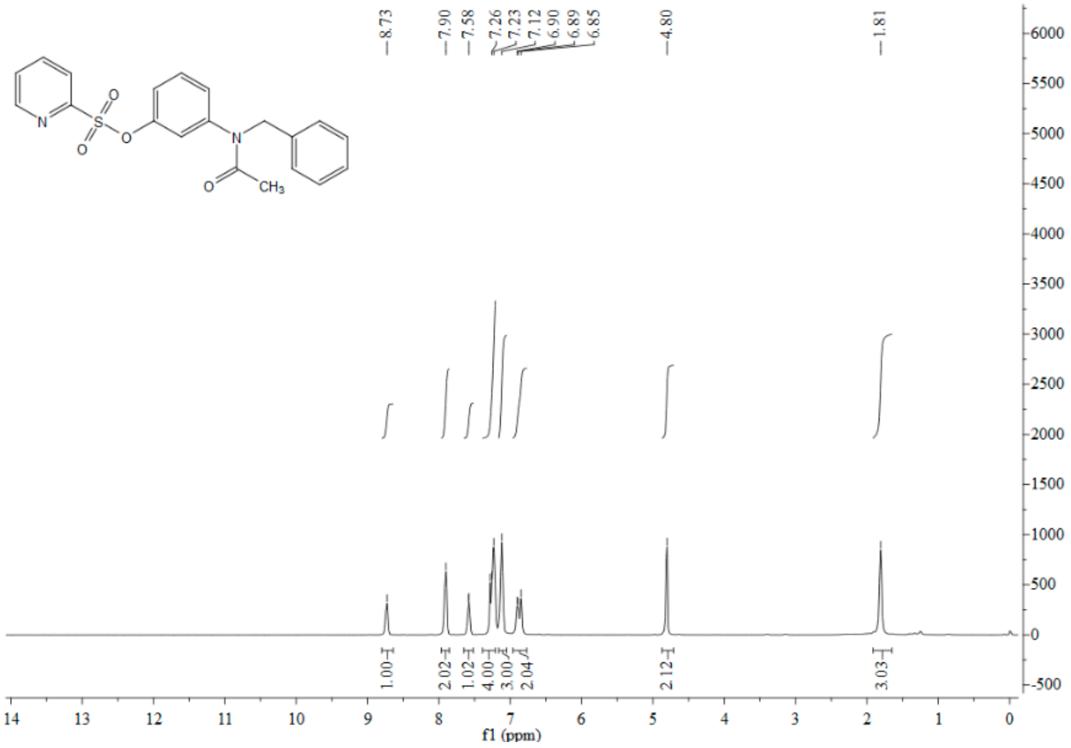

^{13}C NMR spectra of compound **1a**

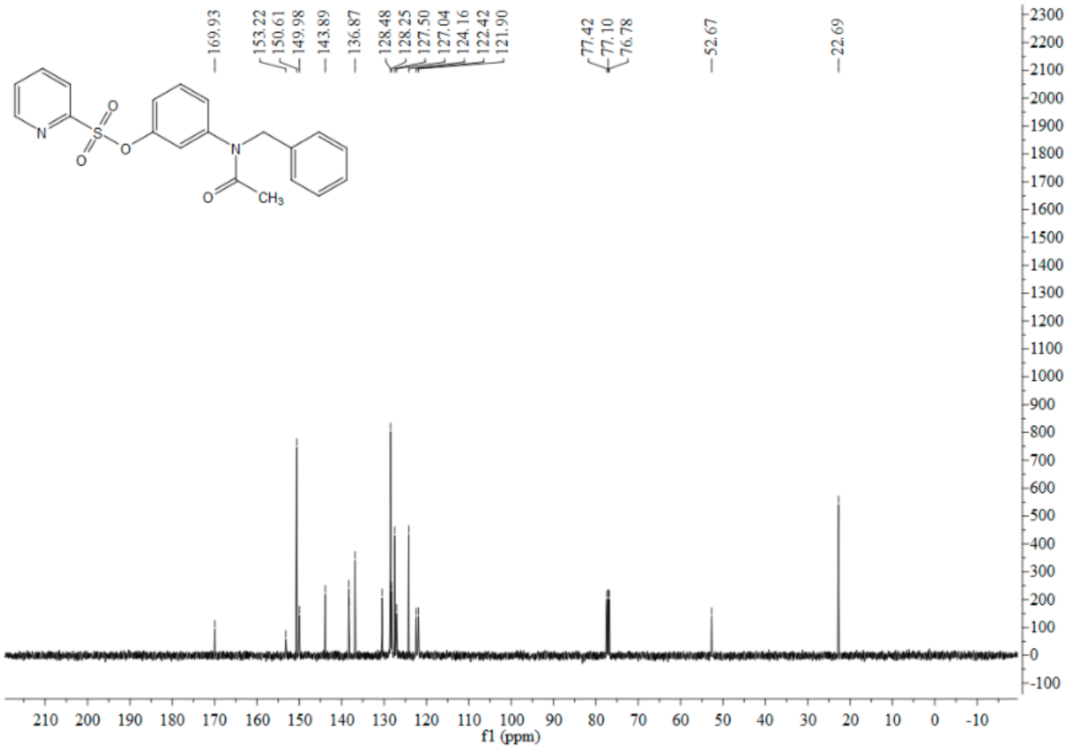

^{1}H NMR spectra of compound **1b**

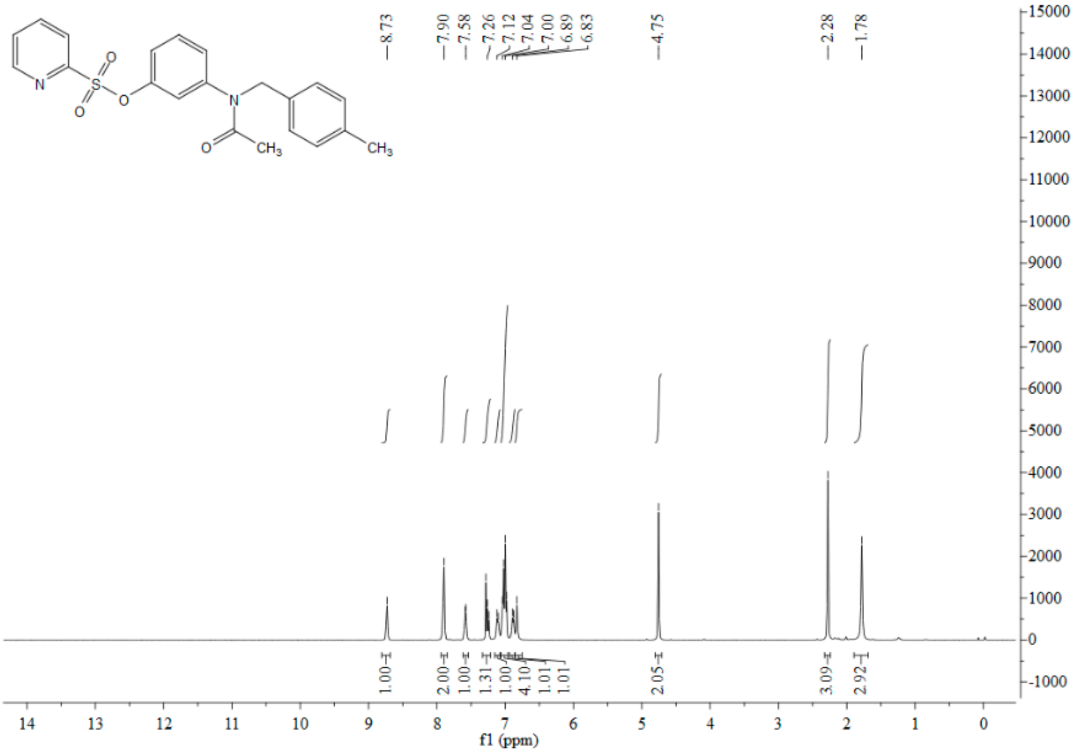

^{13}C NMR spectra of compound **1b**

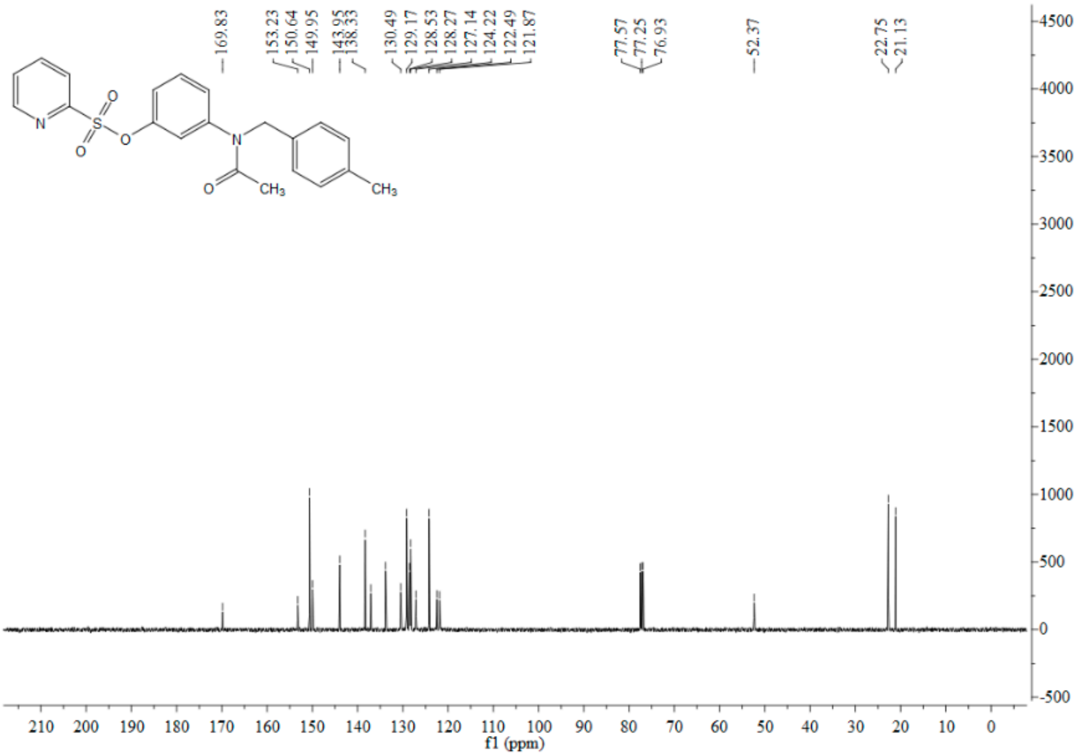

^{13}C NMR spectra of compound **1c**

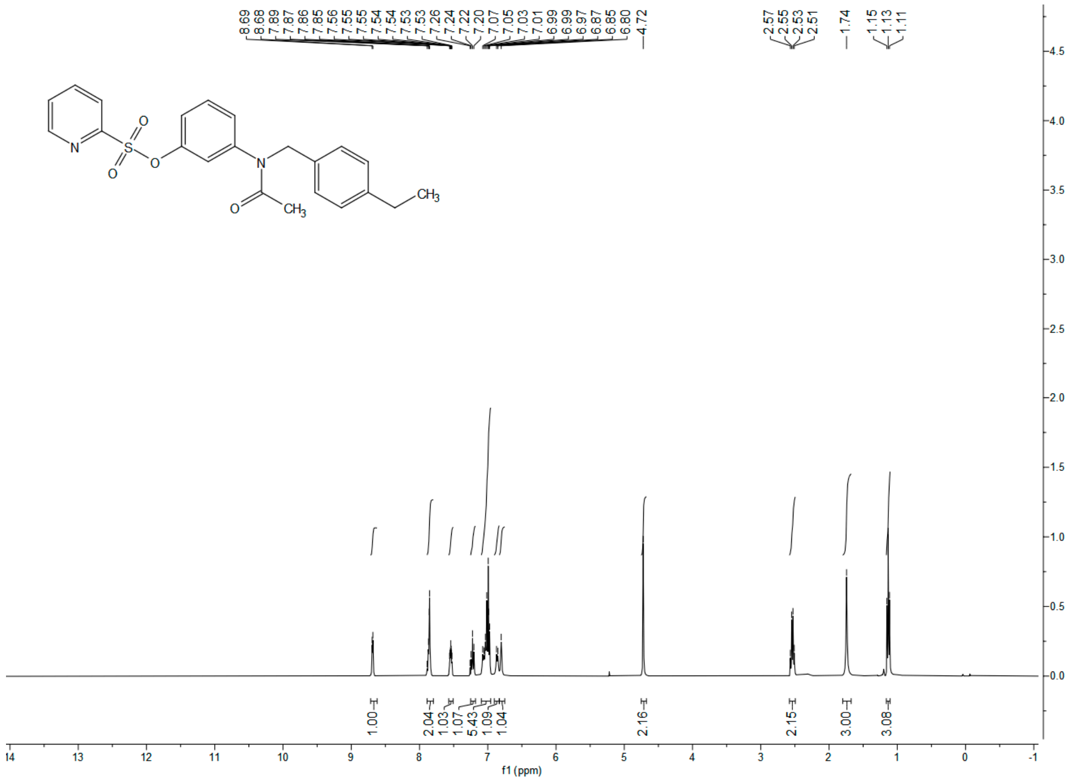

^{13}C NMR spectra of compound **1c**

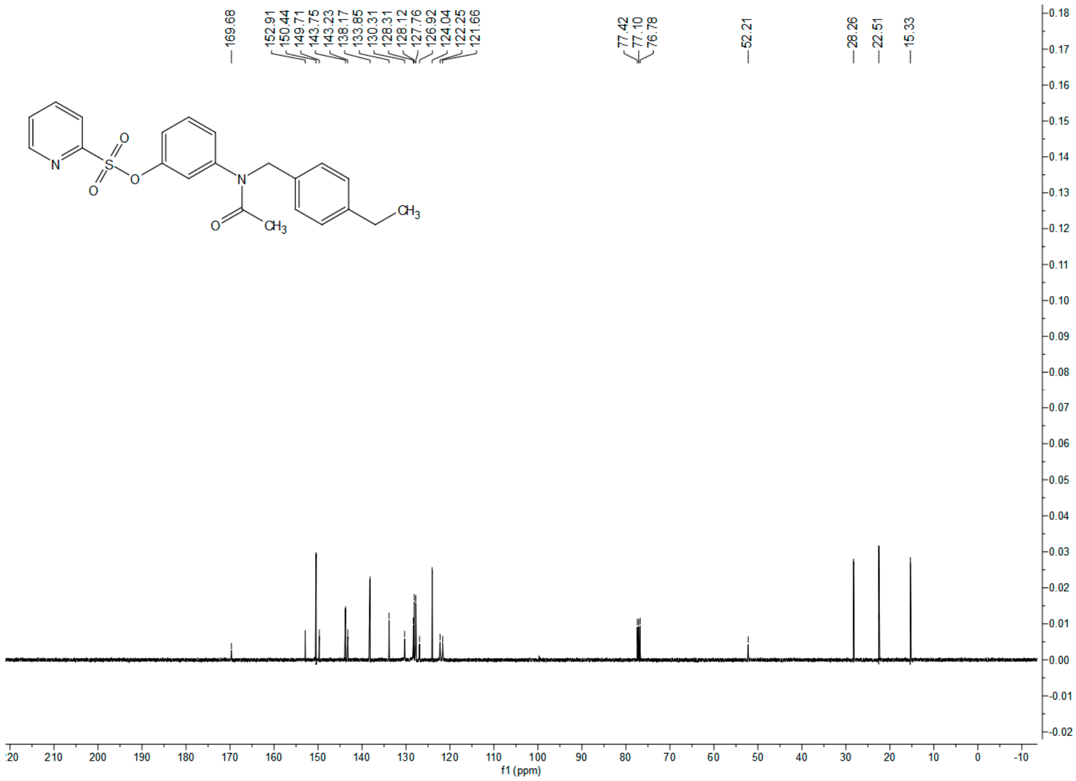

^{1}H NMR spectra of compound **1d**

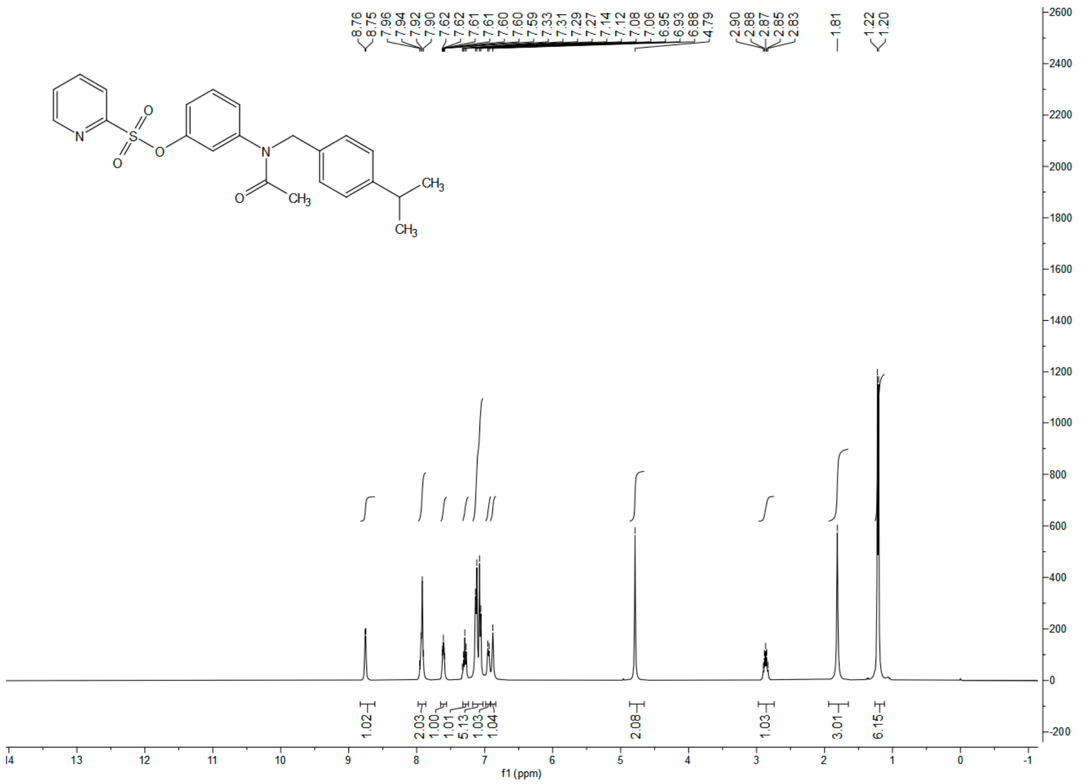

^{13}C NMR spectra of compound **1d**

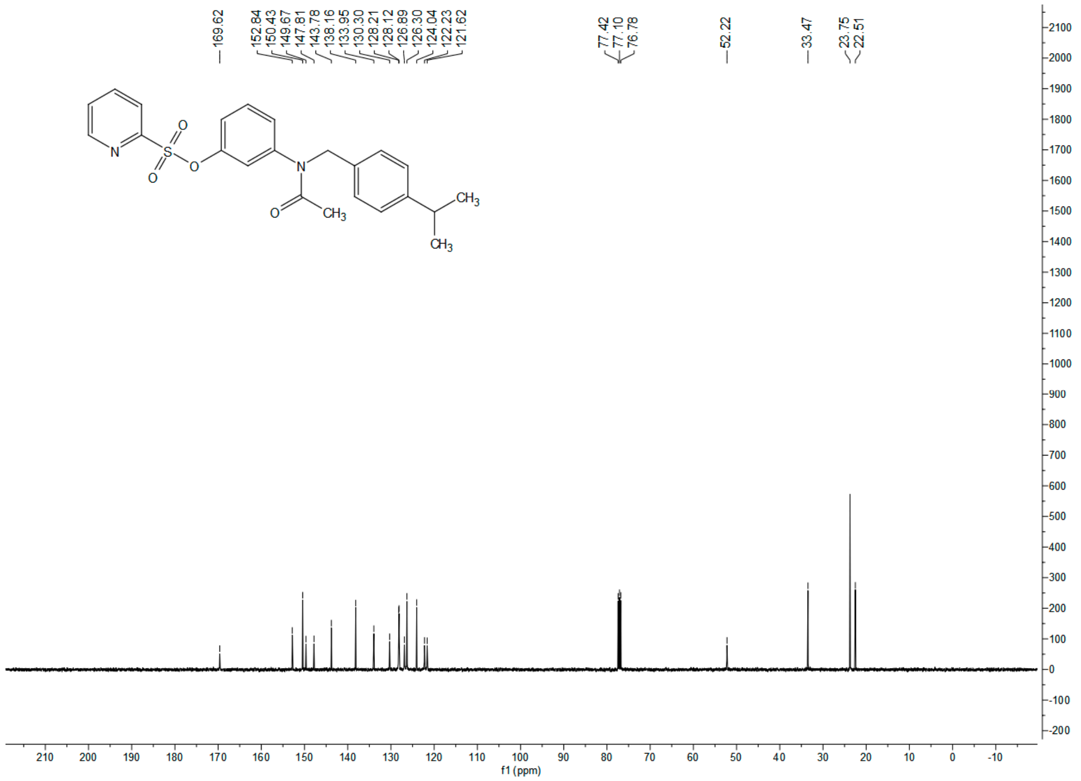

^{1}H NMR spectra of compound **1e**

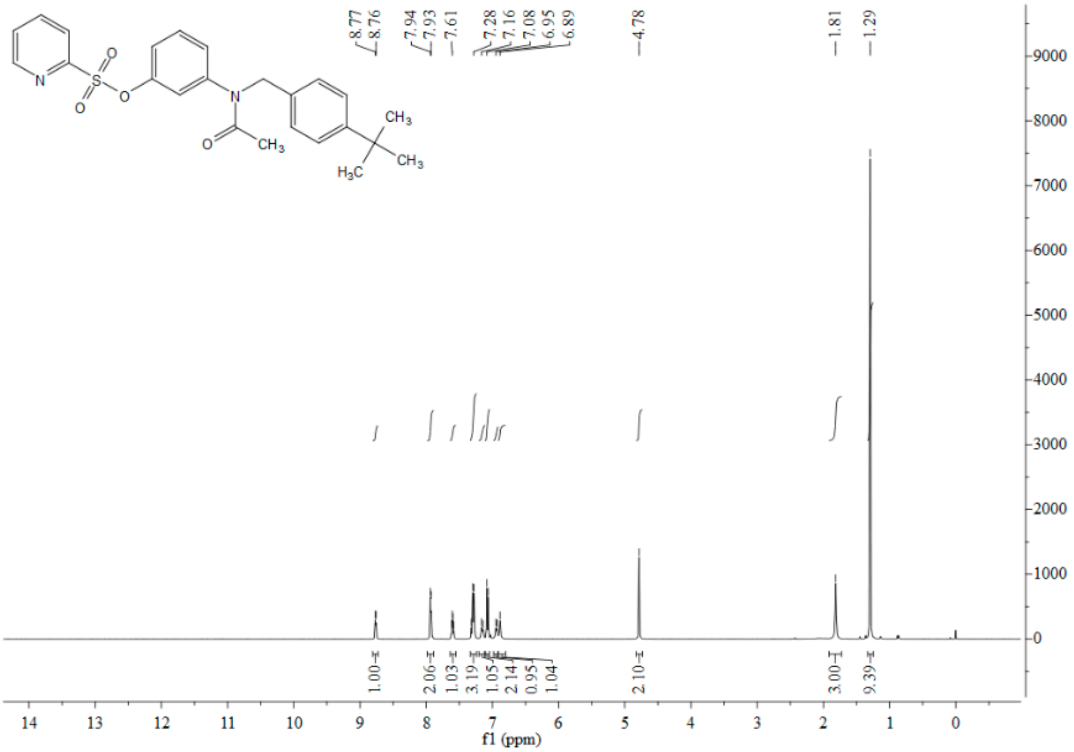

^{13}C NMR spectra of compound **1e**

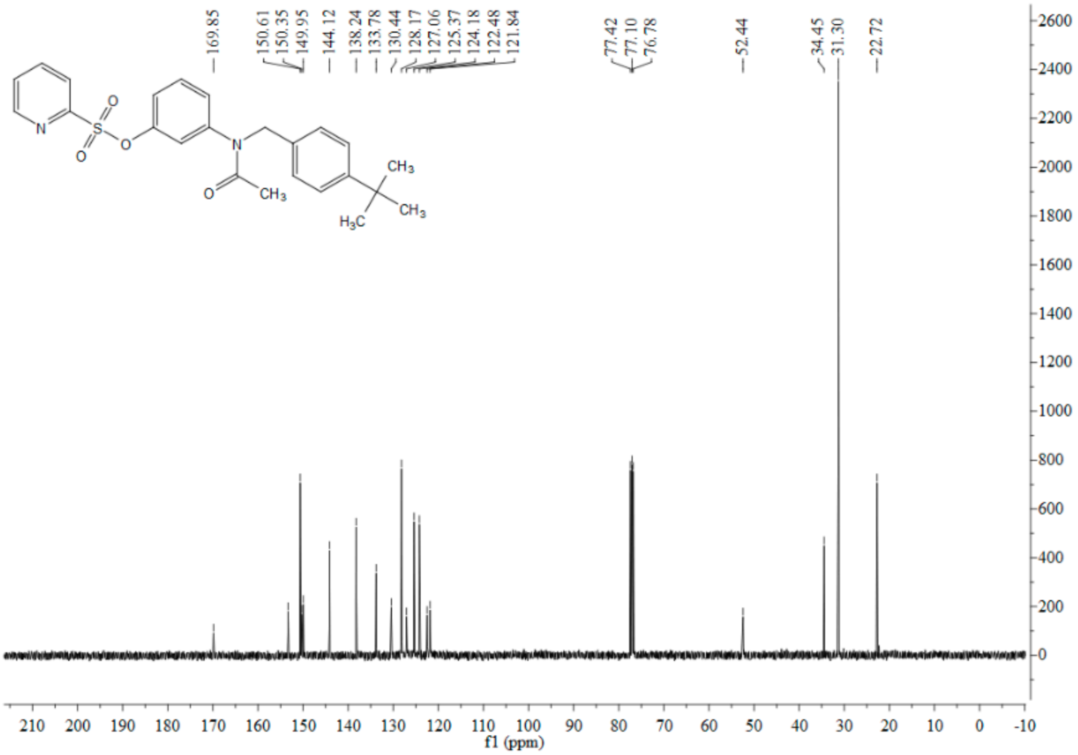

^{1}H NMR spectra of compound **1f**

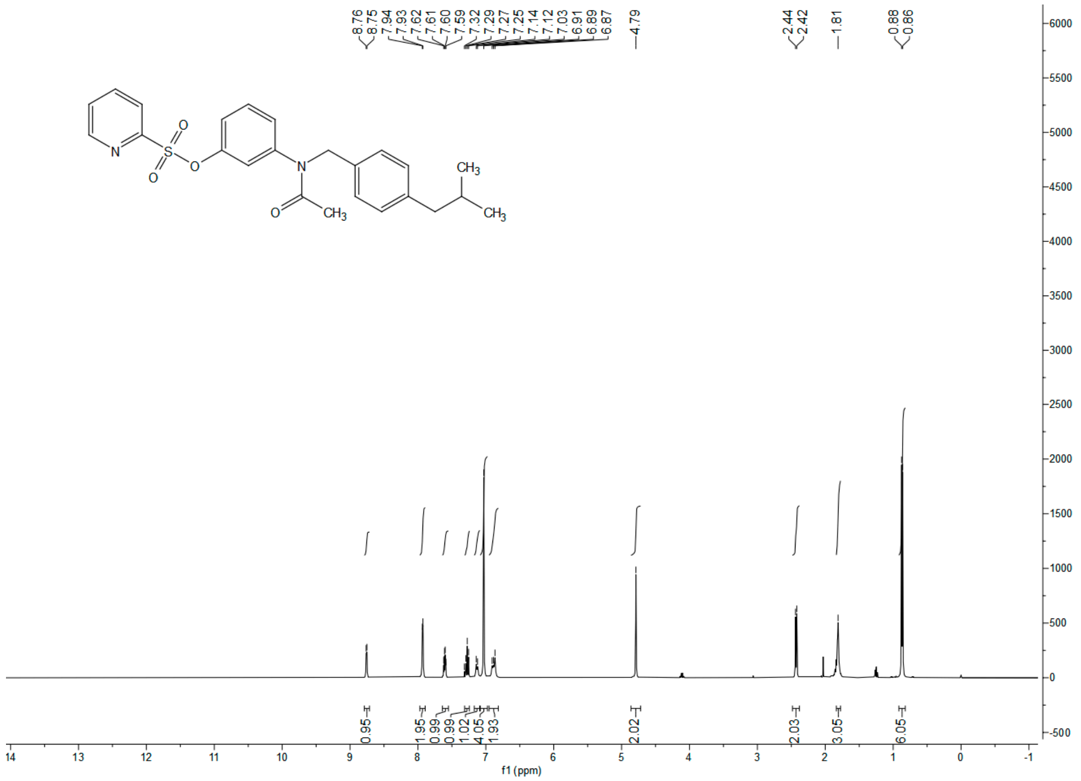

^{13}C NMR spectra of compound **1f**

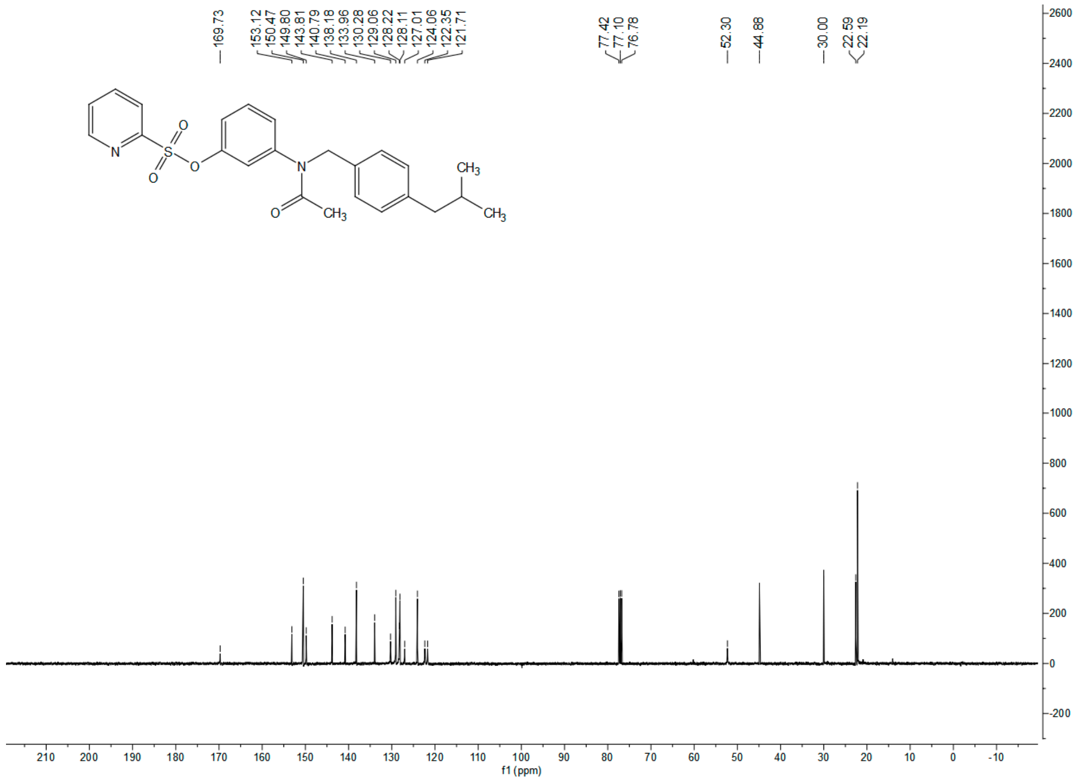

^{1}H NMR spectra of compound **1g**

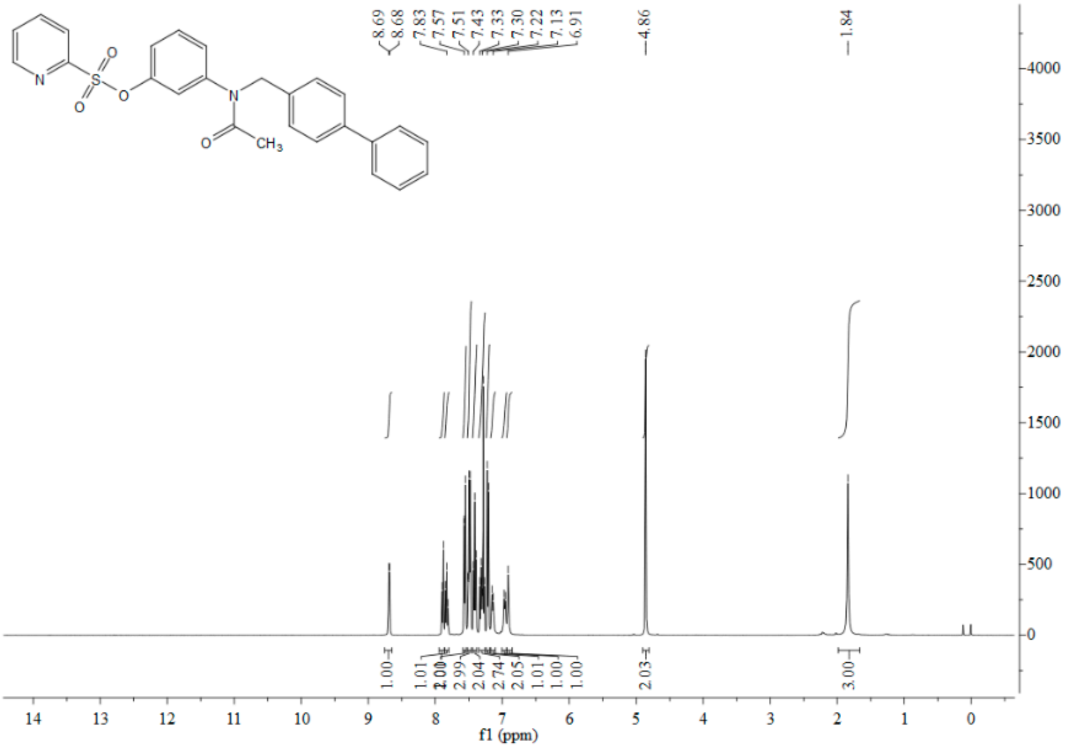

^{13}C NMR spectra of compound **1g**

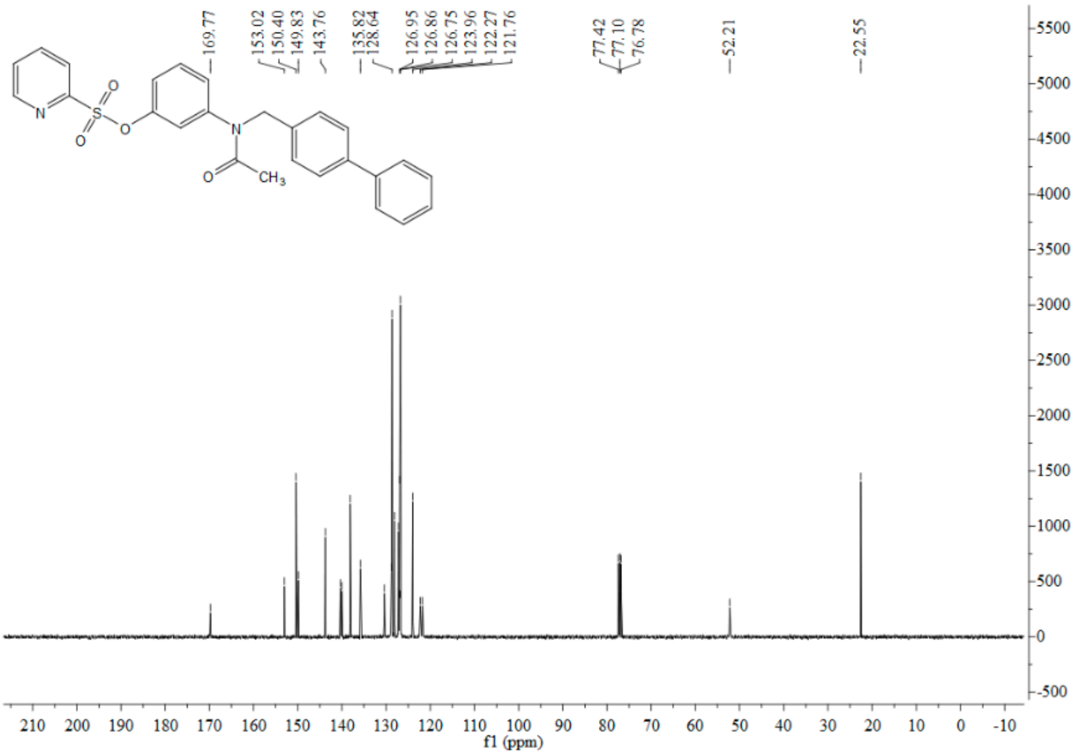

^{1}H NMR spectra of compound **1h**

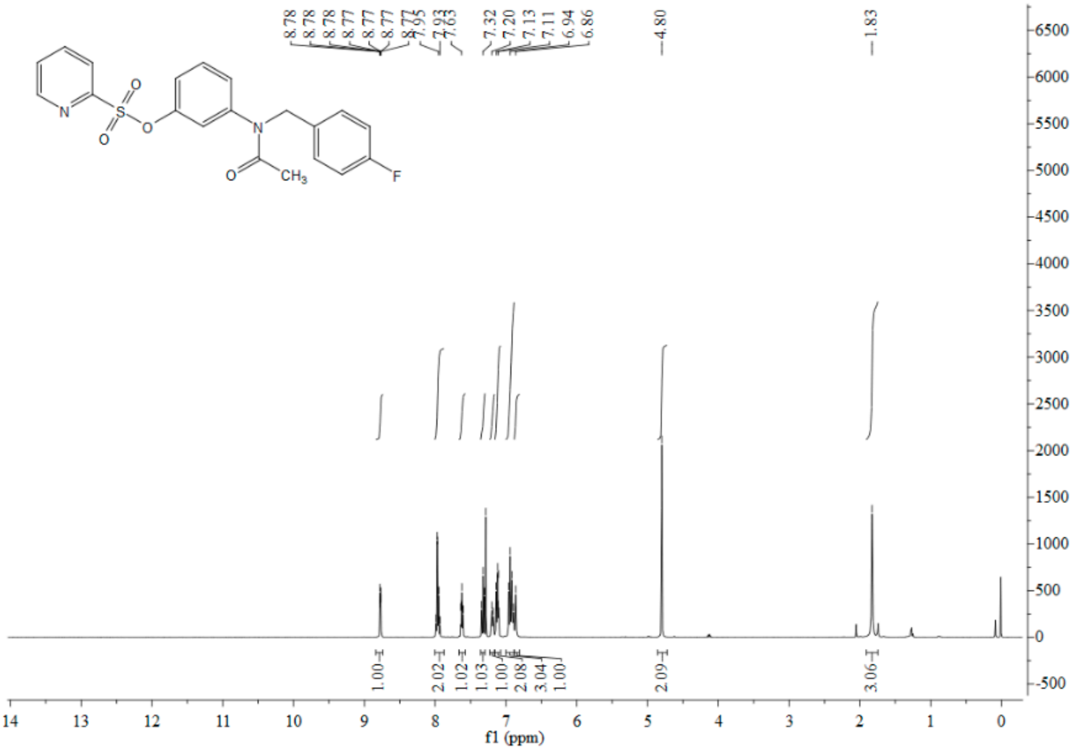

^{13}C NMR spectra of compound **1h**

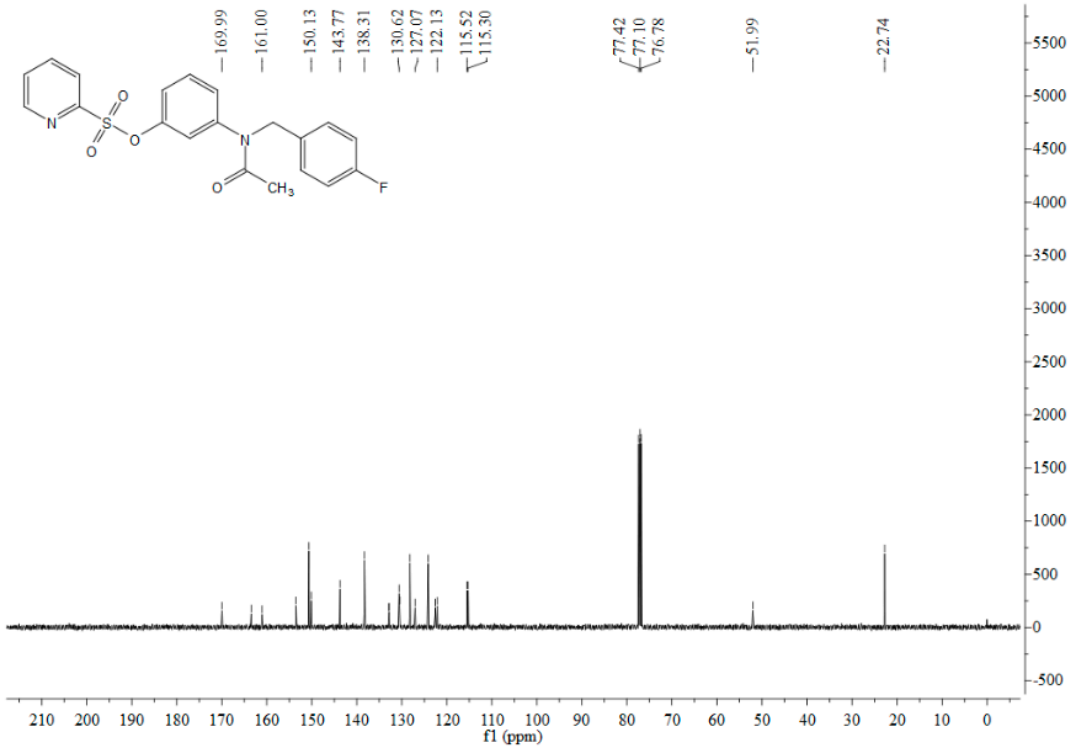

^{1}H NMR spectra of compound **1i**

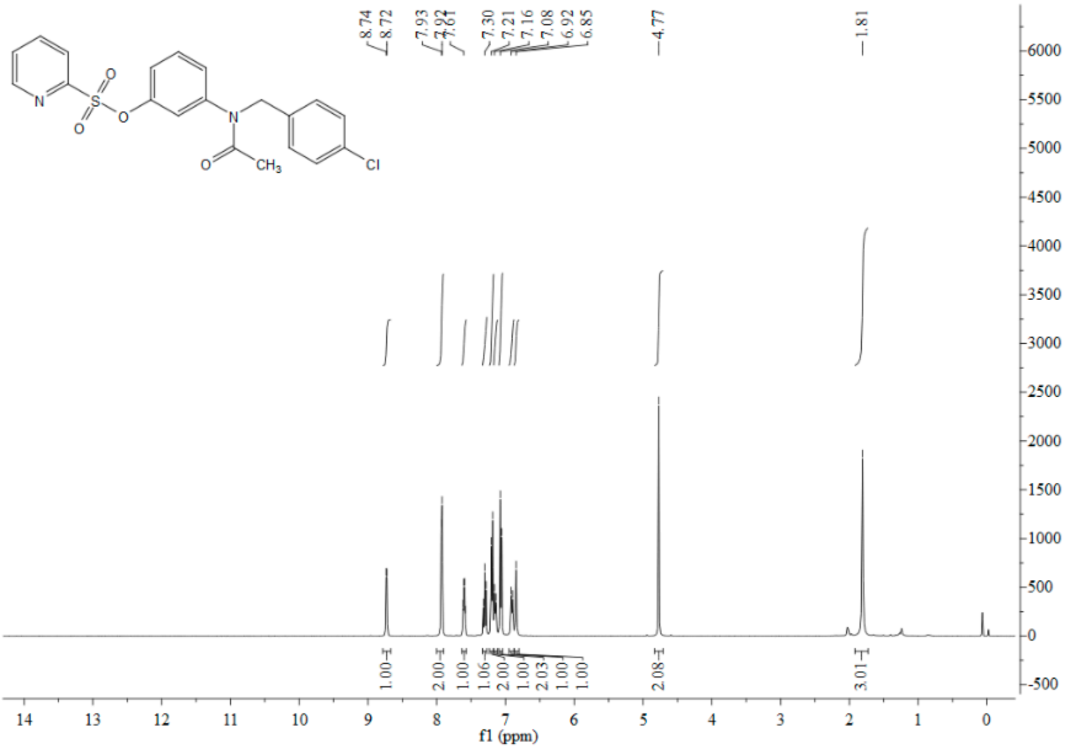

^{13}C NMR spectra of compound **1i**

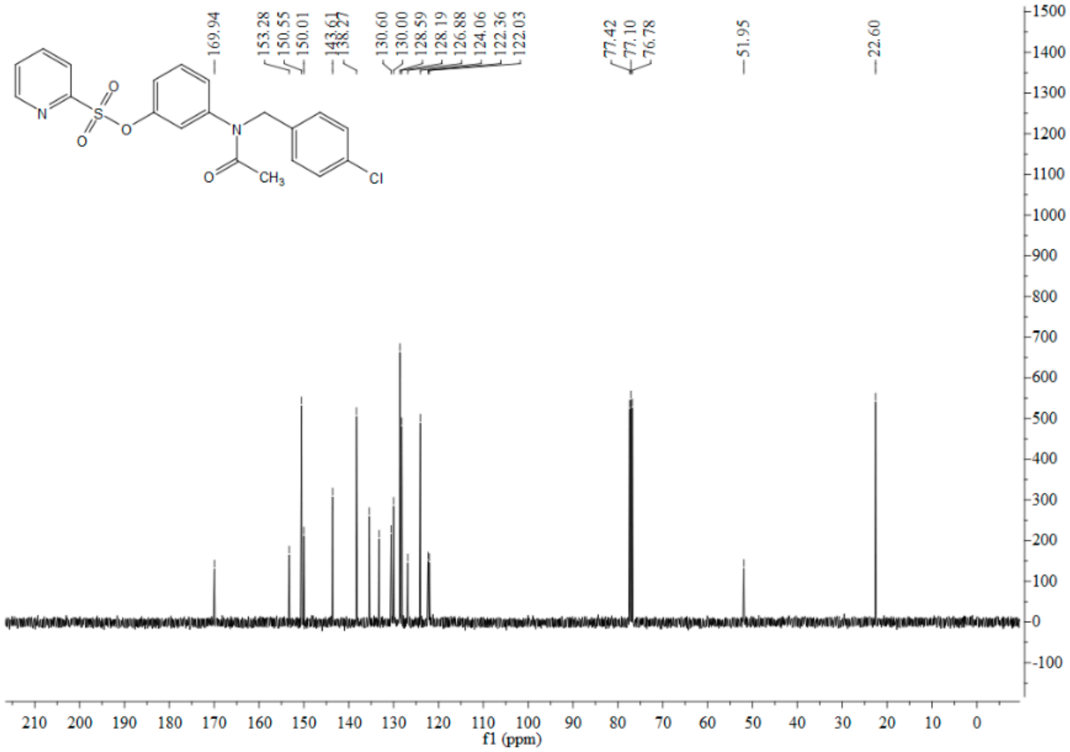

^{1}H NMR spectra of compound **1j**

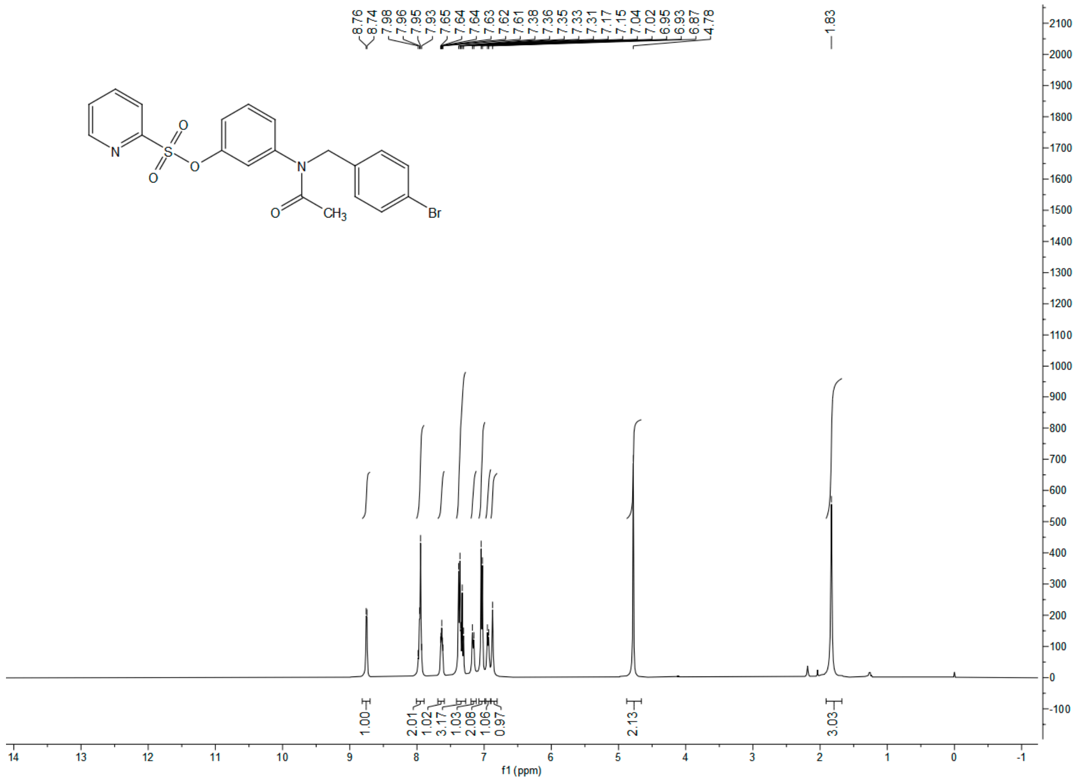

^{13}C NMR spectra of compound **1j**

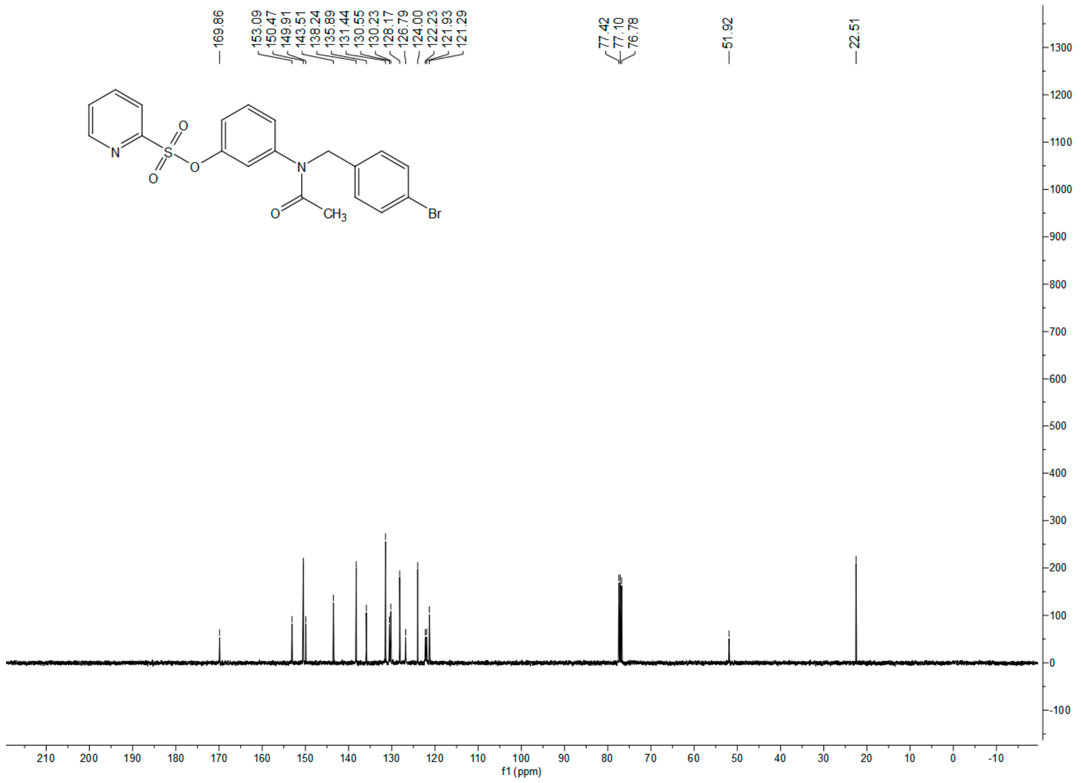

^{1}H NMR spectra of compound **1k**

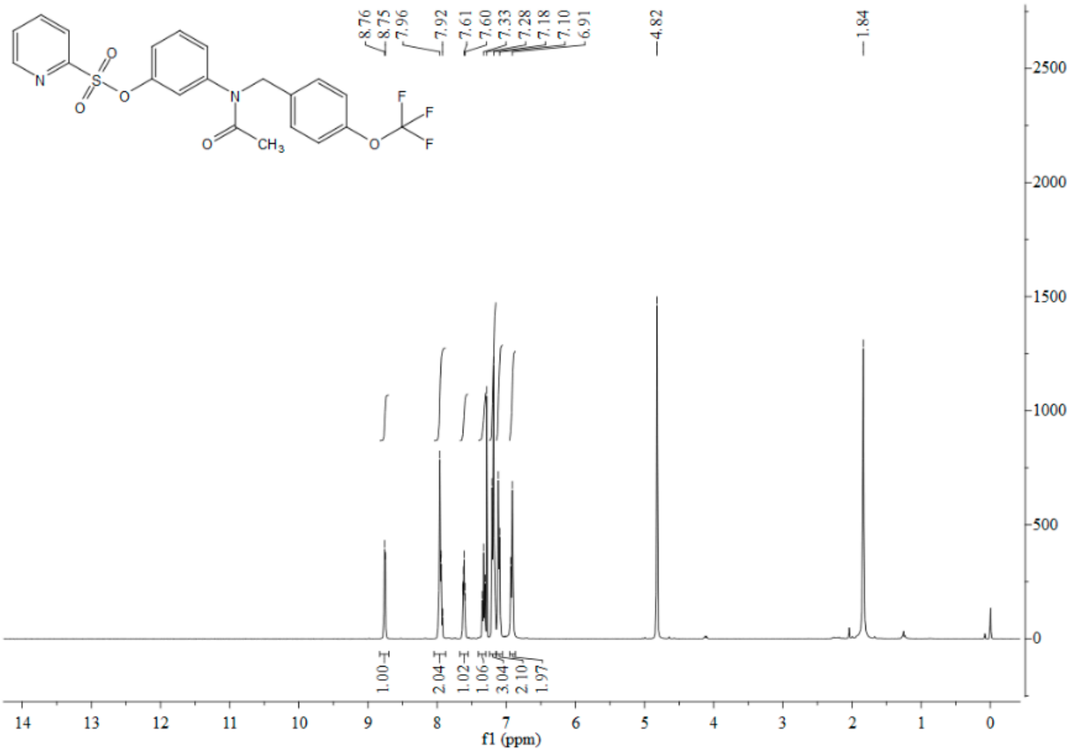

^{13}C NMR spectra of compound **1k**

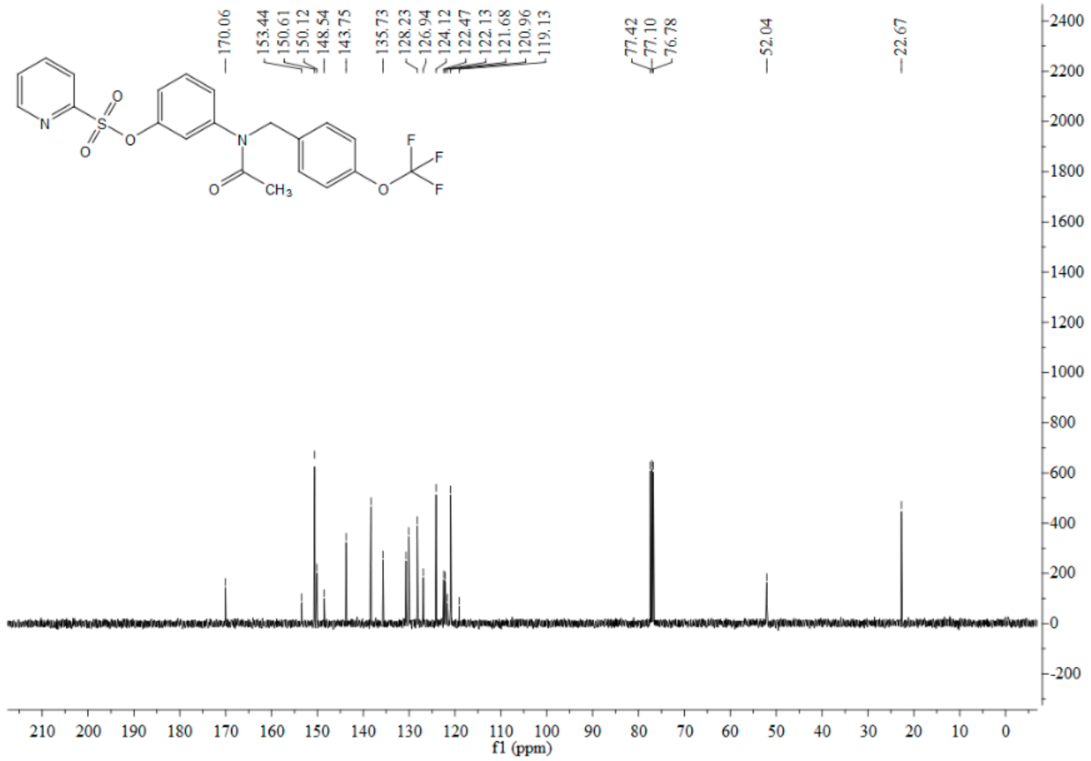

^{1}H NMR spectra of compound **1l**

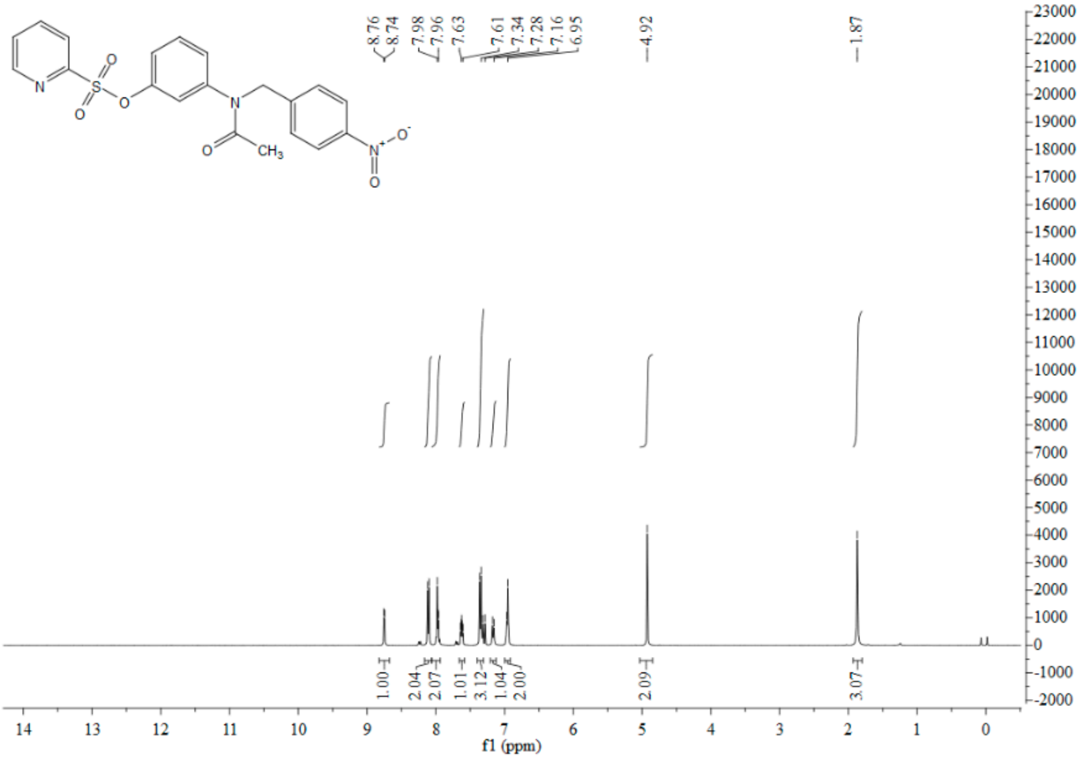

^{13}C NMR spectra of compound **1l**

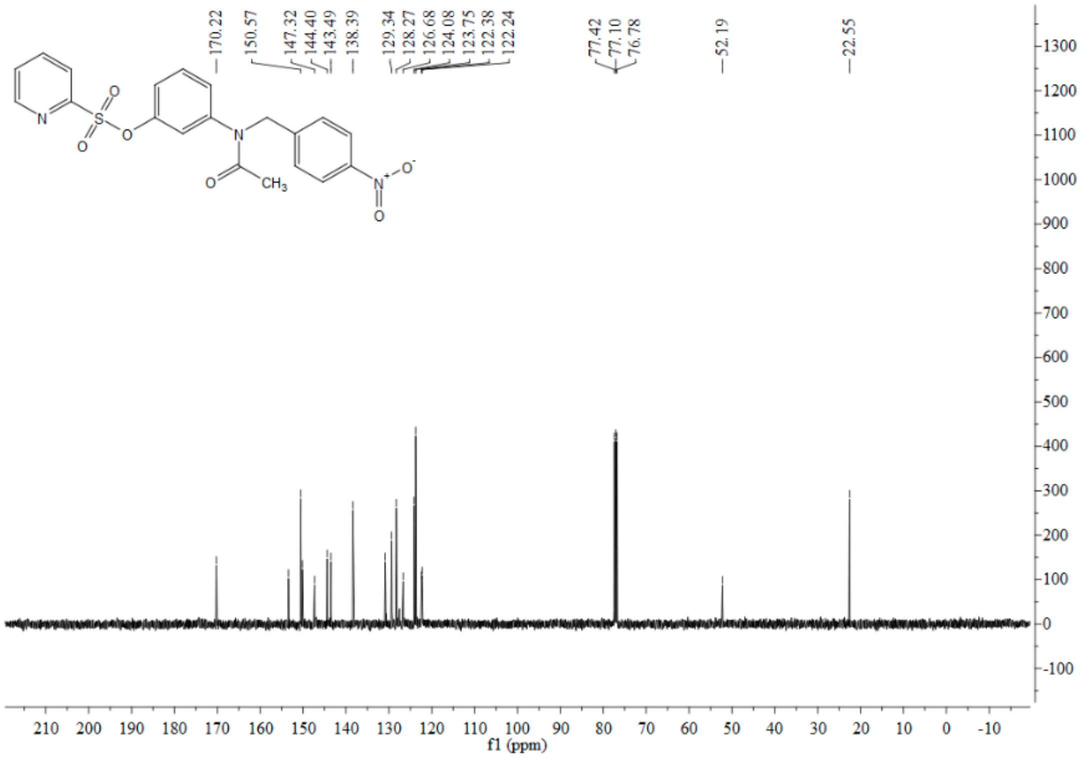

^{1}H NMR spectra of compound **1m**

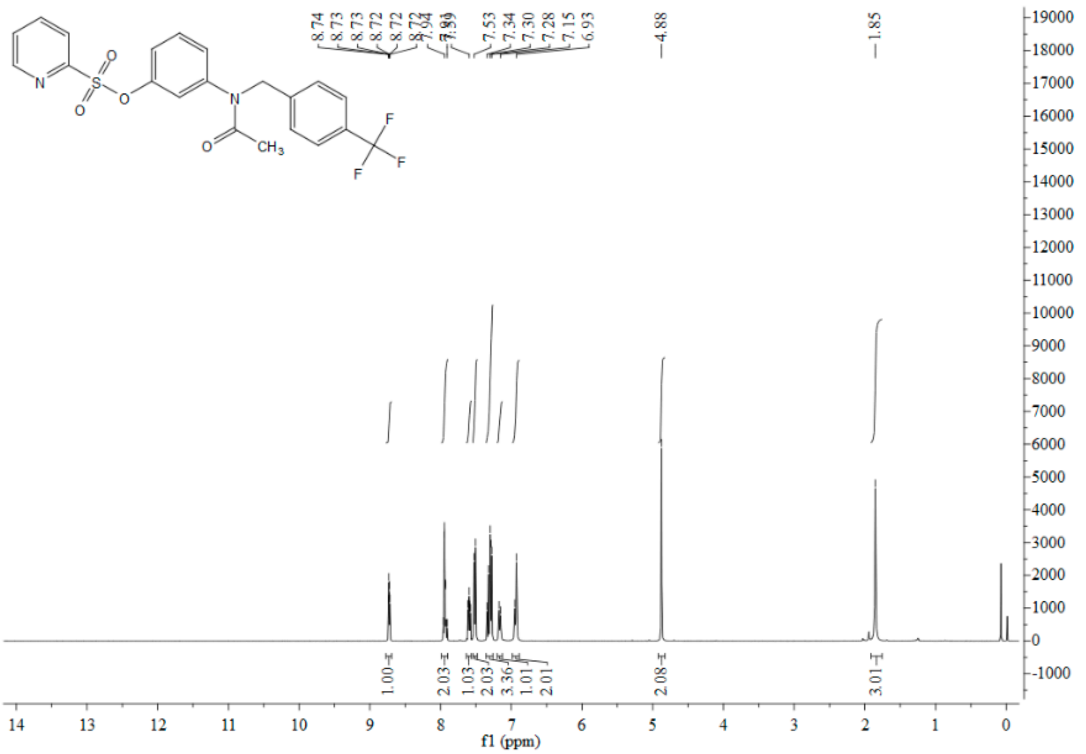

^{13}C NMR spectra of compound **1m**

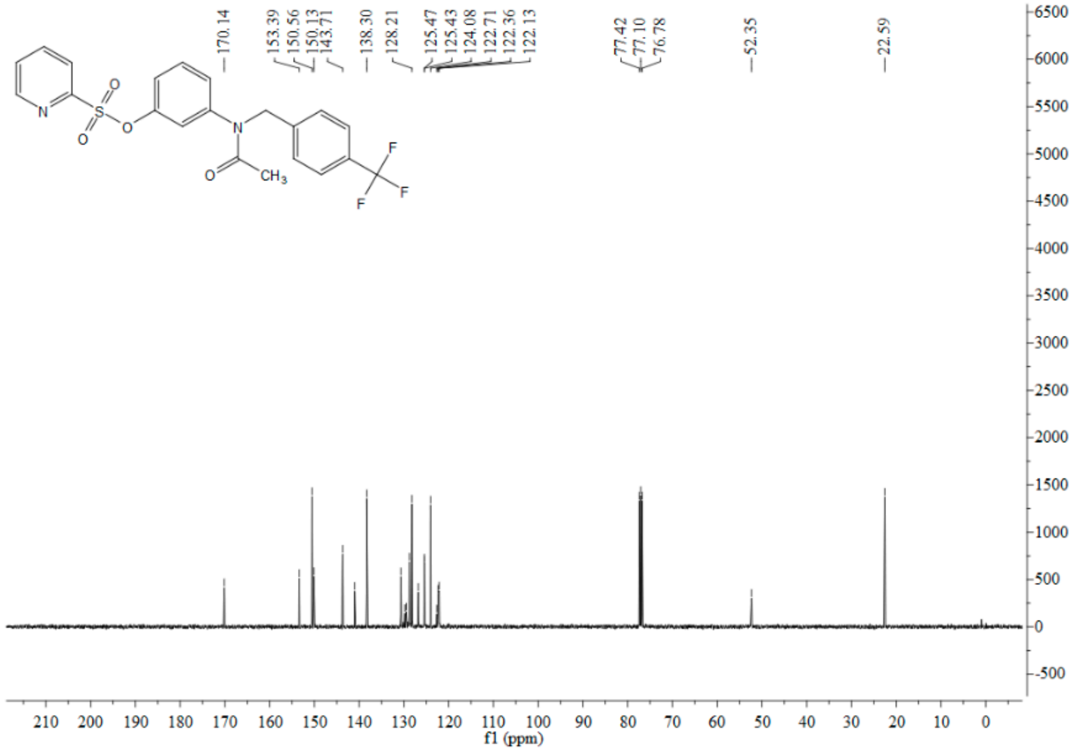

^{1}H NMR spectra of compound **1n**

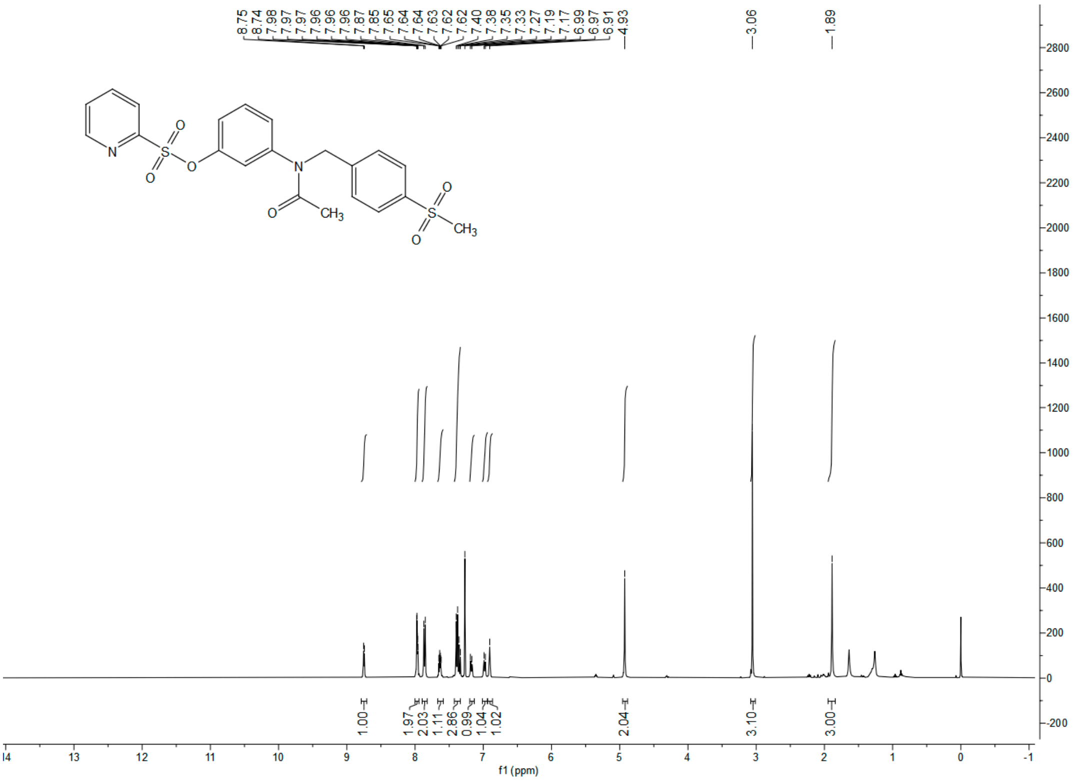

^{13}C NMR spectra of compound **1n**

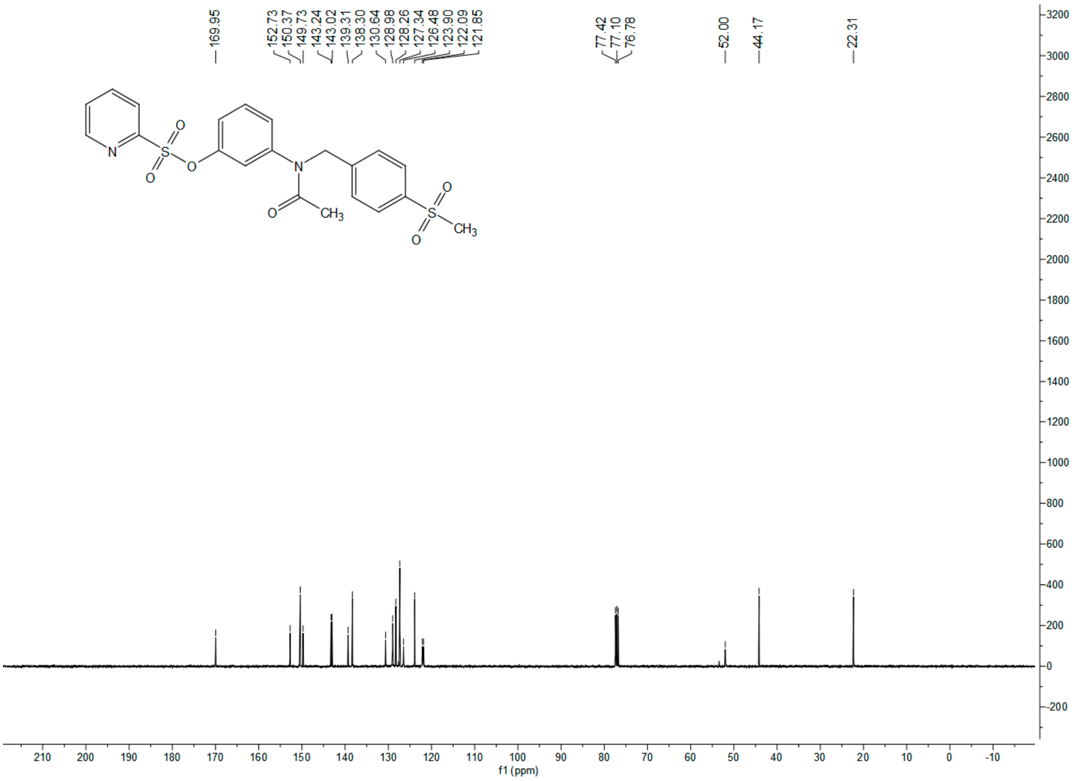

^{1}H NMR spectra of compound **1o**

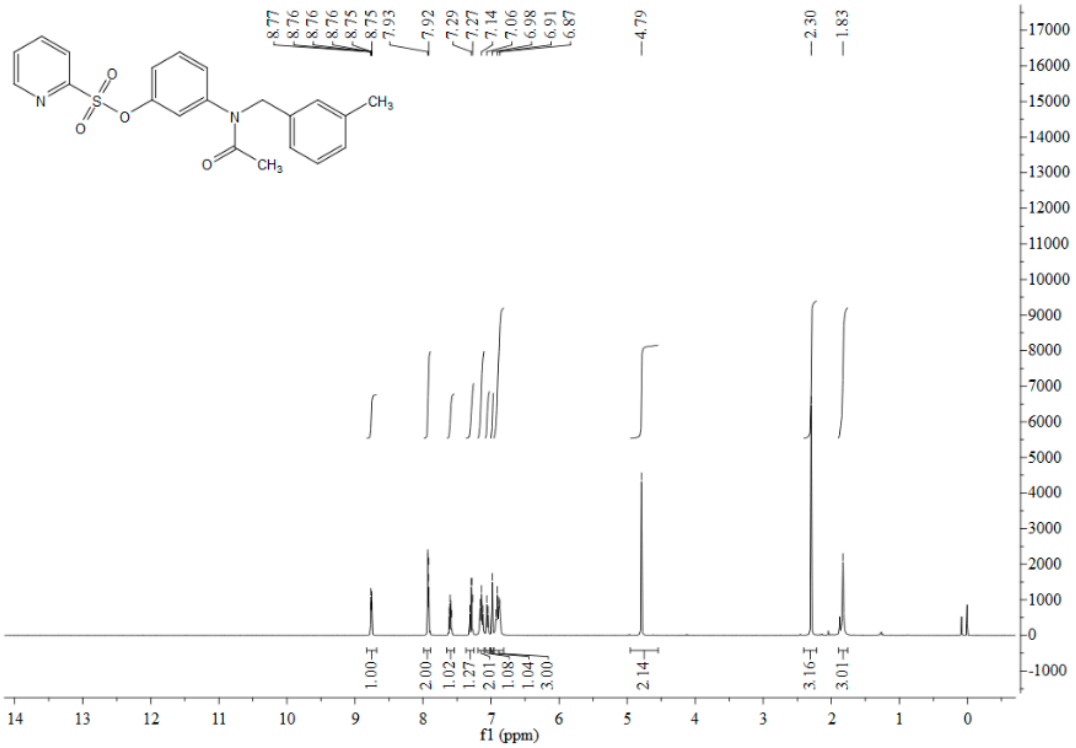

^{13}C NMR spectra of compound **1o**

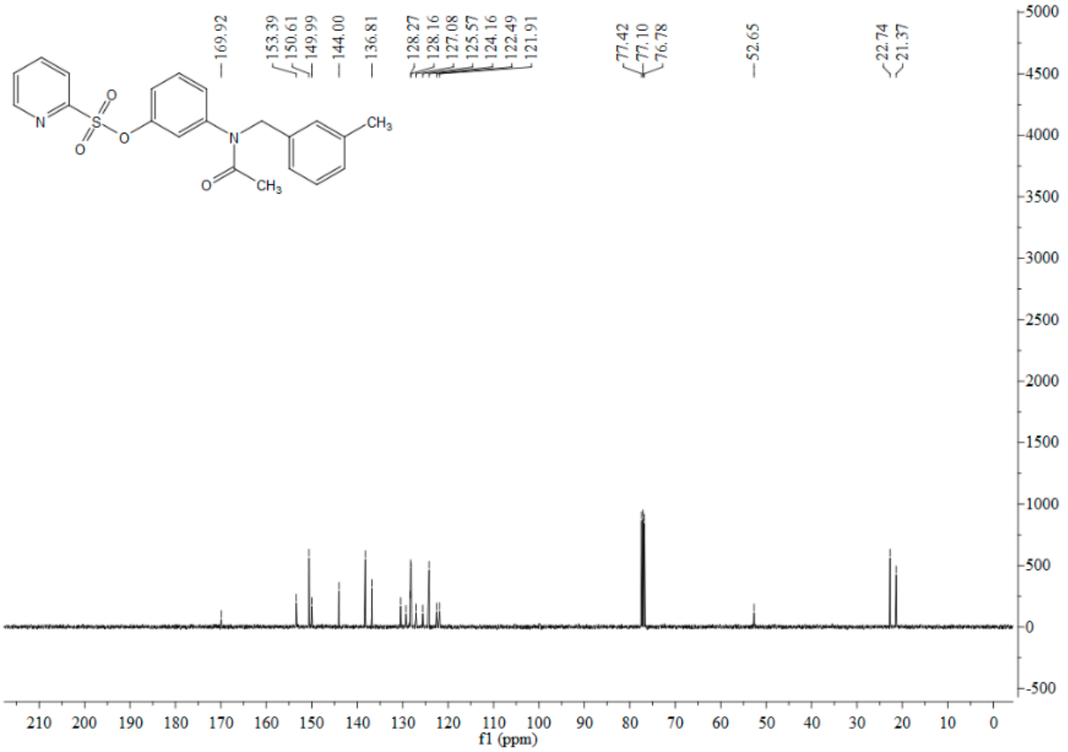

^{1}H NMR spectra of compound **1p**

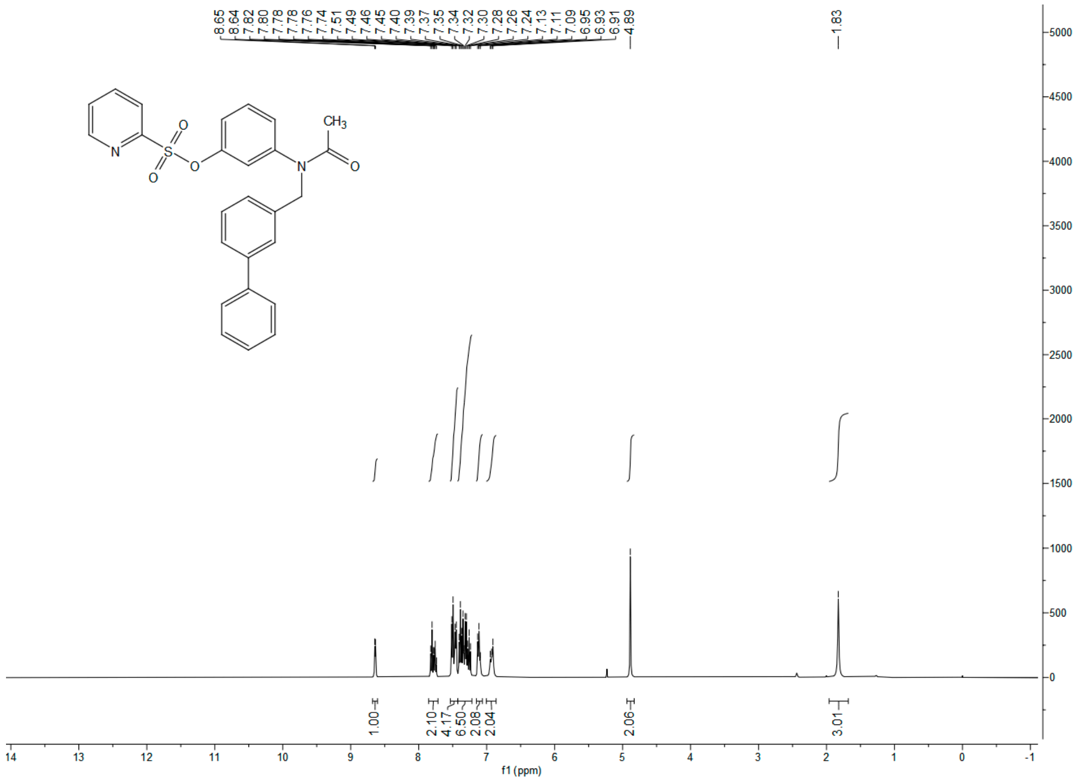

^{13}C NMR spectra of compound **1p**

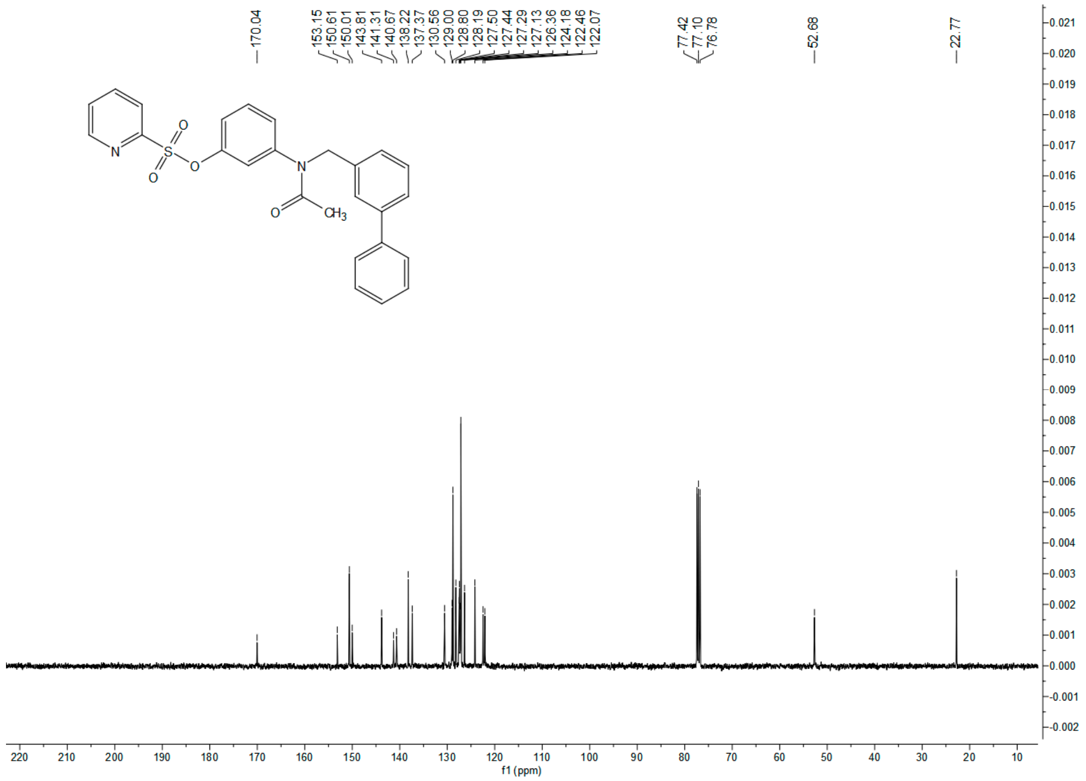

^{1}H NMR spectra of compound **1q**

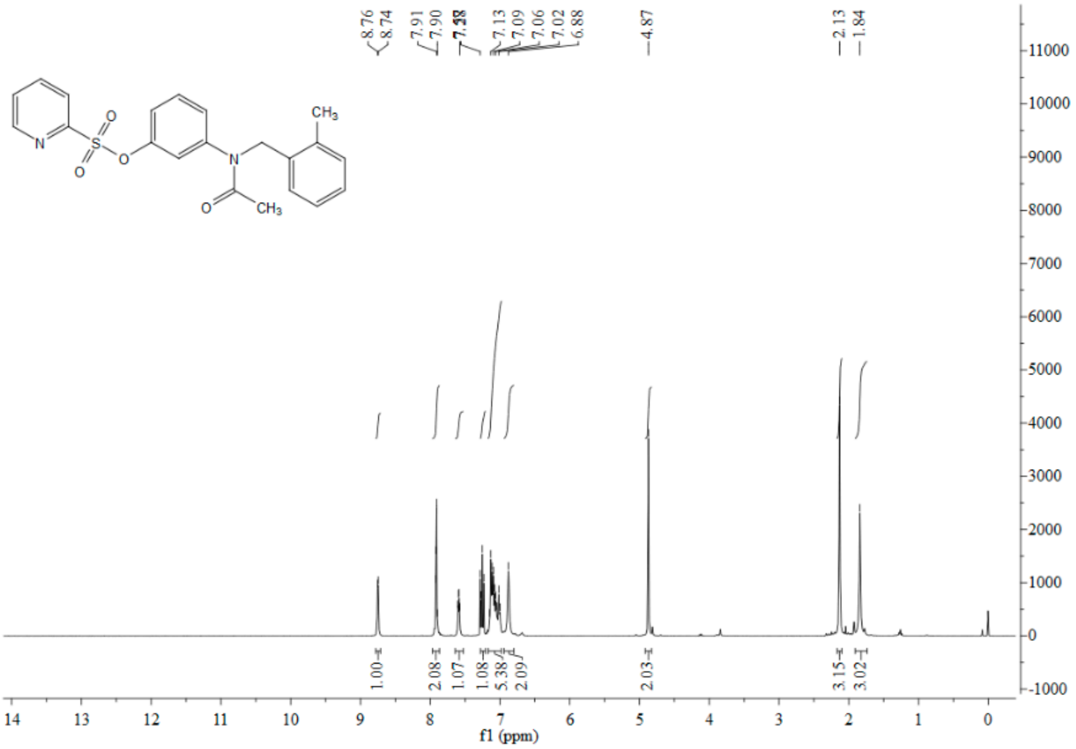

^{13}C NMR spectra of compound **1q**

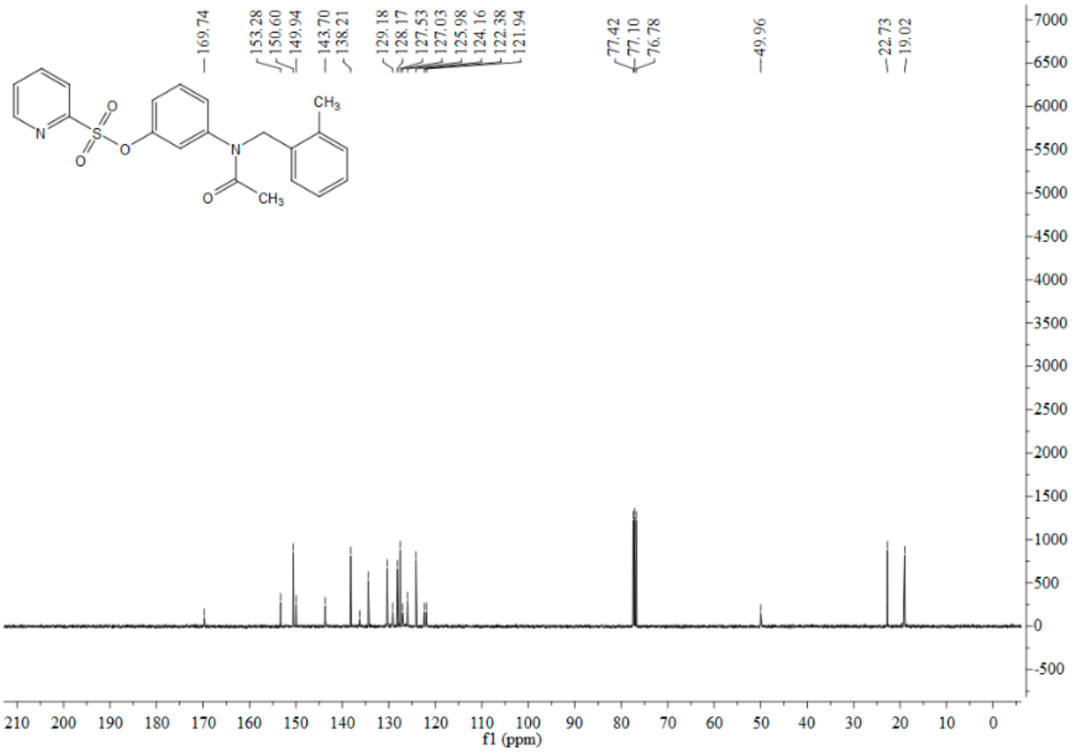

^{1}H NMR spectra of compound **1r**

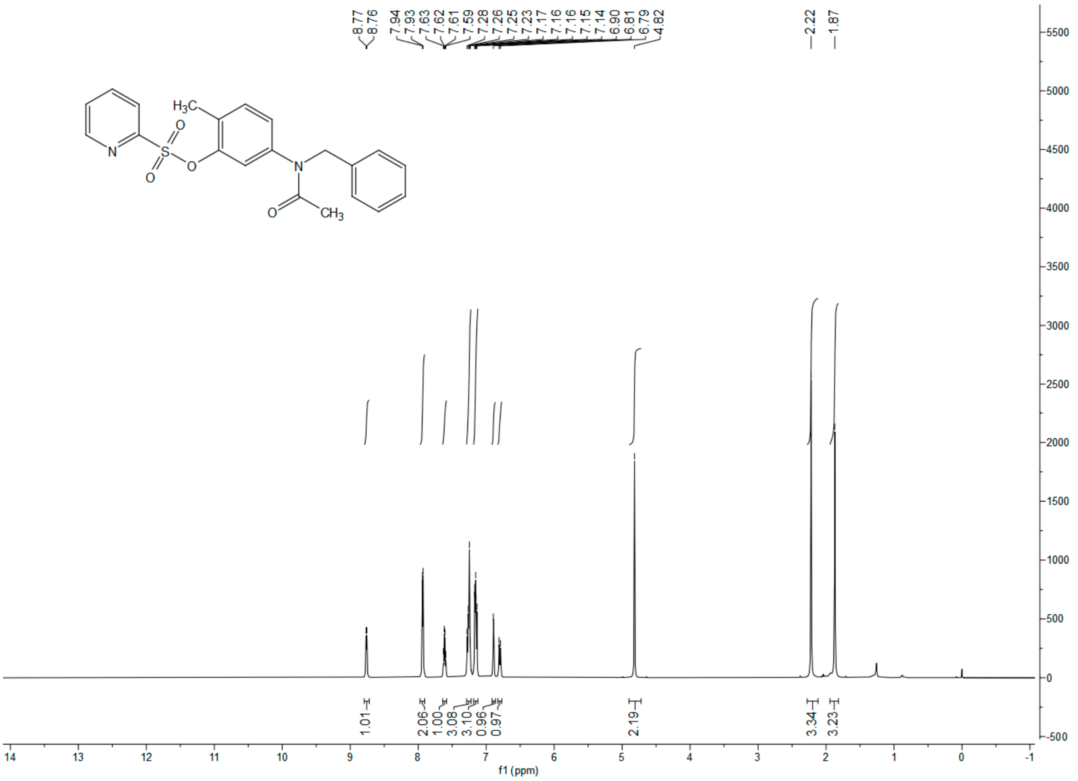

^{13}C NMR spectra of compound **1r**

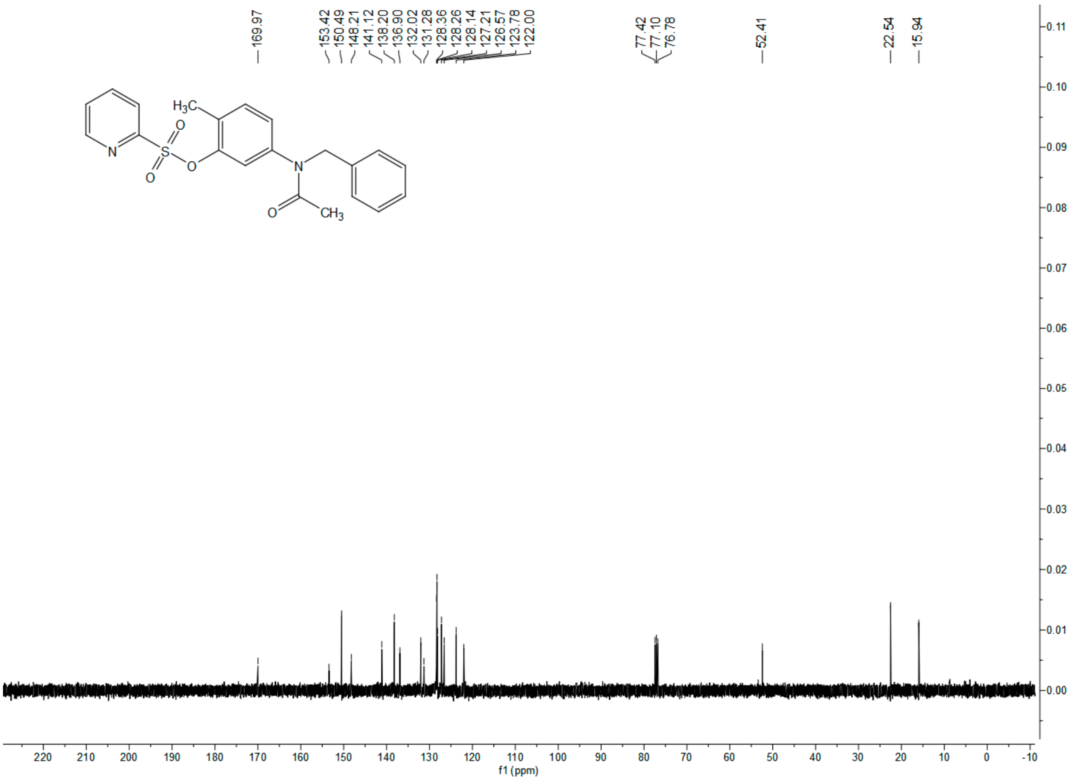

^{1}H NMR spectra of compound **1s**

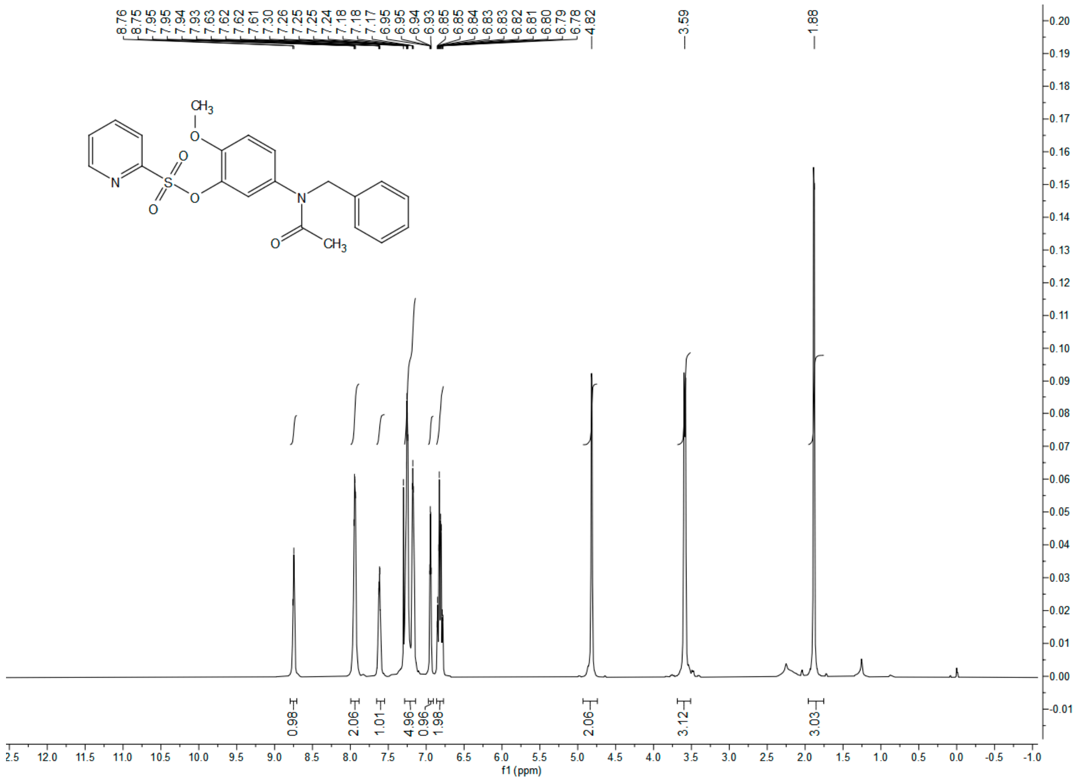

^{13}C NMR spectra of compound **1s**

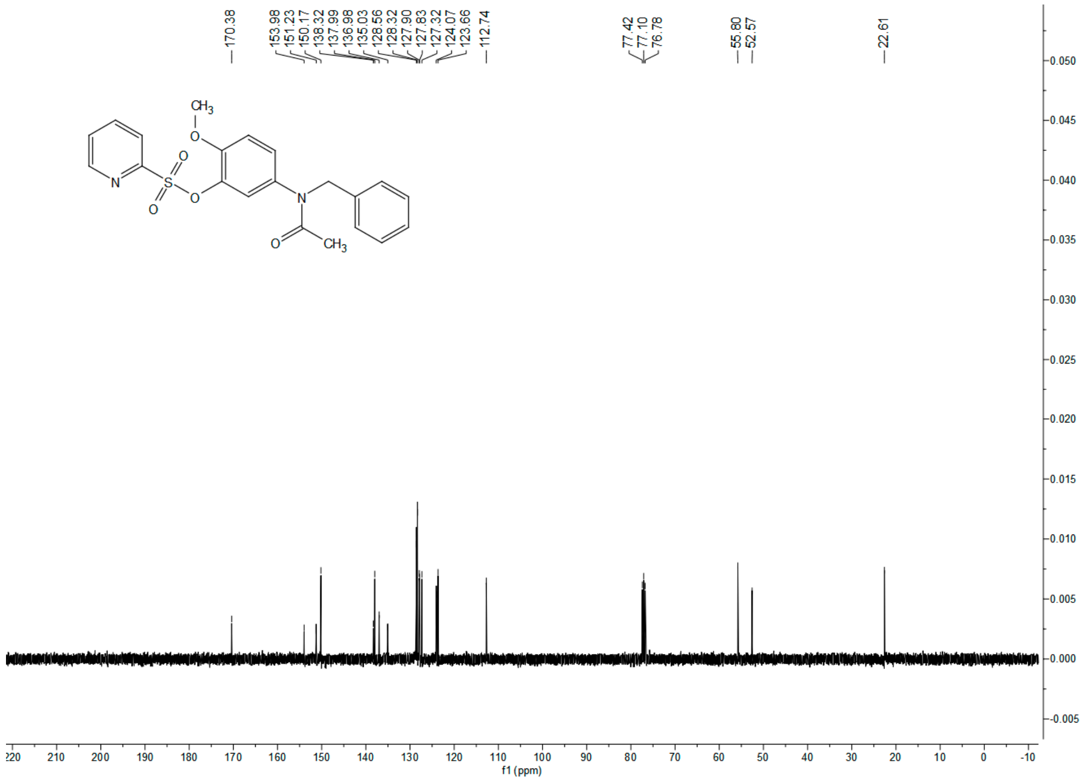

^{1}H NMR spectra of compound **1t**

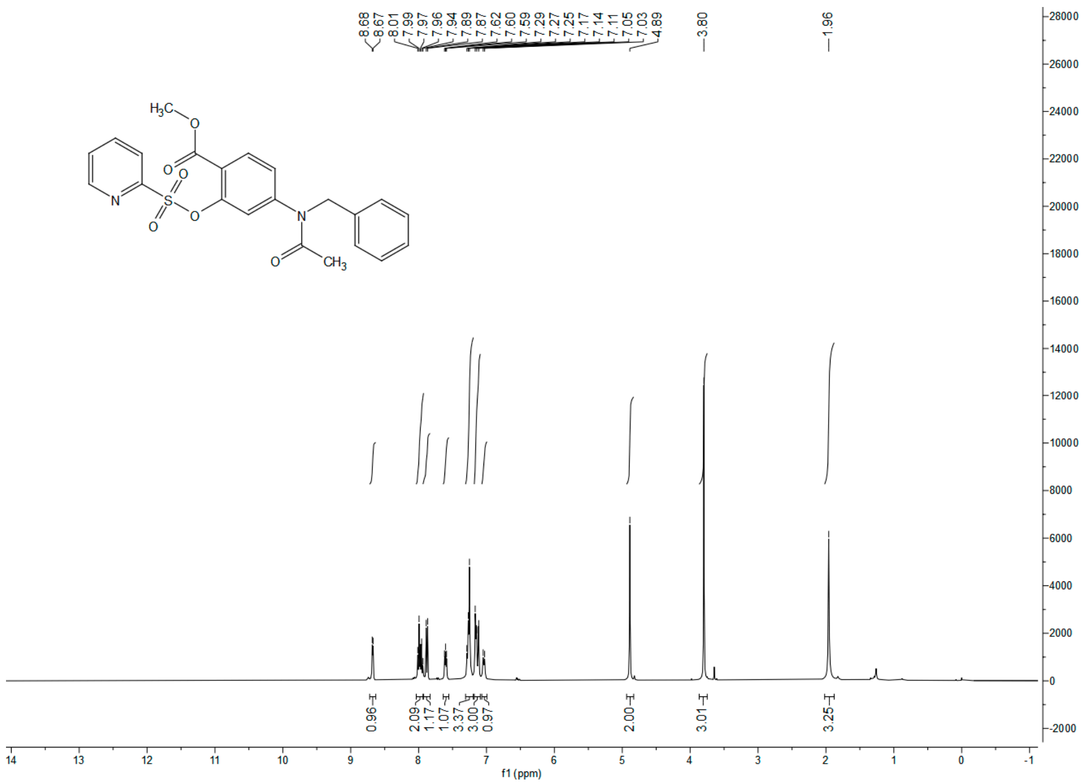

^{13}C NMR spectra of compound **1t**

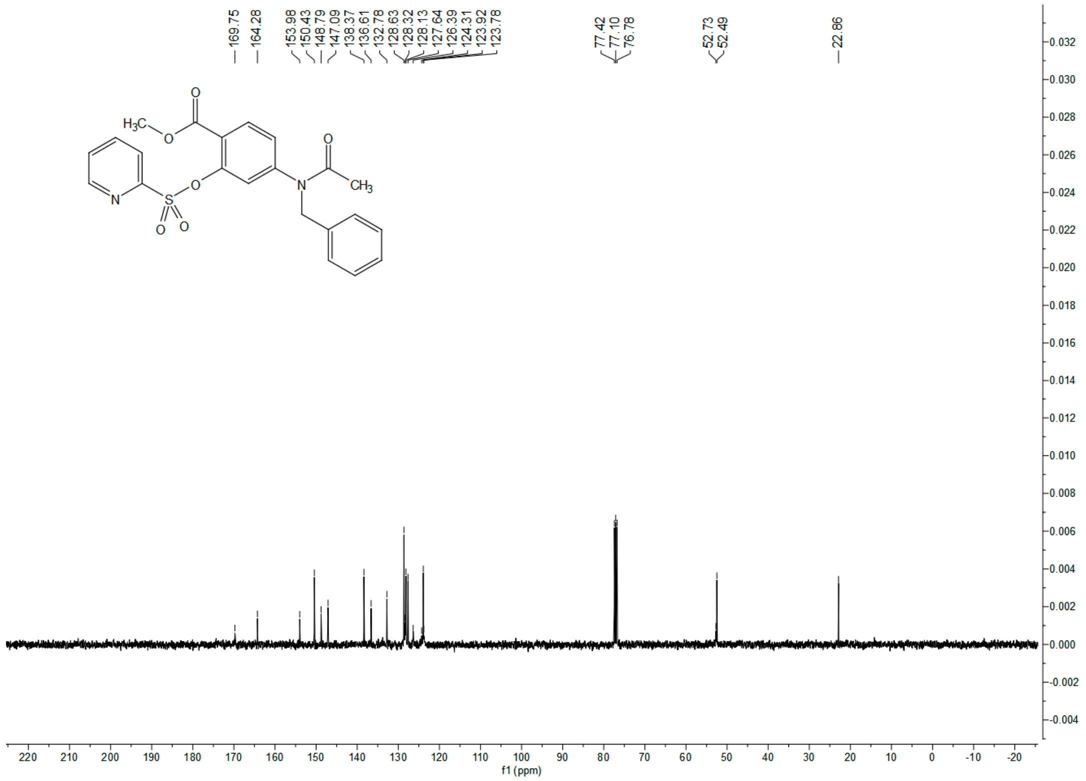

5. Spectral copies of products ^{1}H NMR and ^{13}C NMR

^{1}H NMR spectra of compound **2a**

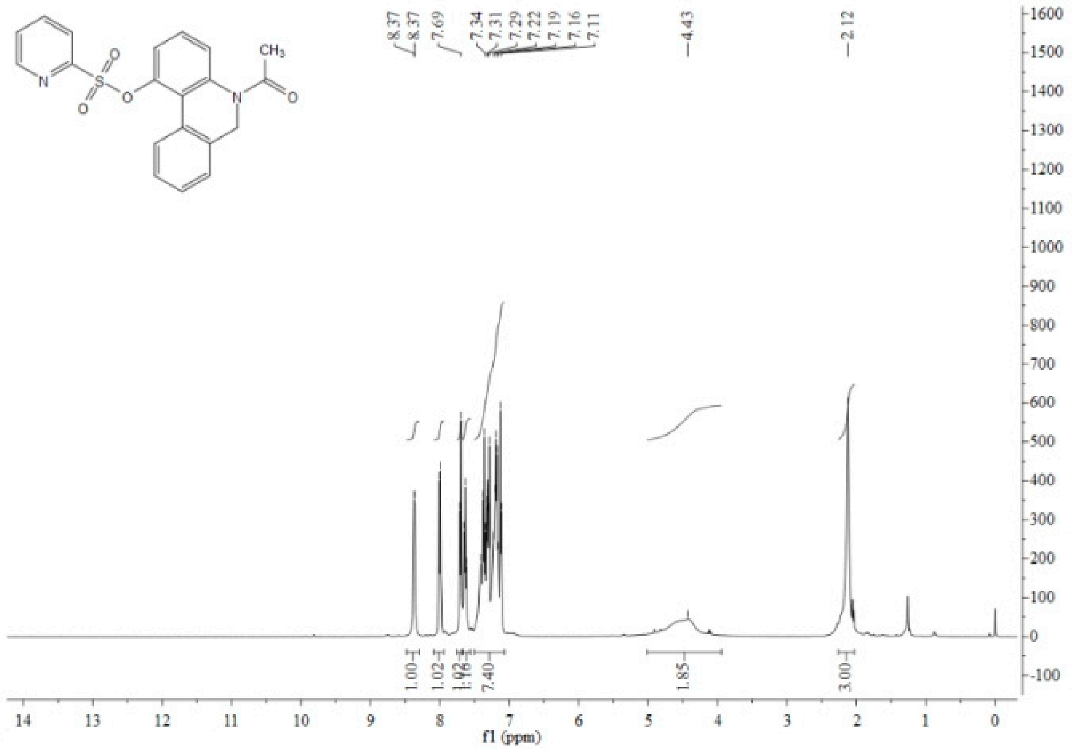

^{13}C NMR spectra of compound **2a**

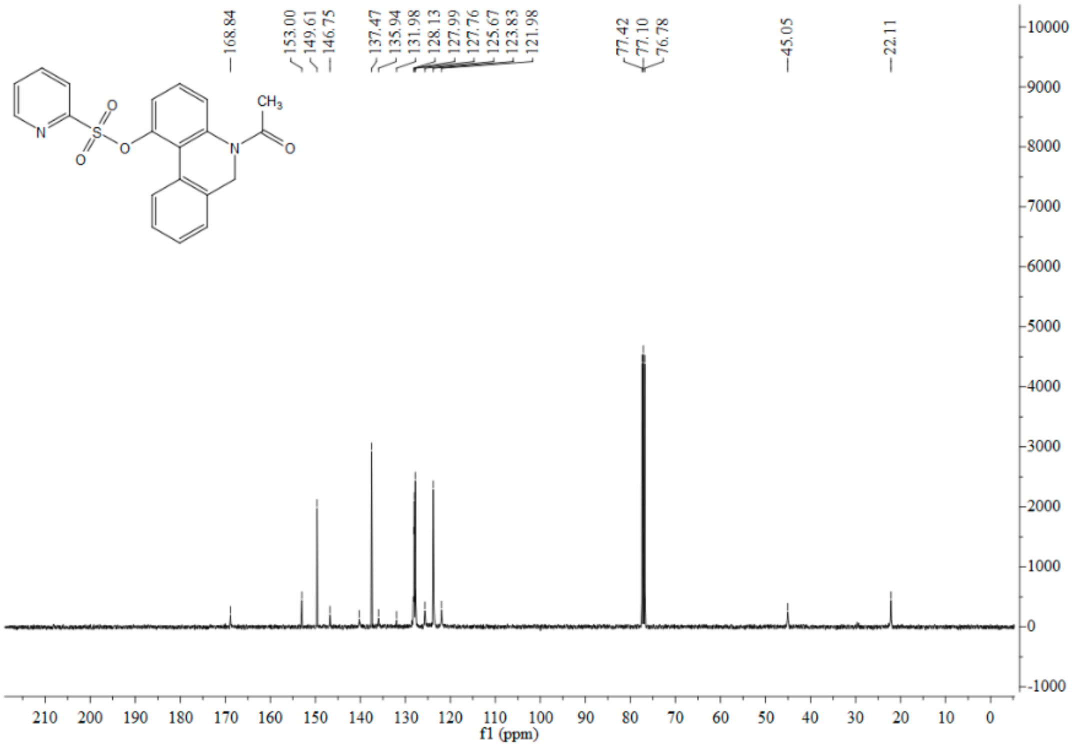

^{1}H NMR spectra of compound **2b**

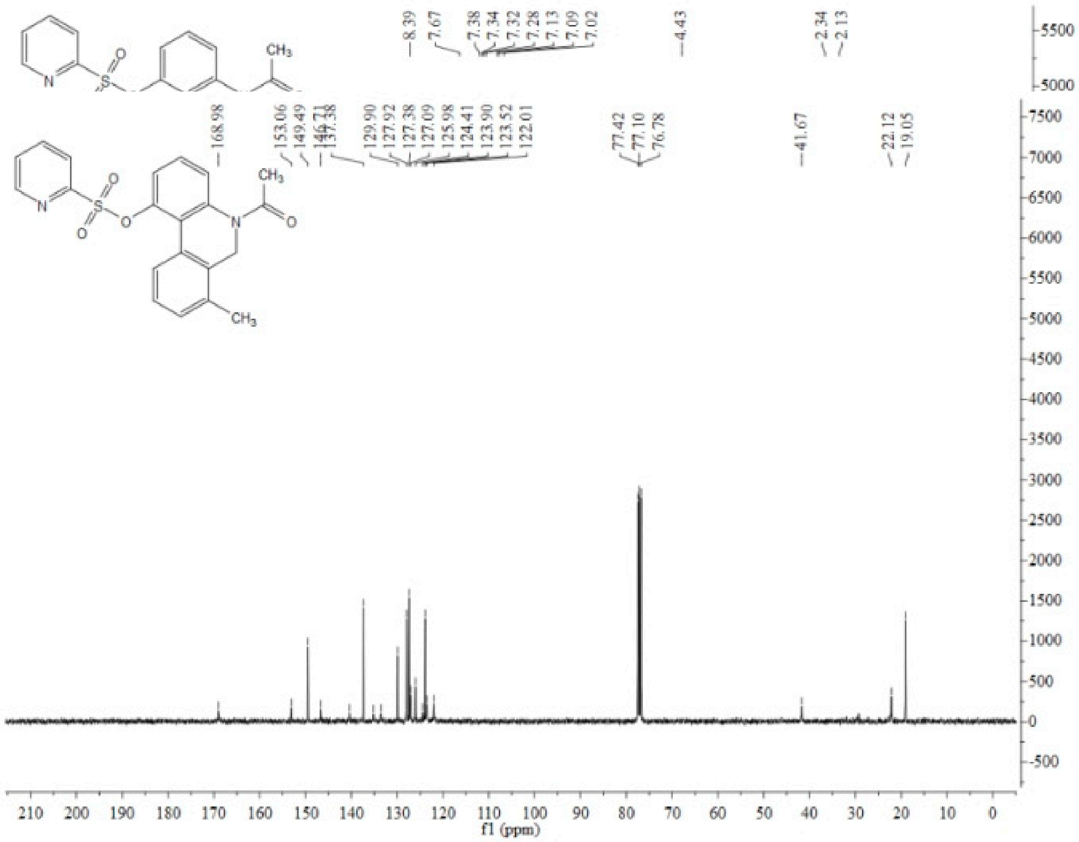

^{13}C NMR spectra of compound **2b**

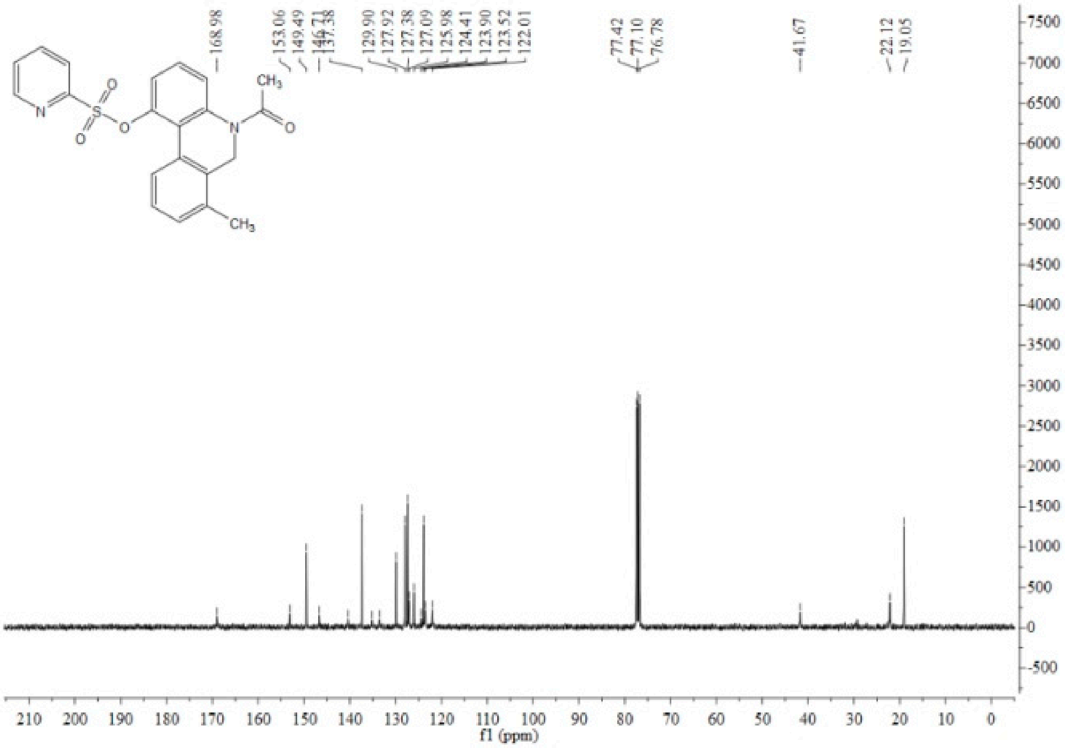

^{1}H NMR spectra of compound **2c**

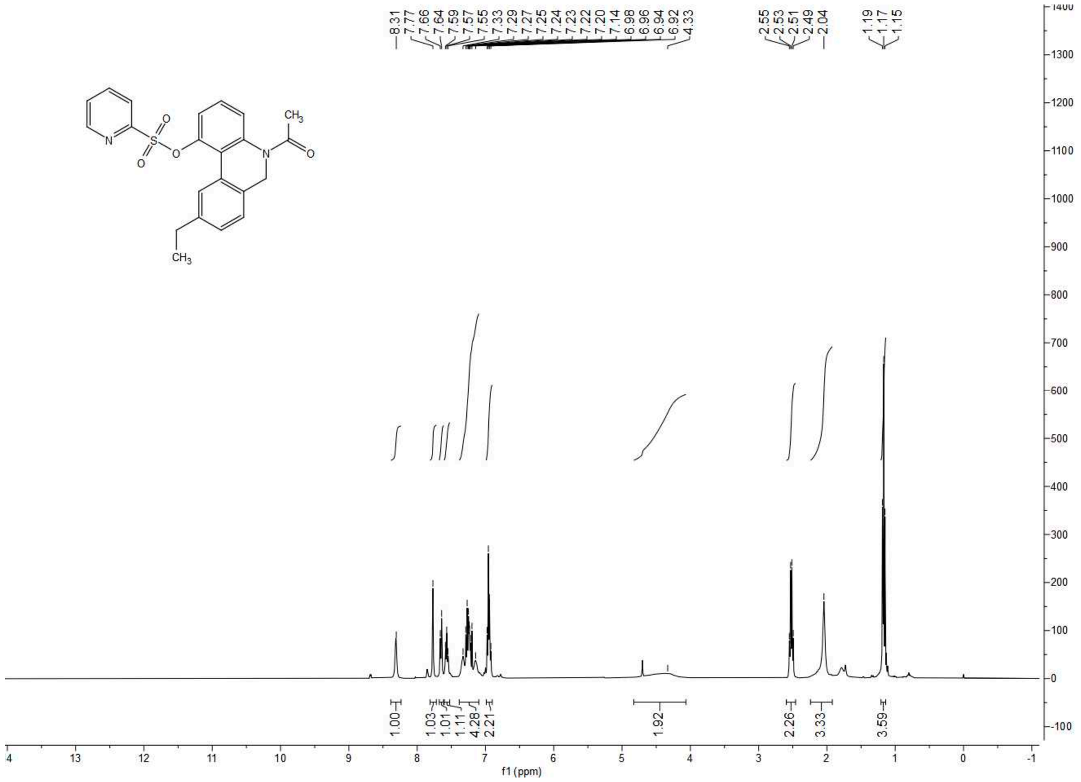

^{13}C NMR spectra of compound **2c**

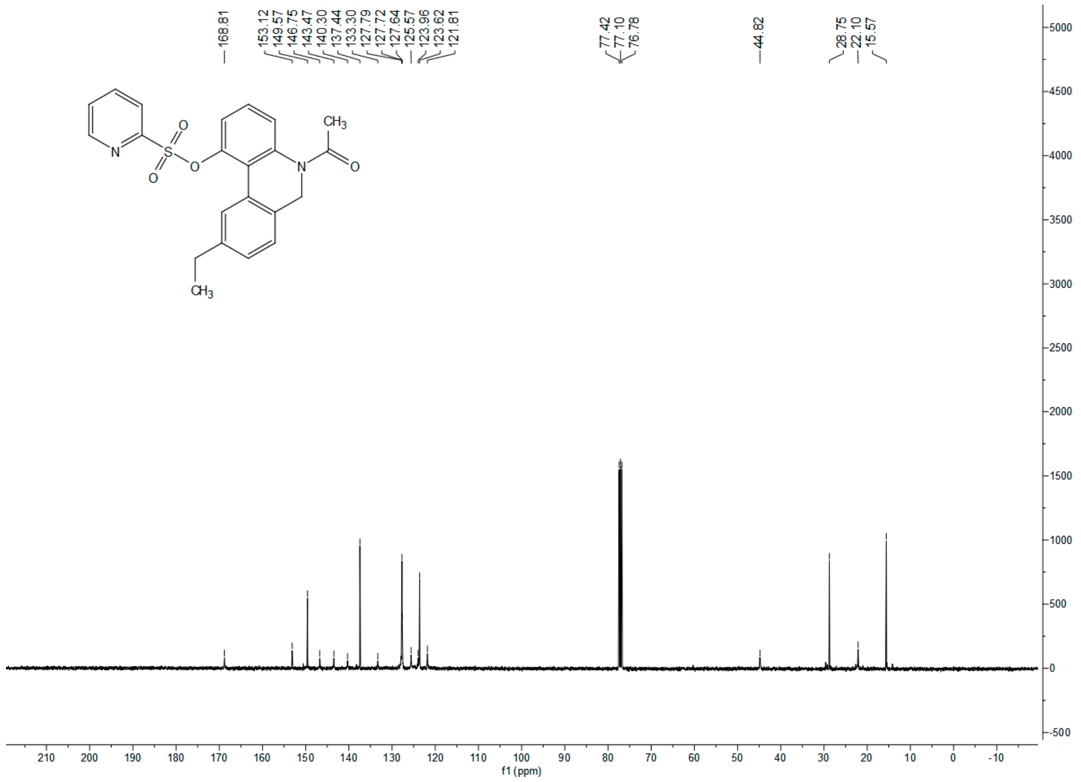

^{1}H NMR spectra of compound **2d**

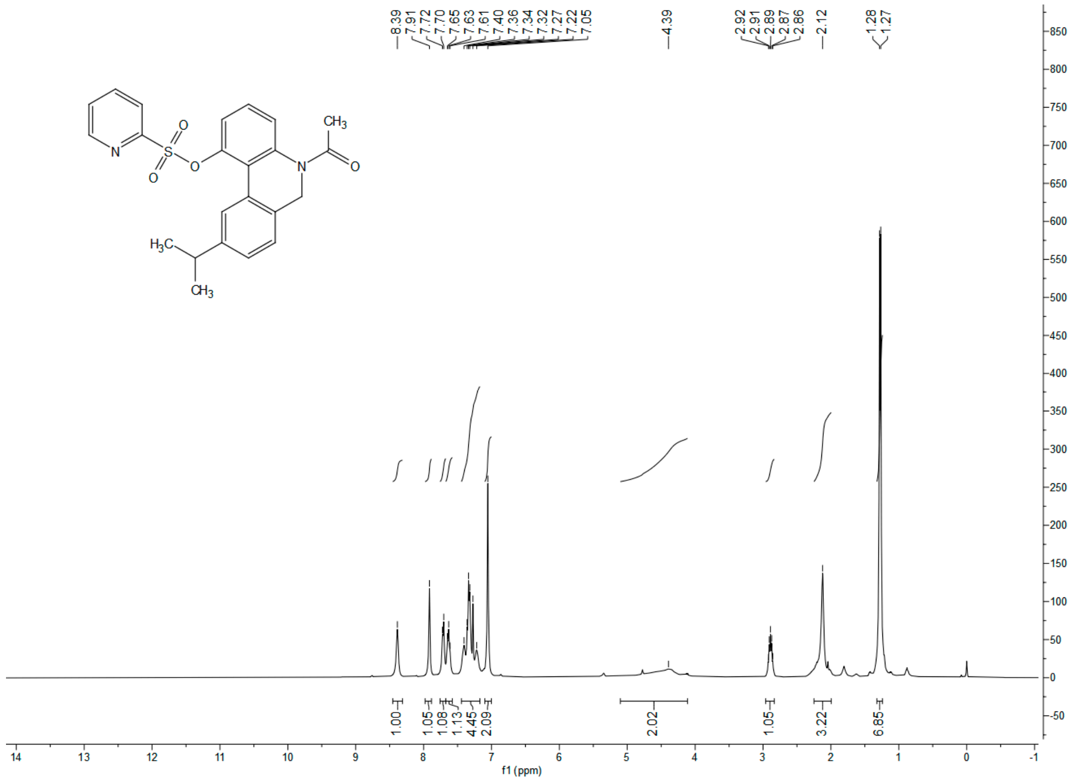

^{13}C NMR spectra of compound **2d**

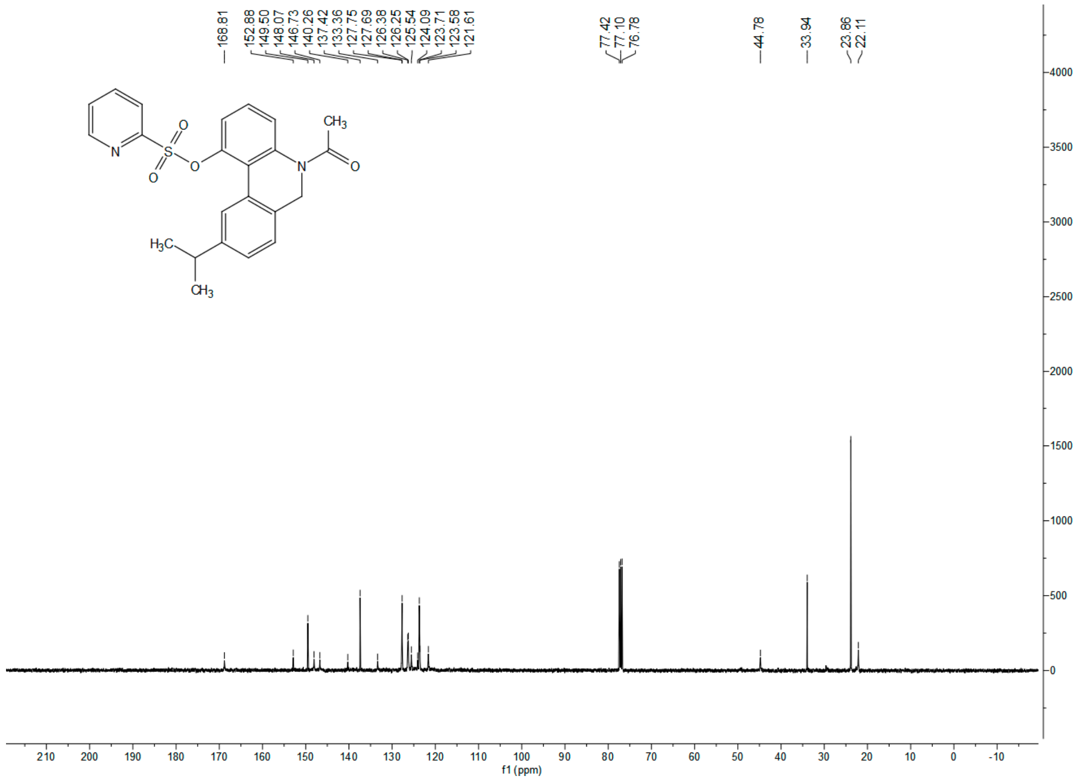

^{1}H NMR spectra of compound **2e**

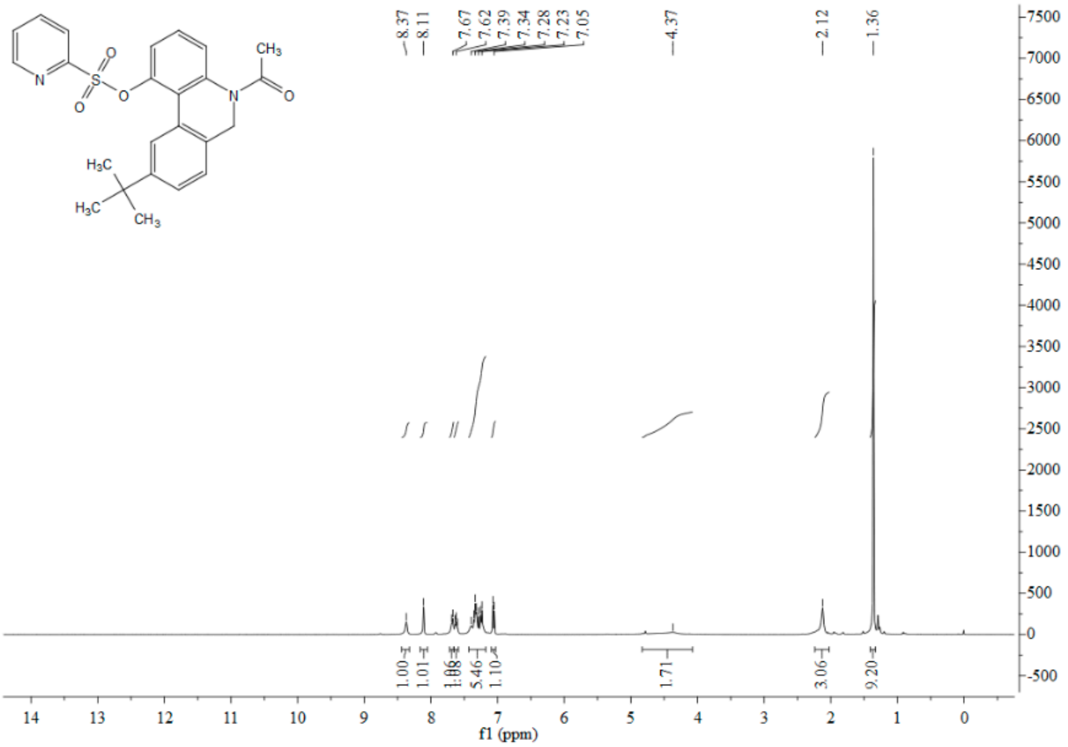

^{13}C NMR spectra of compound **2e**

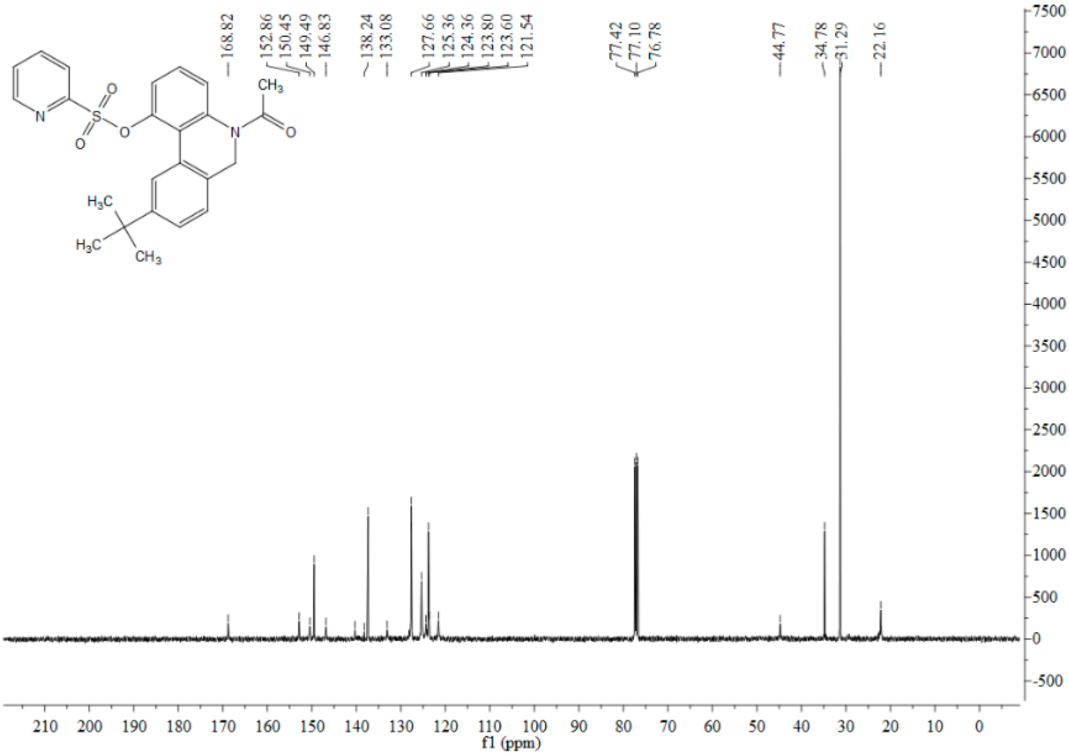

^{1}H NMR spectra of compound **2f**

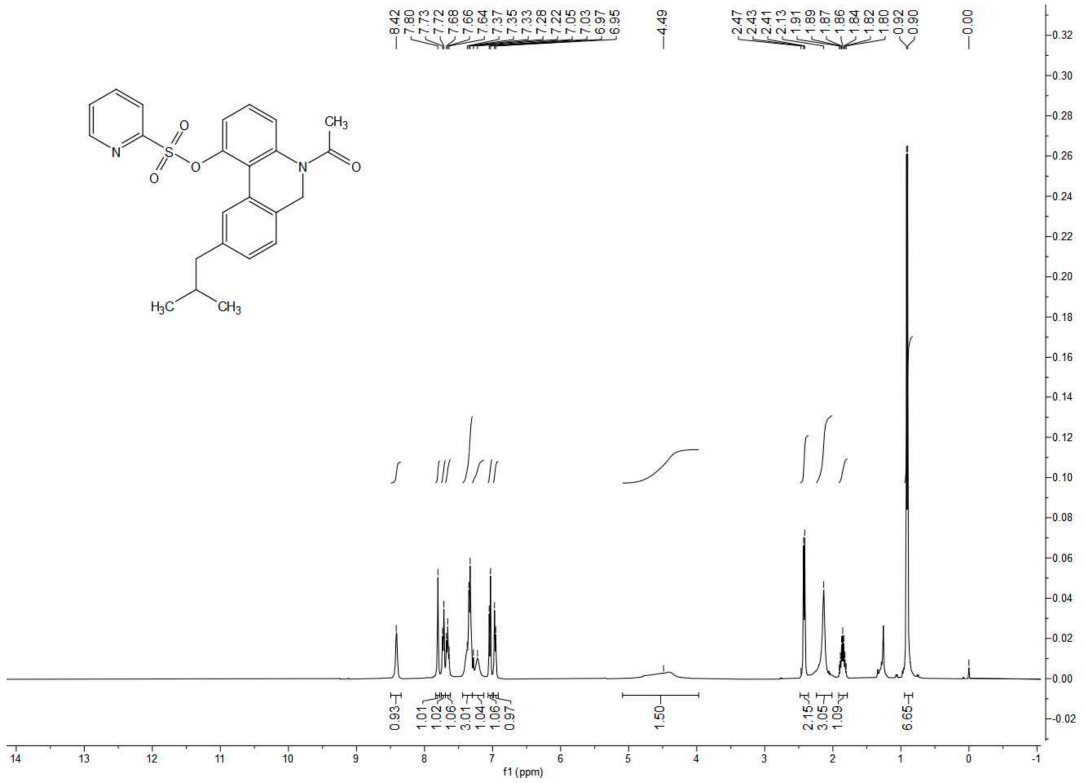

^{13}C NMR spectra of compound **2f**

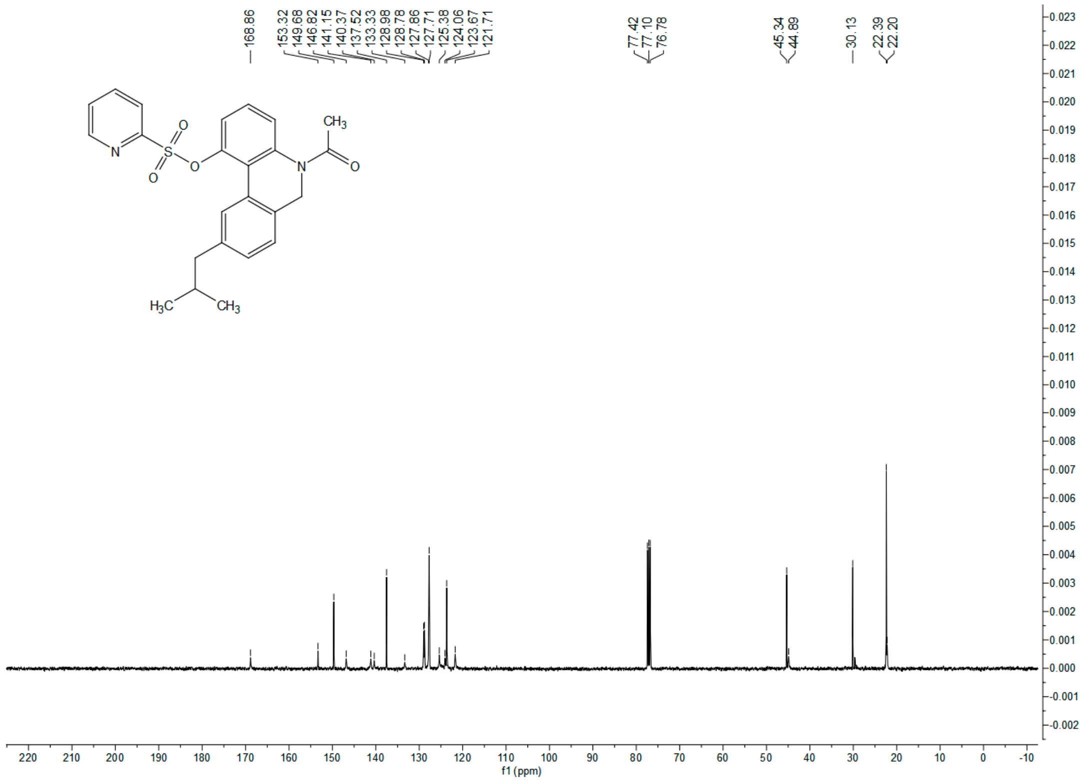

^{1}H NMR spectra of compound **2g**

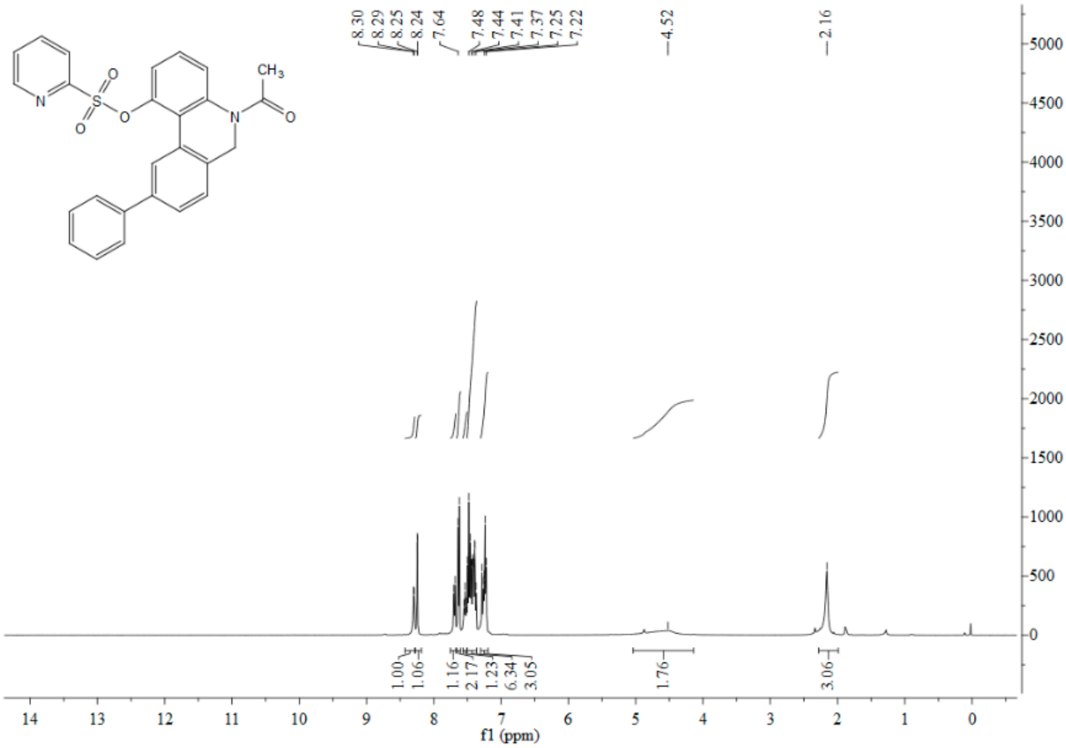

^{13}C NMR spectra of compound **2g**

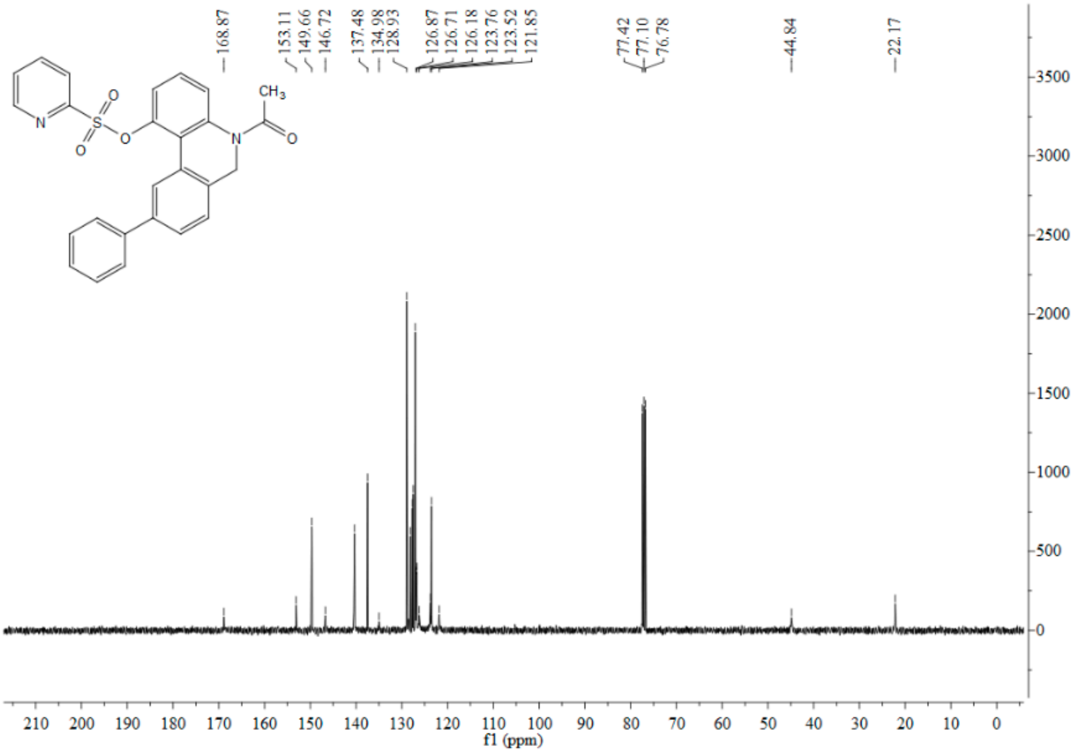

^{1}H NMR spectra of compound **2h**

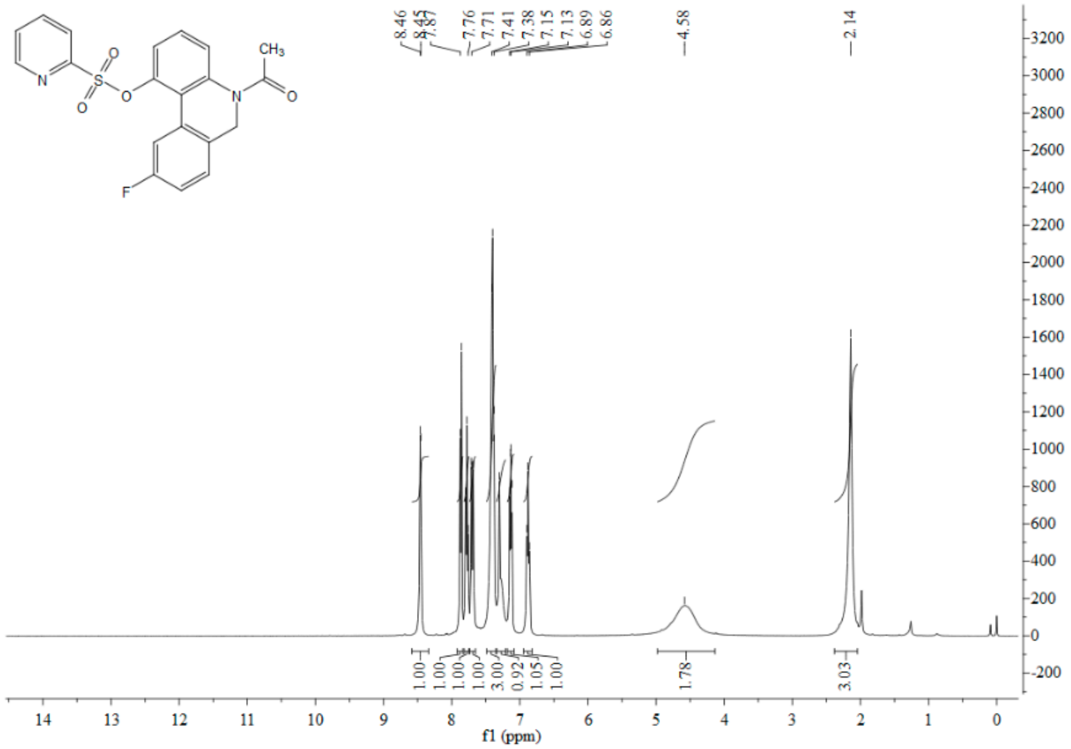

^{13}C NMR spectra of compound **2h**

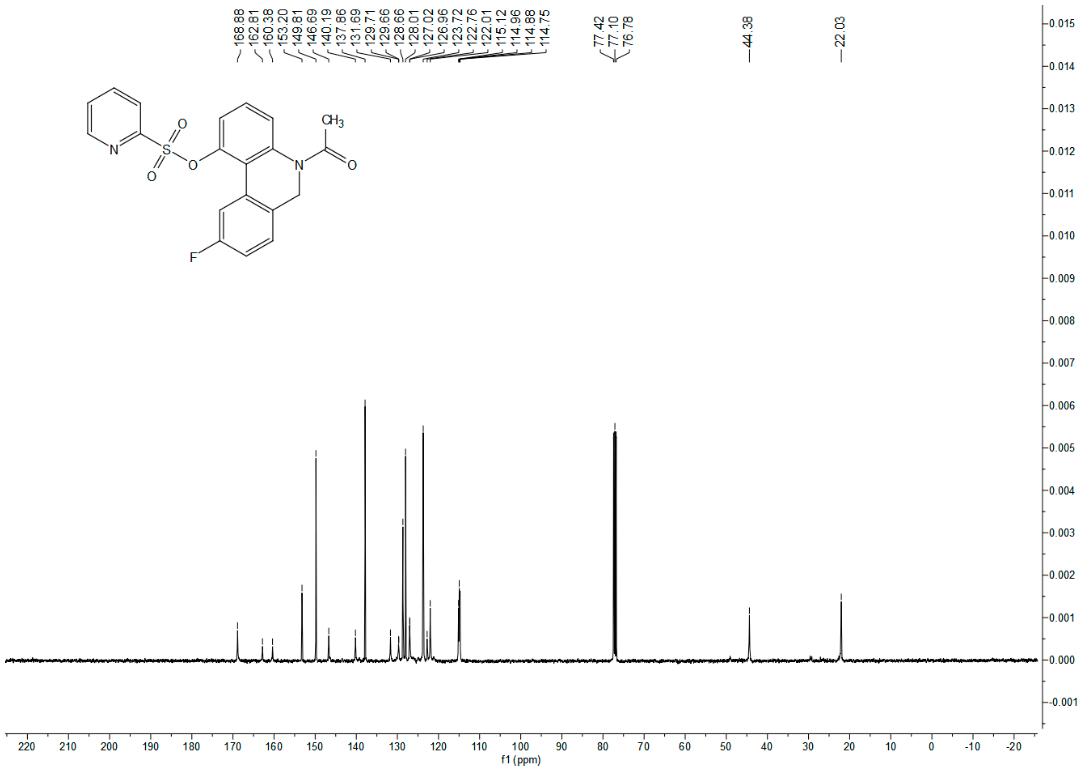

^{1}H NMR spectra of compound **2i**

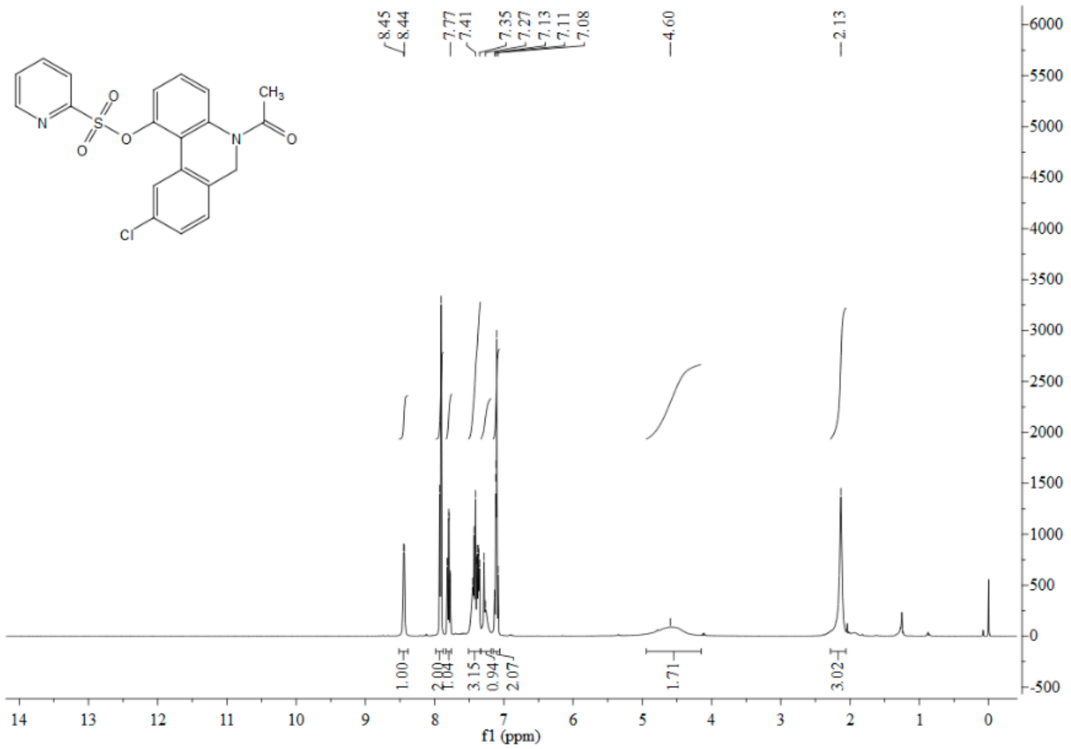

^{13}C NMR spectra of compound **2i**

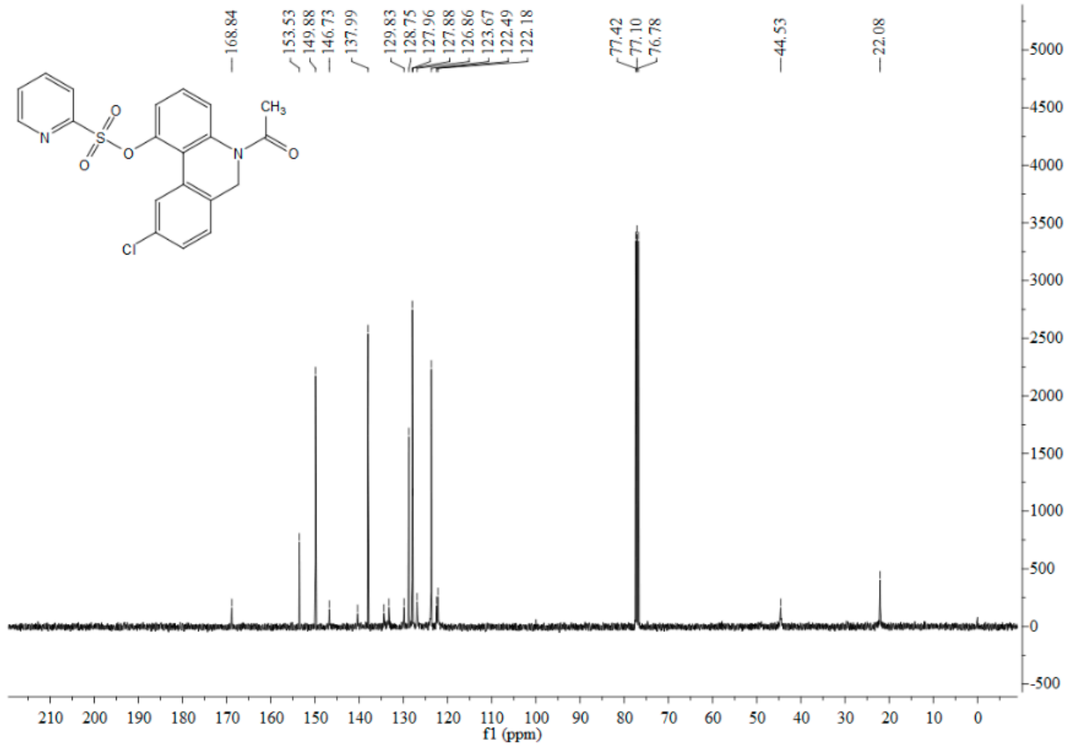

^{1}H NMR spectra of compound **2j**

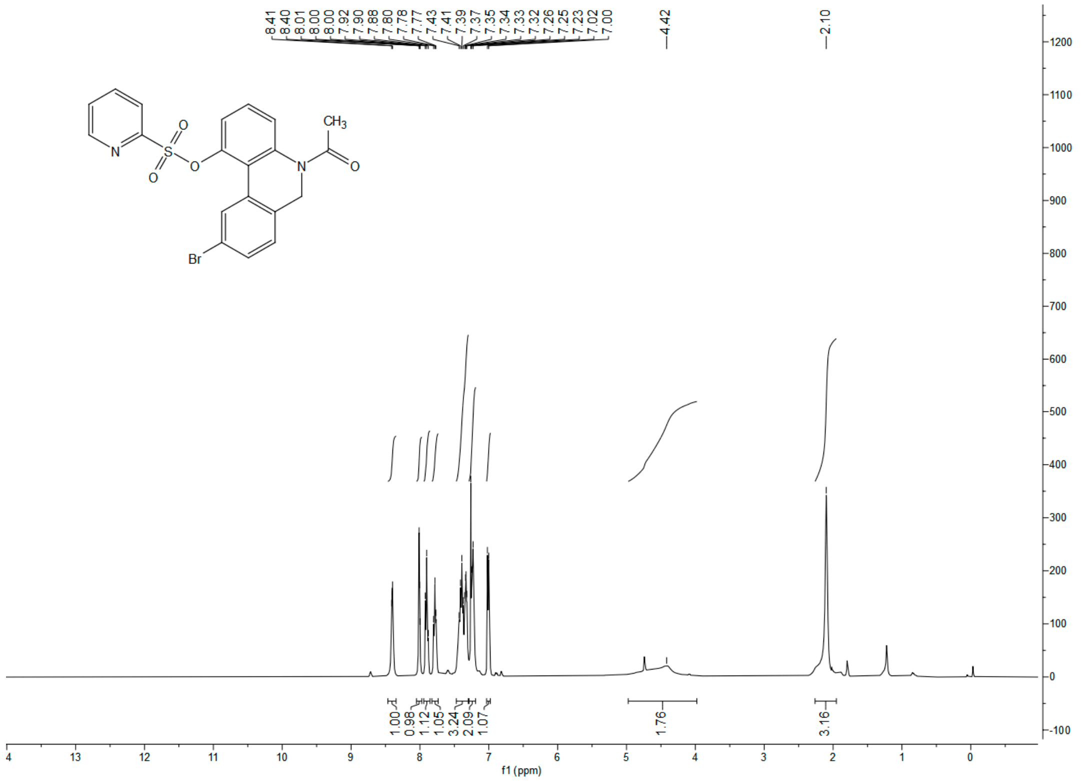

^{13}C NMR spectra of compound **2j**

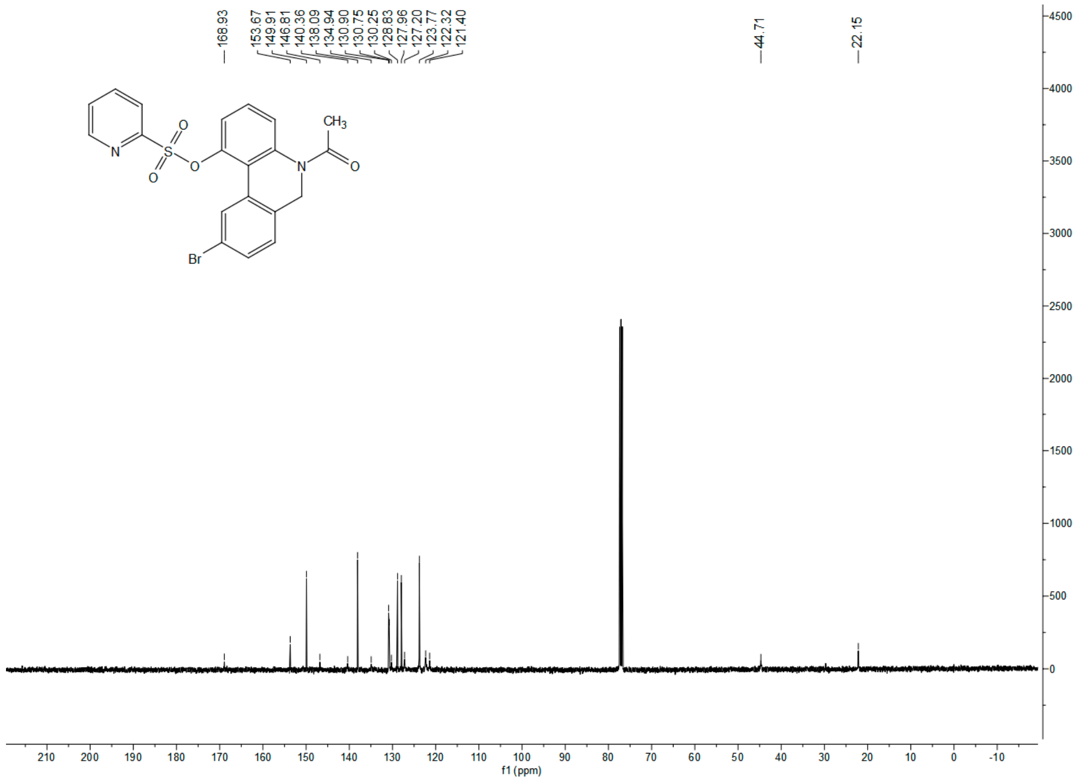

^{1}H NMR spectra of compound **2k**

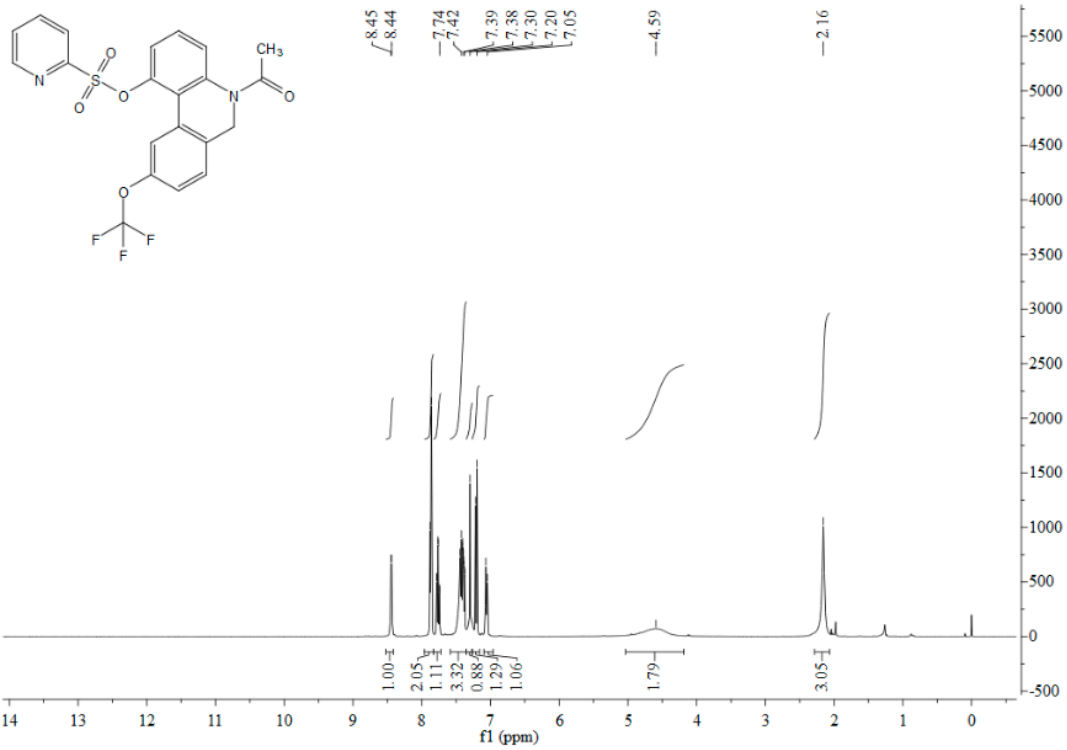

^{13}C NMR spectra of compound **2k**

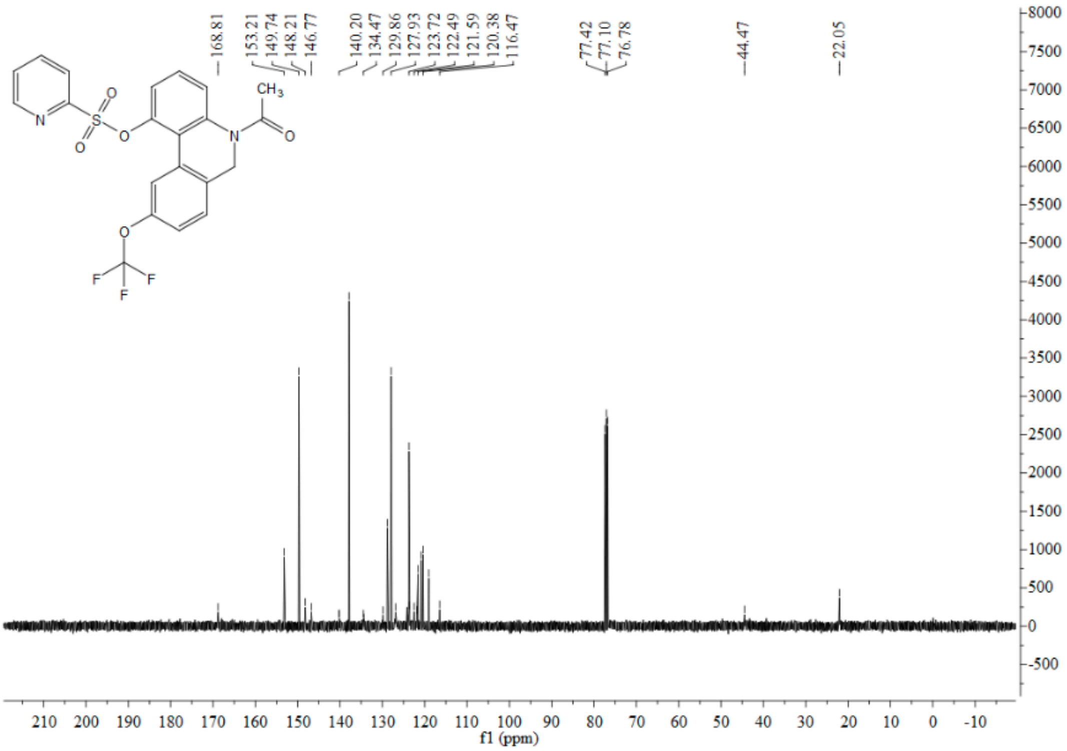

^{1}H NMR spectra of compound **2l**

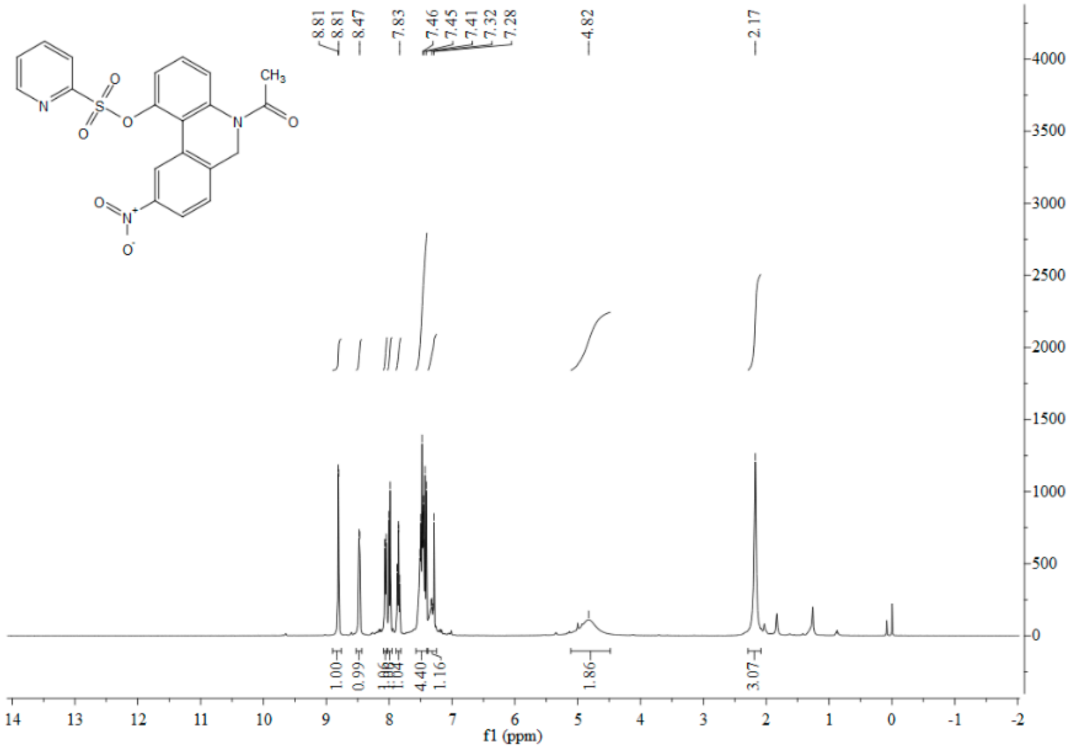

^{13}C NMR spectra of compound **2l**

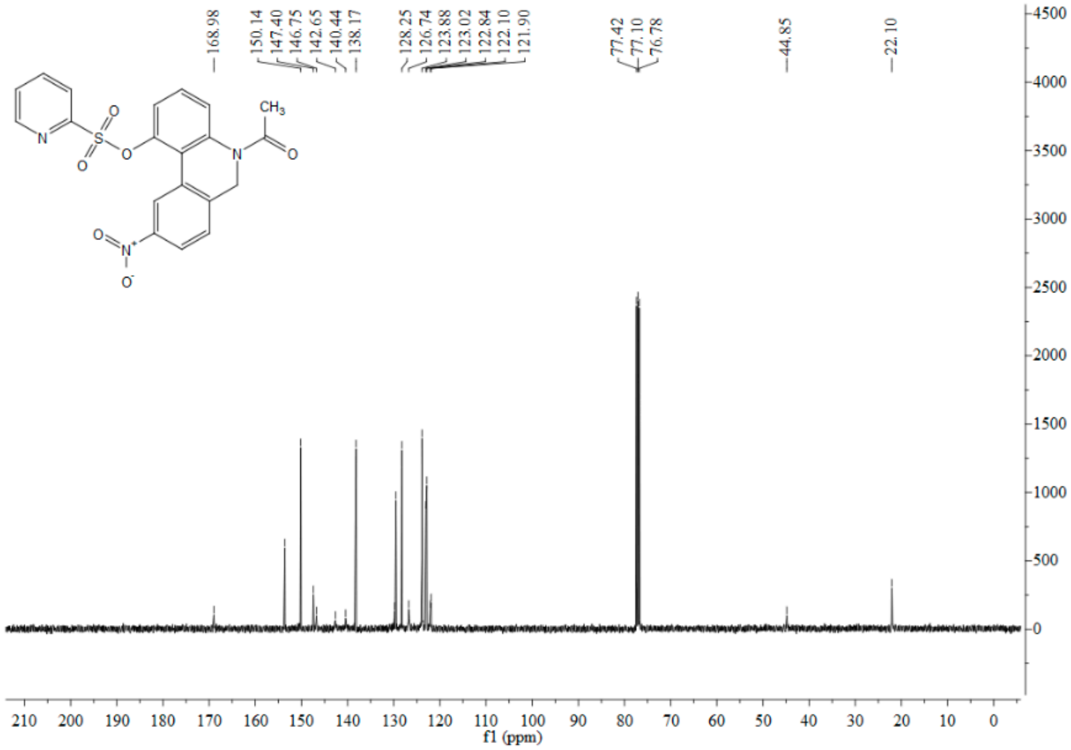

^{1}H NMR spectra of compound **2m**

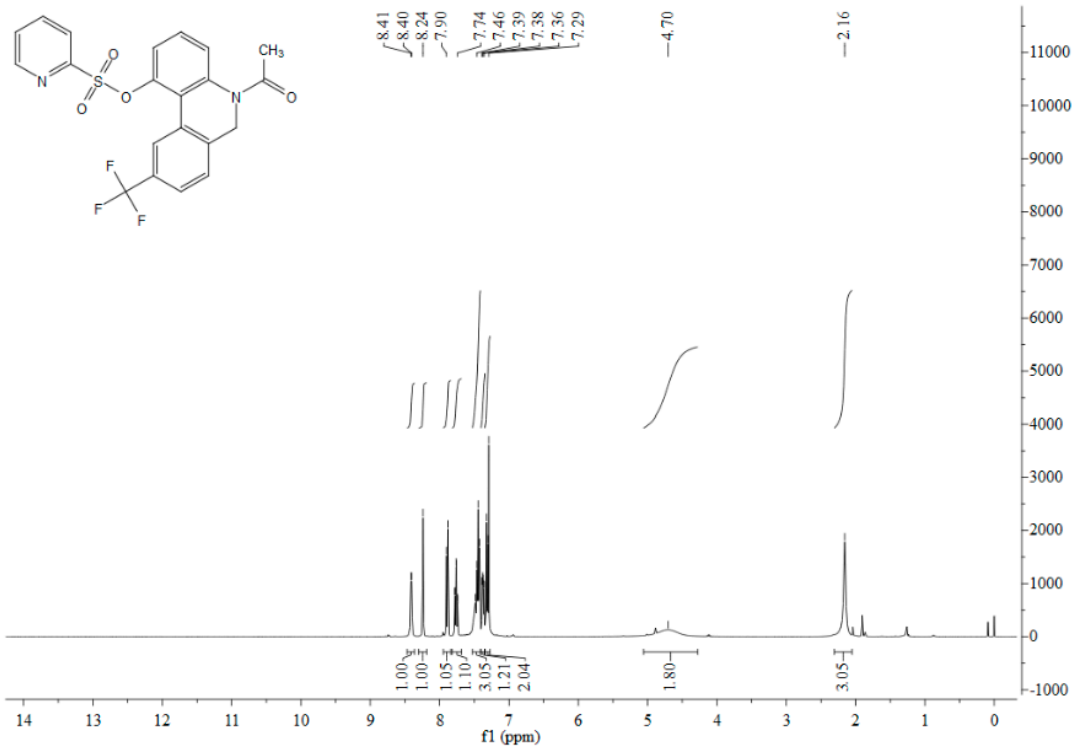

^{13}C NMR spectra of compound **2m**

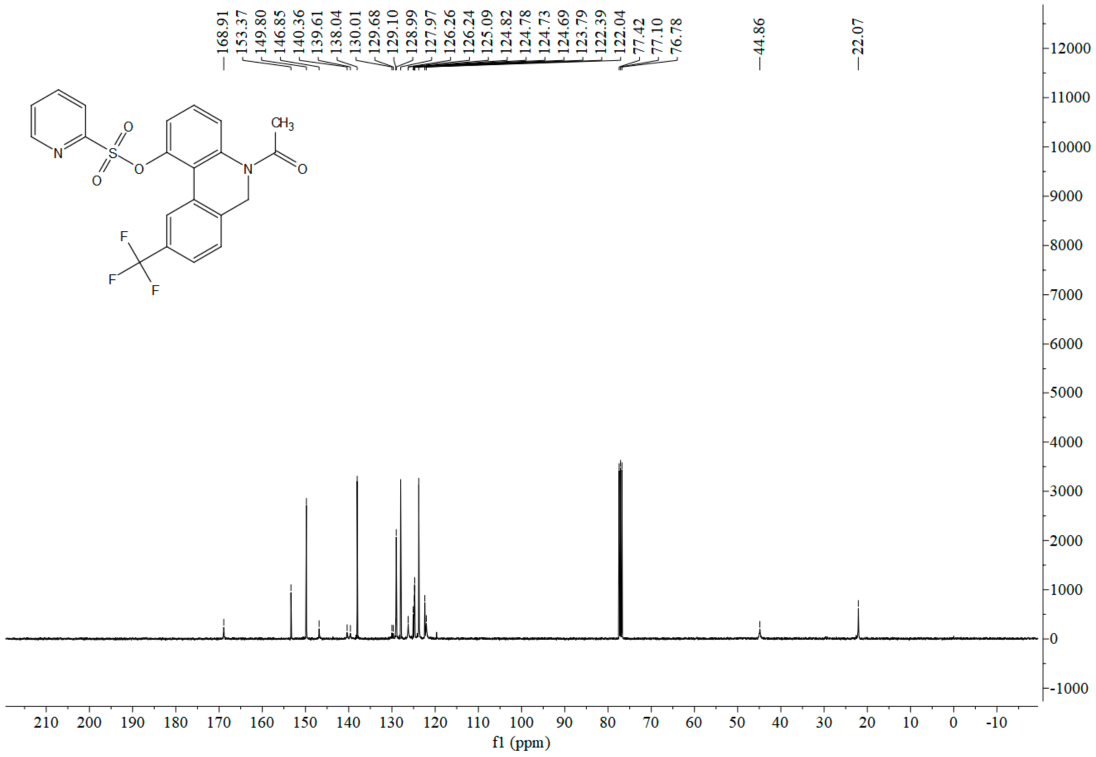

^{1}H NMR spectra of compound **2n**

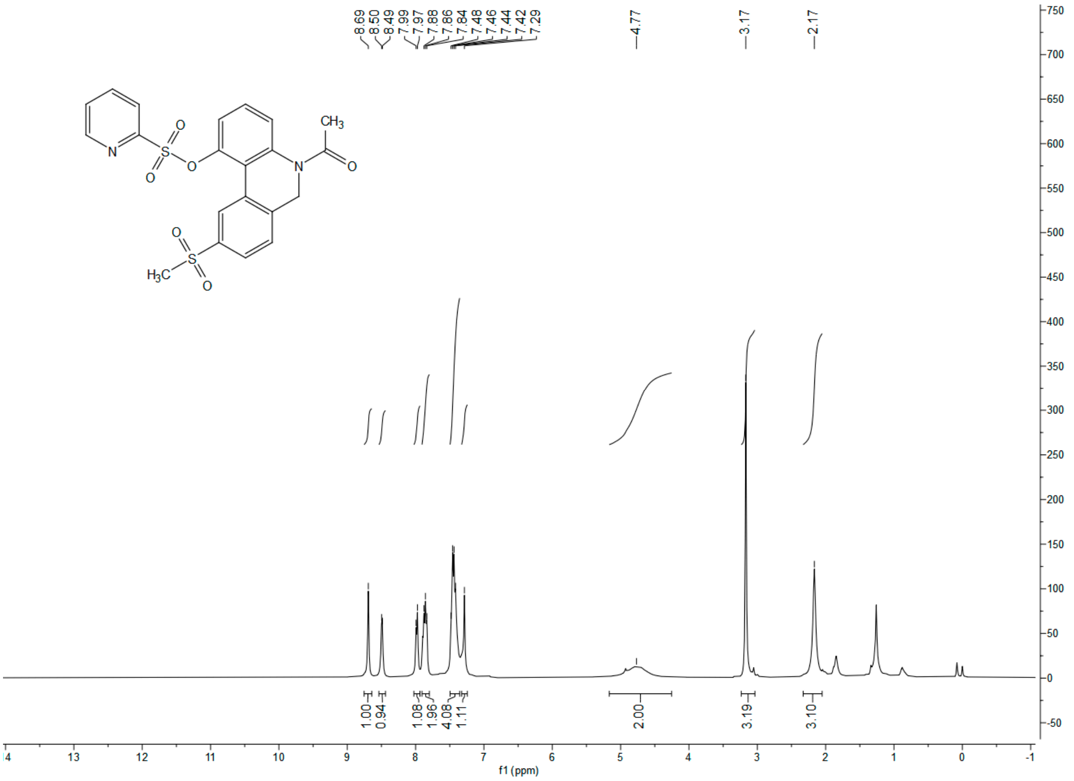

^{13}C NMR spectra of compound **2n**

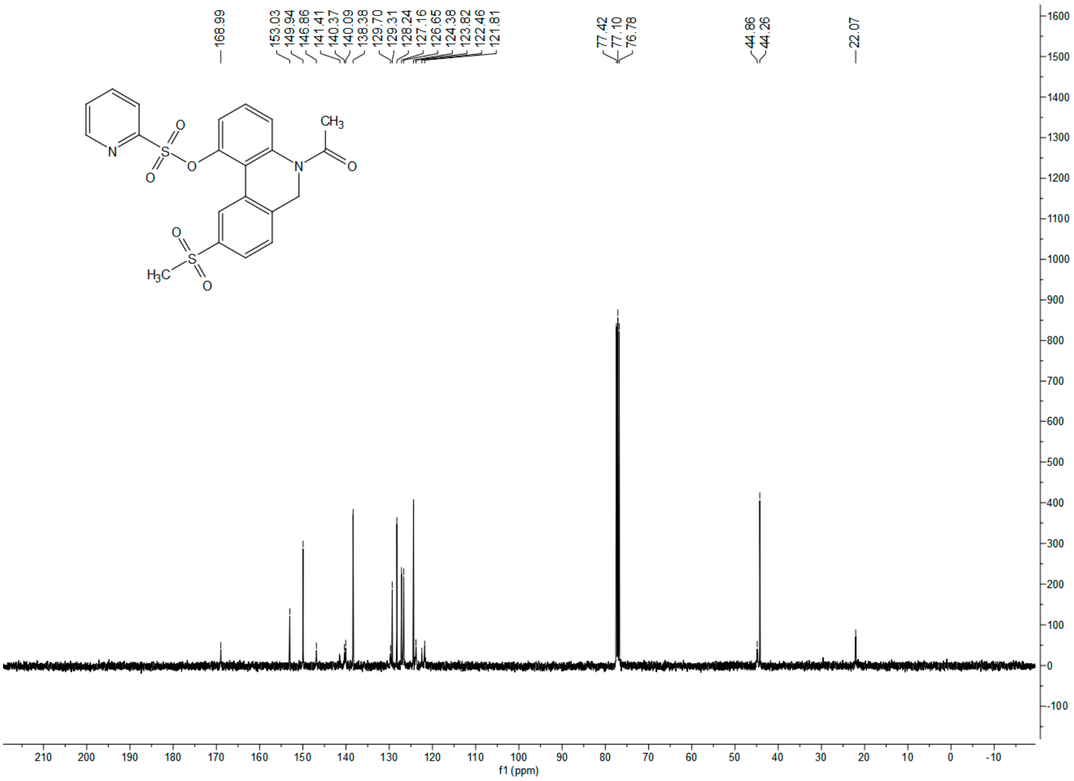

^{1}H NMR spectra of compound **2o**

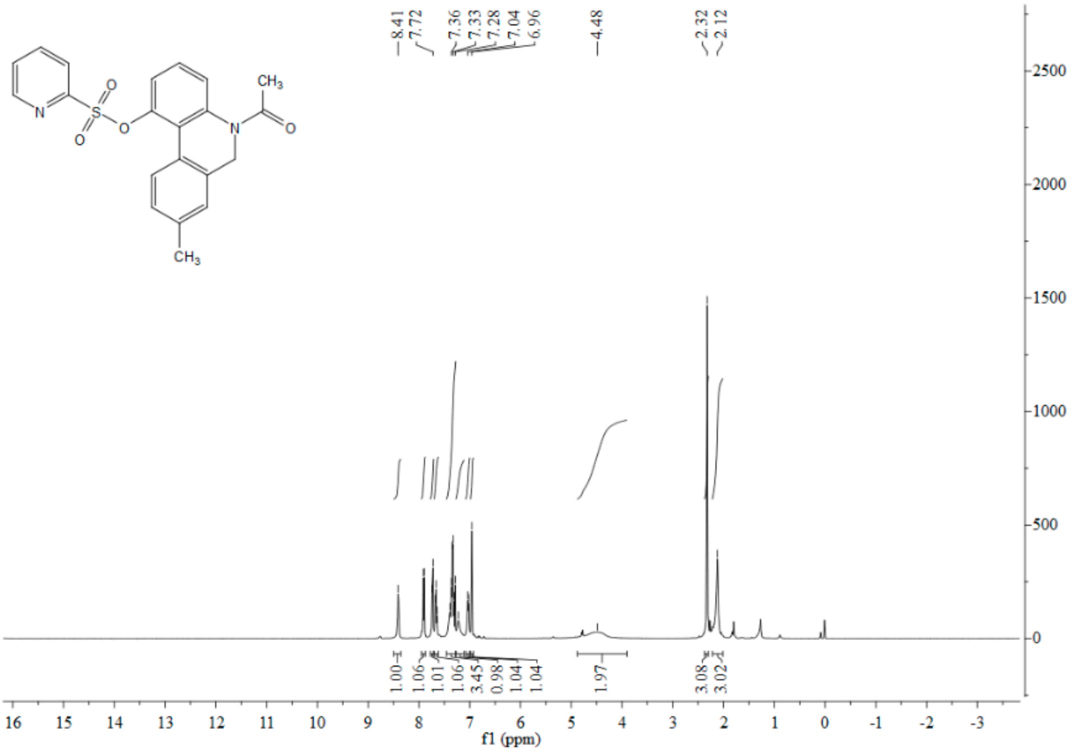

^{13}C NMR spectra of compound **2o**

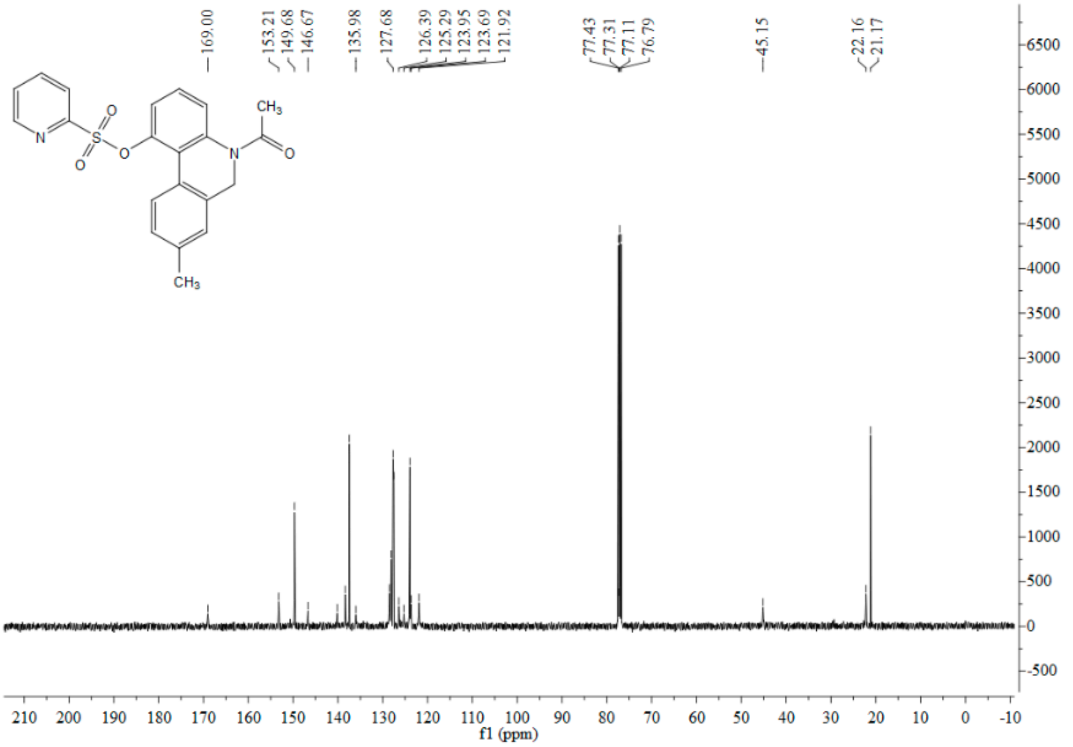

^{1}H NMR spectra of compound **2p**

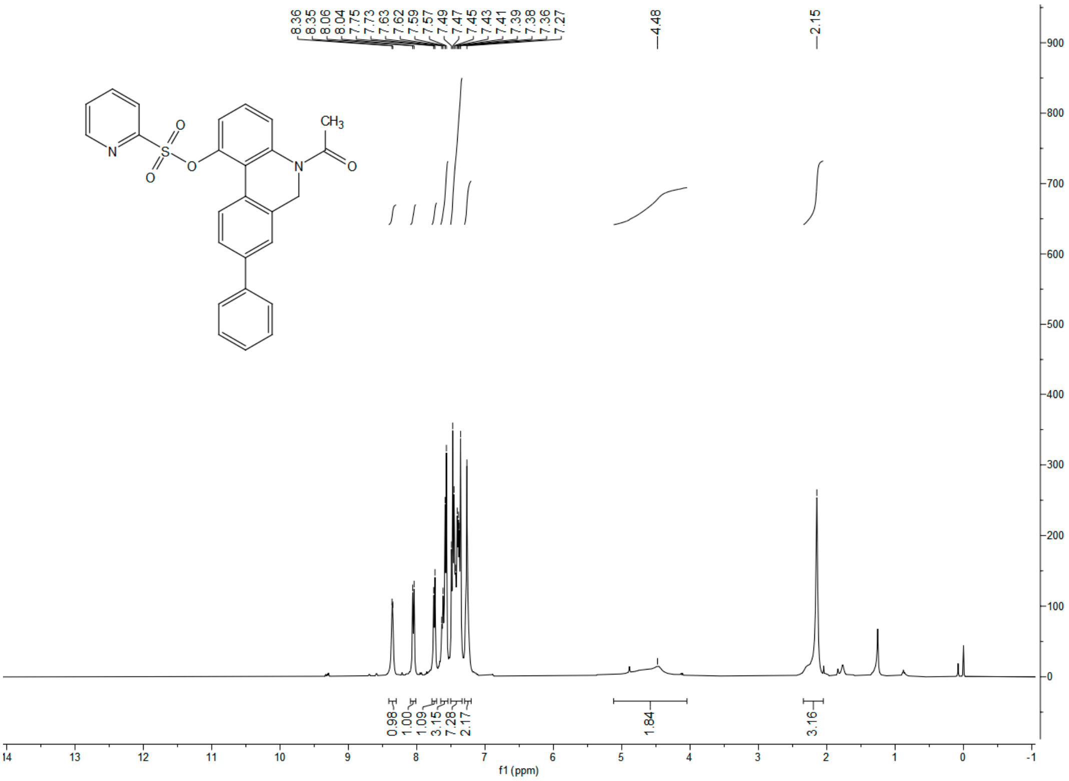

^{13}C NMR spectra of compound **2p**

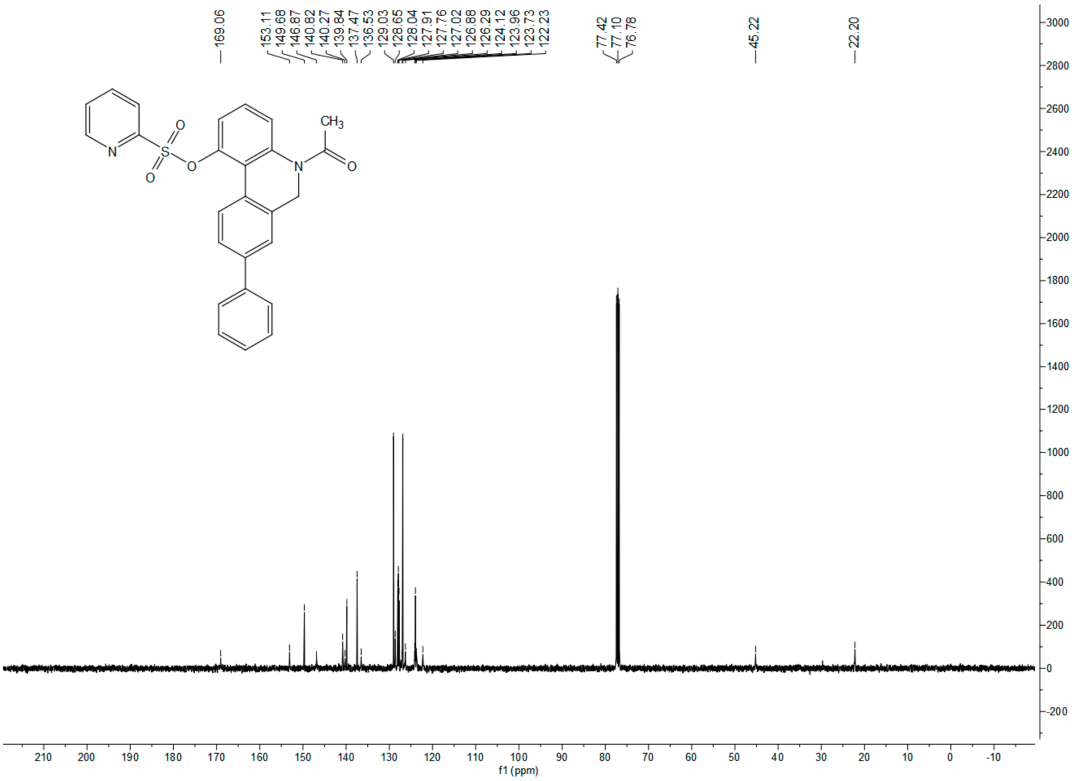

^{1}H NMR spectra of compound **2q**

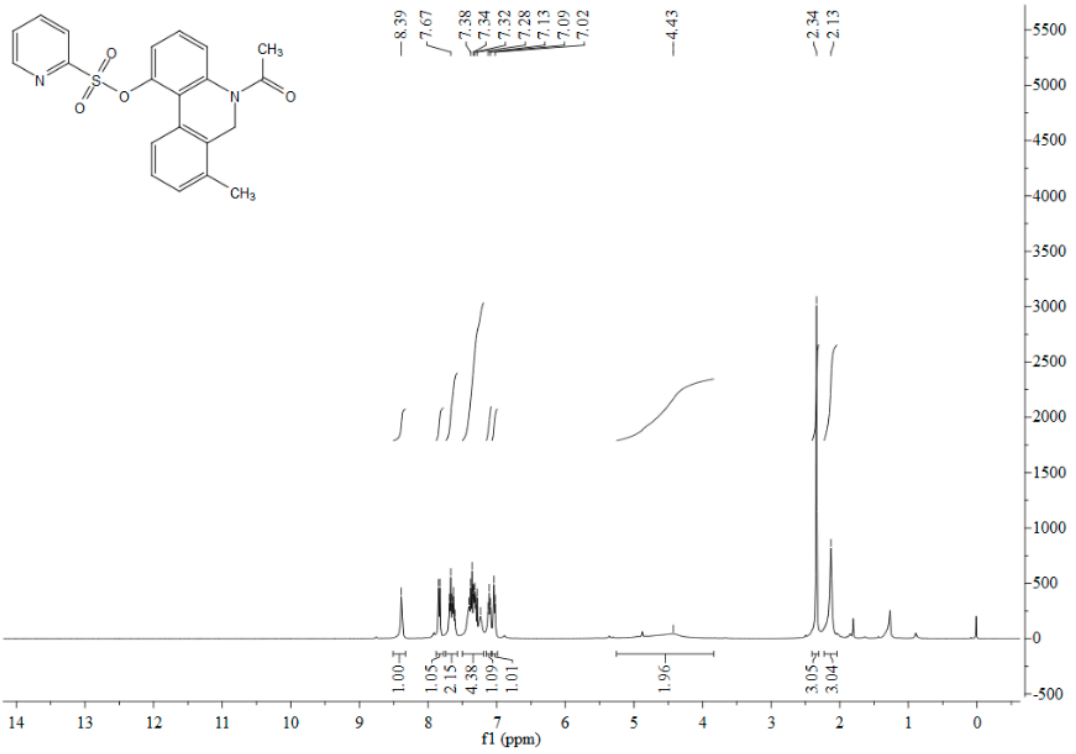

^{13}C NMR spectra of compound **2q**

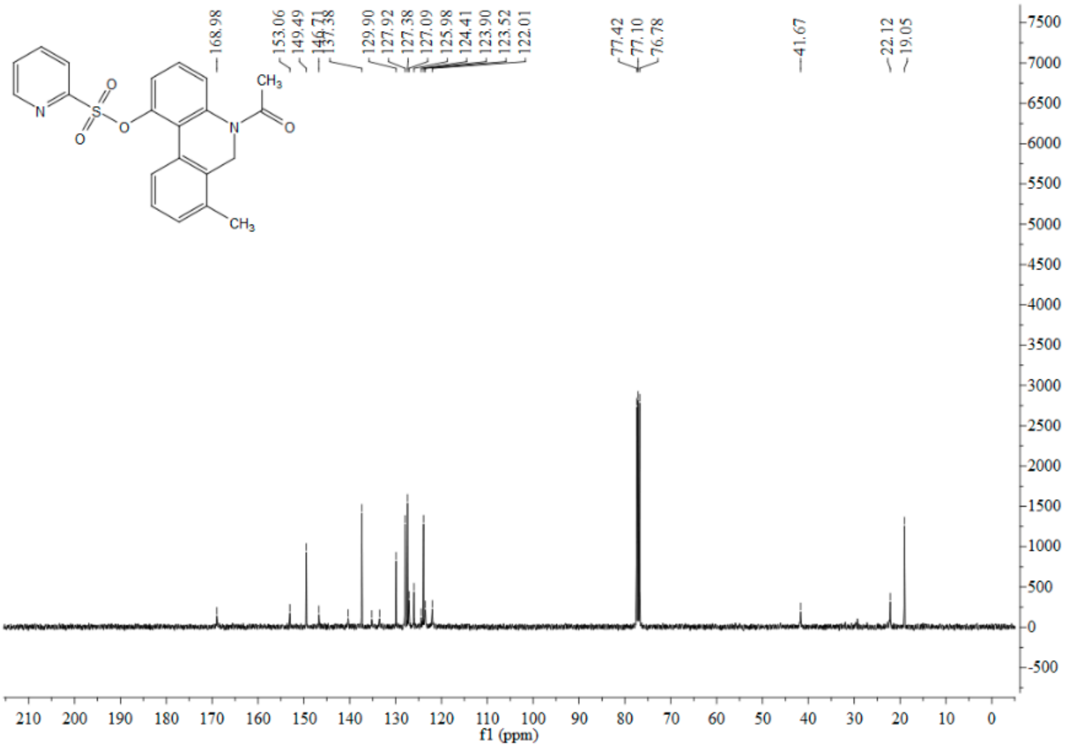

^{1}H NMR spectra of compound **2r**

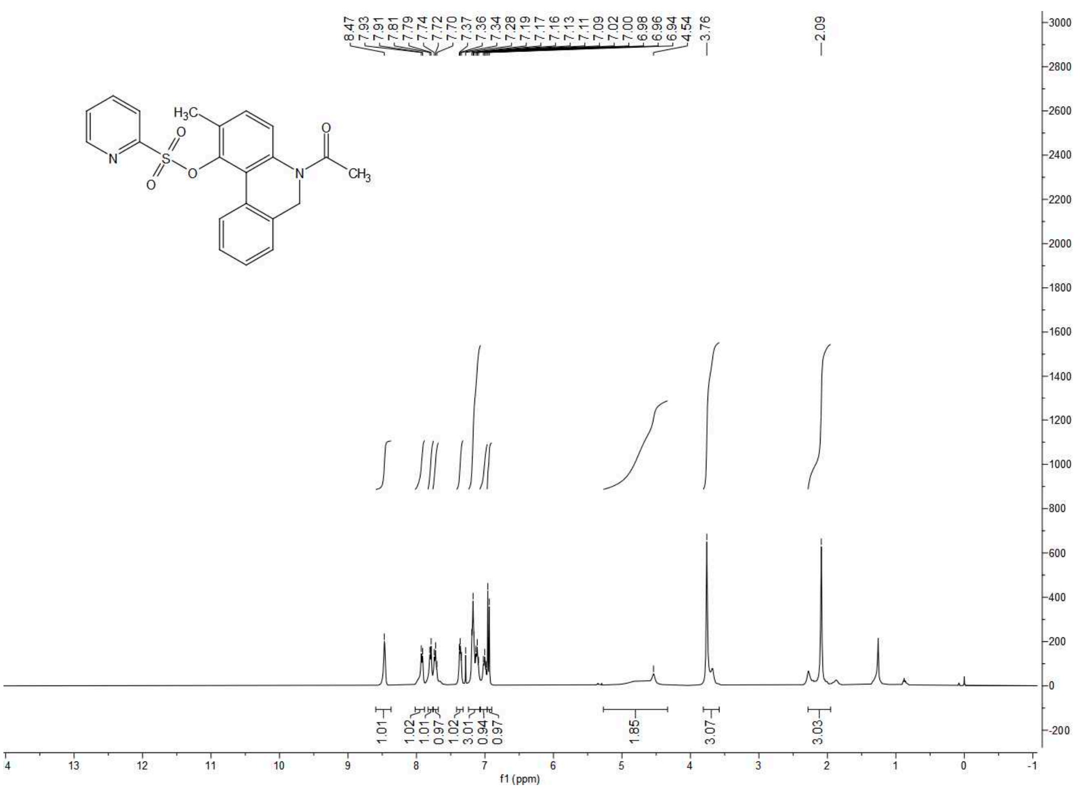

^{13}C NMR spectra of compound **2r**

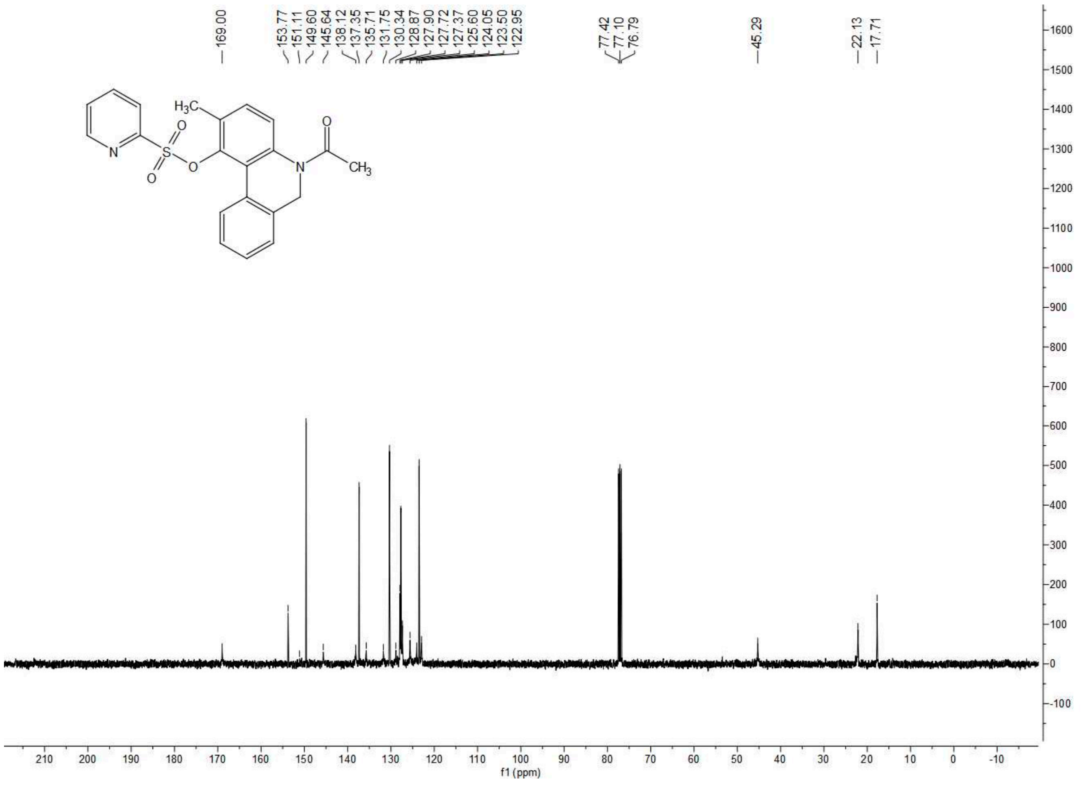

^{1}H NMR spectra of compound **2s**

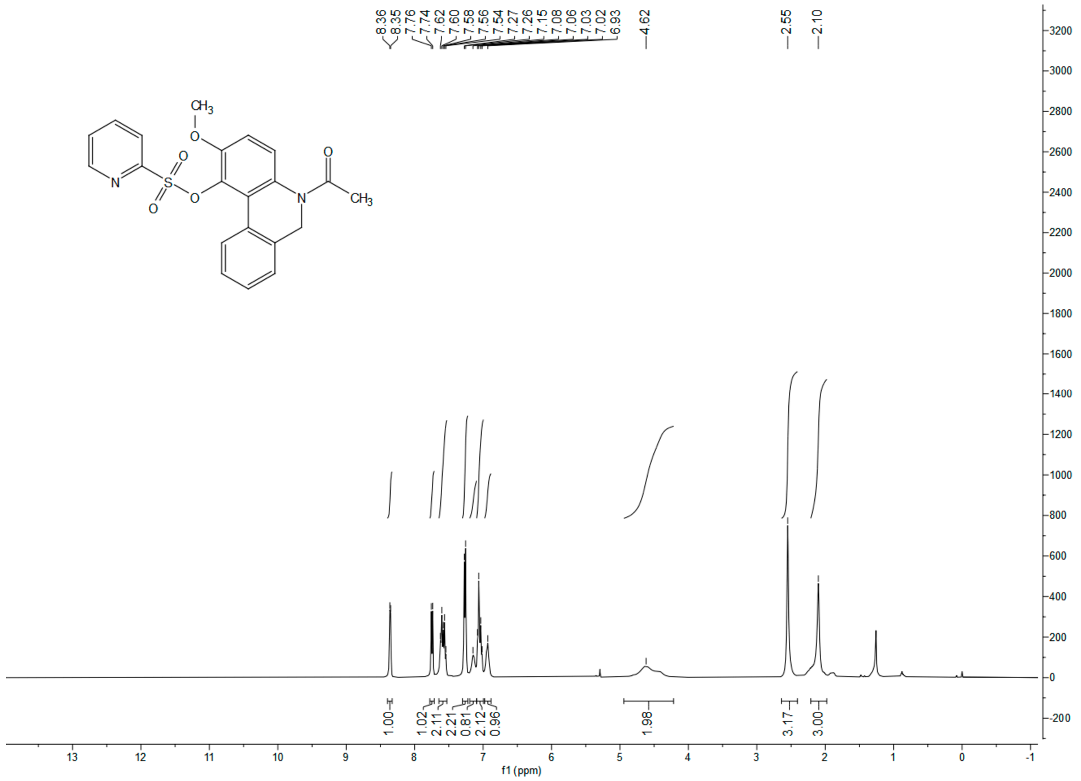

^{13}C NMR spectra of compound **2s**

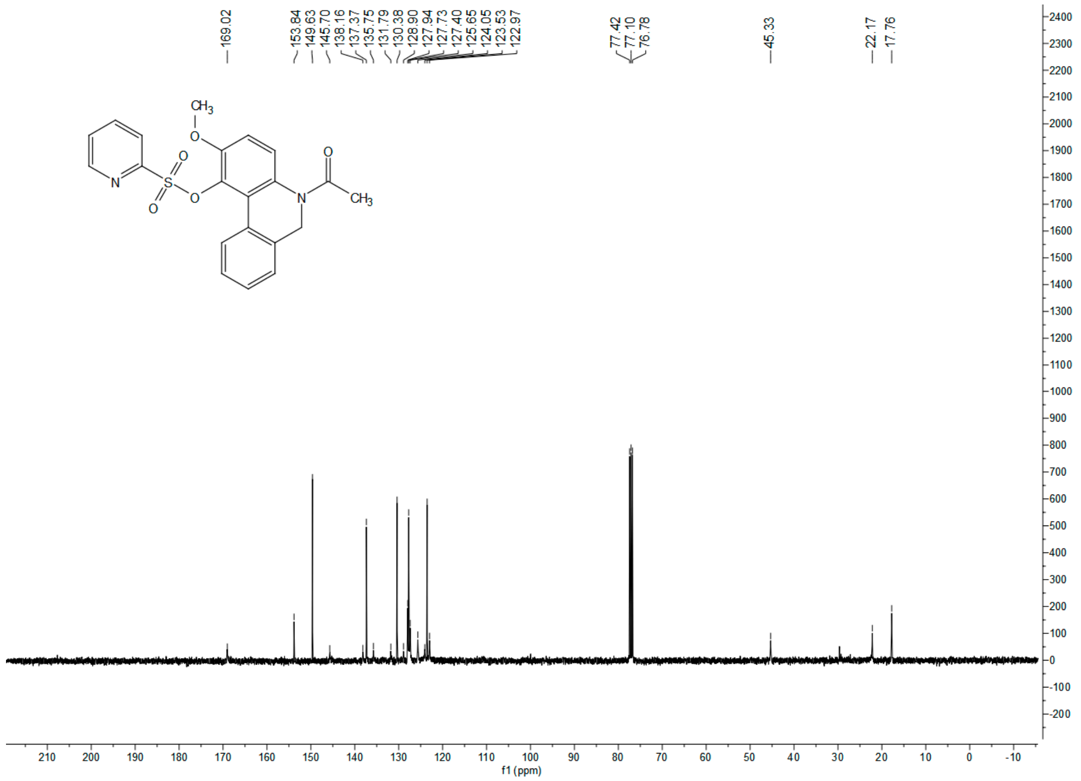

^{1}H NMR spectra of compound **2t**

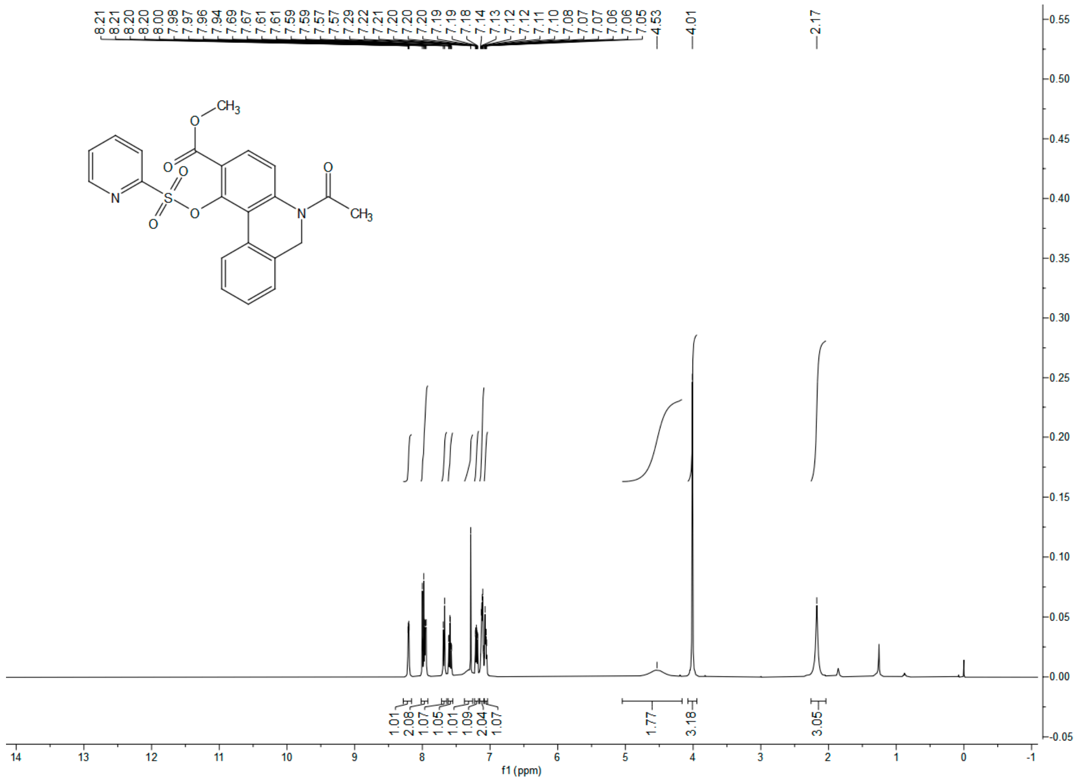

^{13}C NMR spectra of compound **2t**

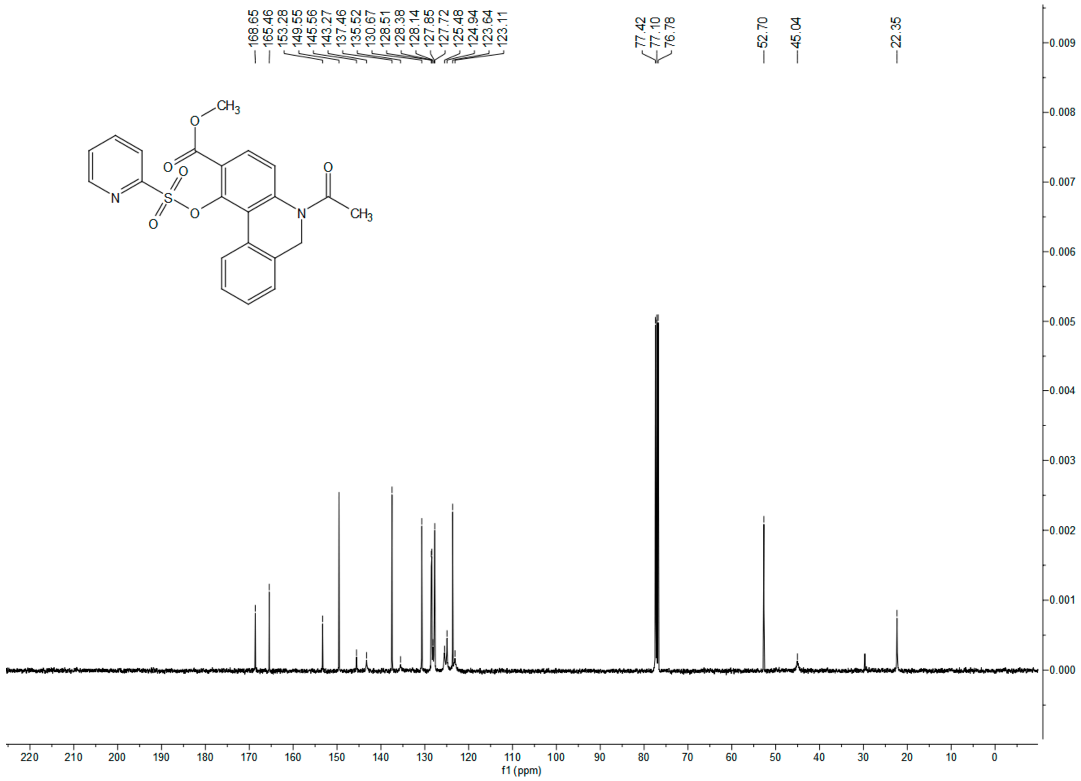

6. Spectral copies of **3-7** ^{1}H NMR and ^{13}C NMR

^{1}H NMR spectra of compound **3**

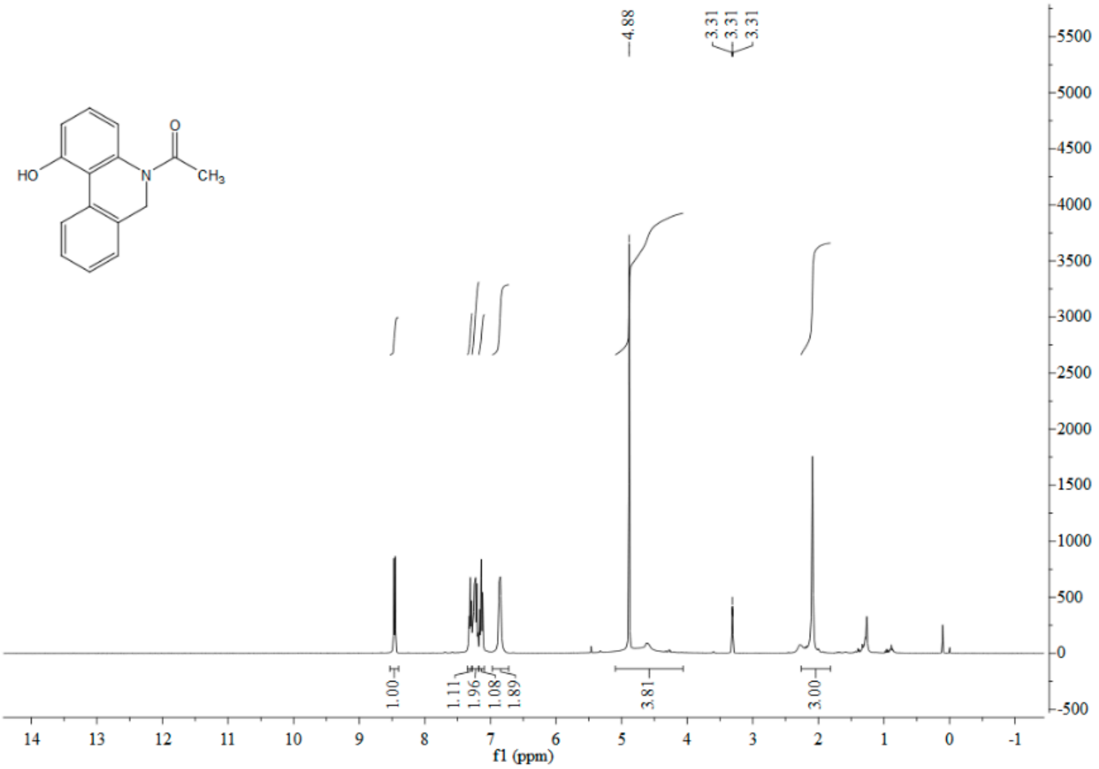

^{13}C NMR spectra of compound **3**

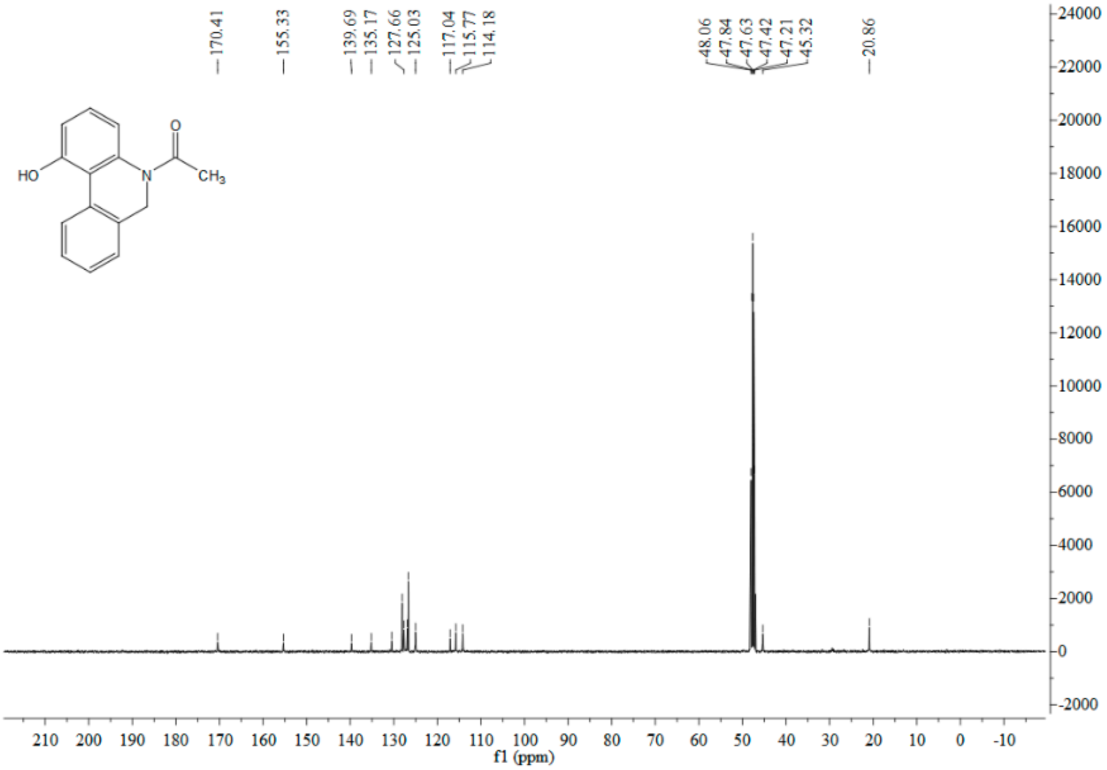

^{1}H NMR spectra of compound **4**

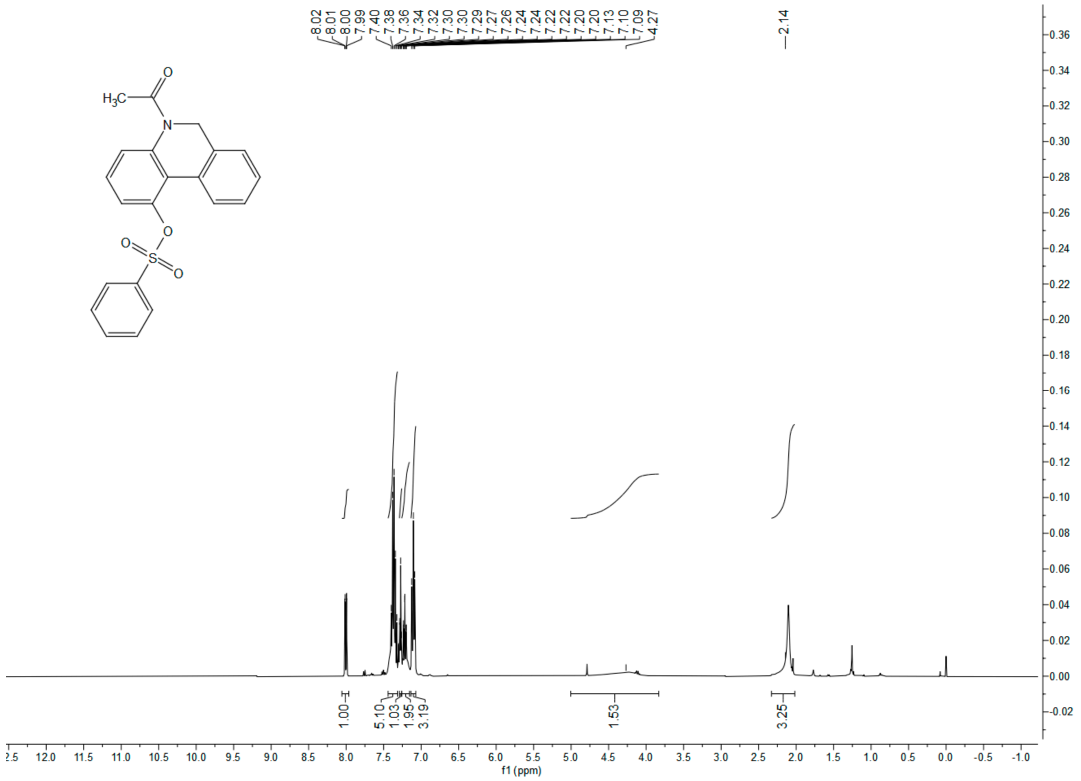

^{13}C NMR spectra of compound **4**

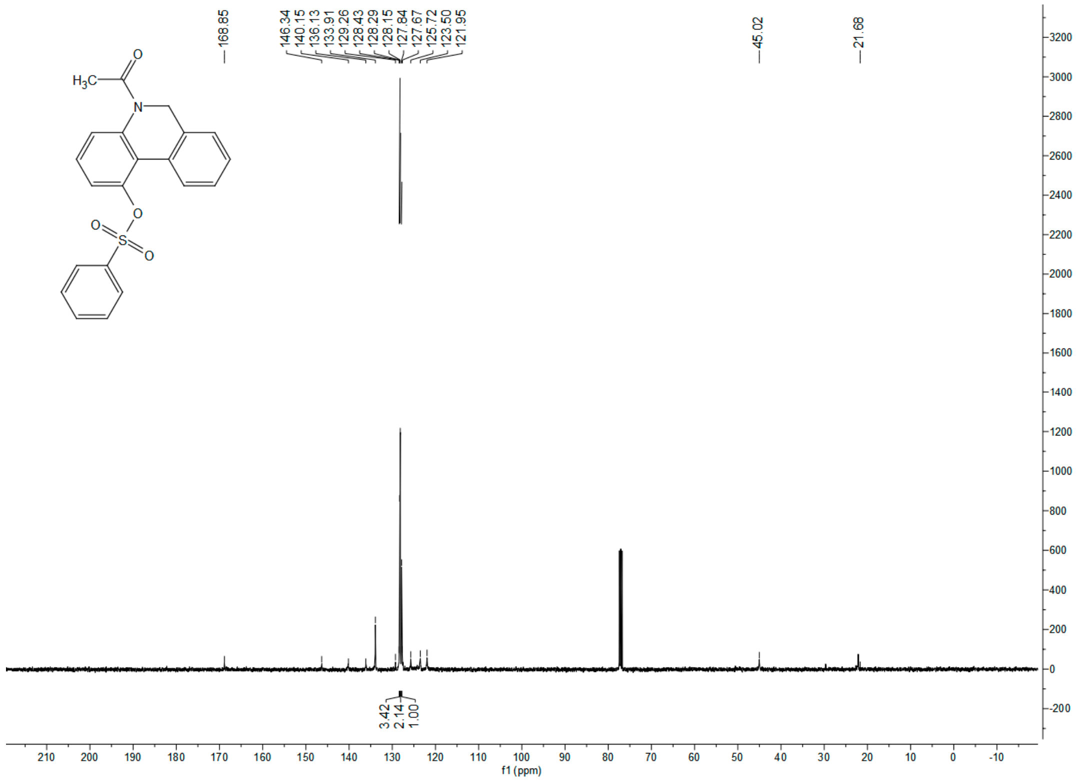

^{1}H NMR spectra of compound **5**

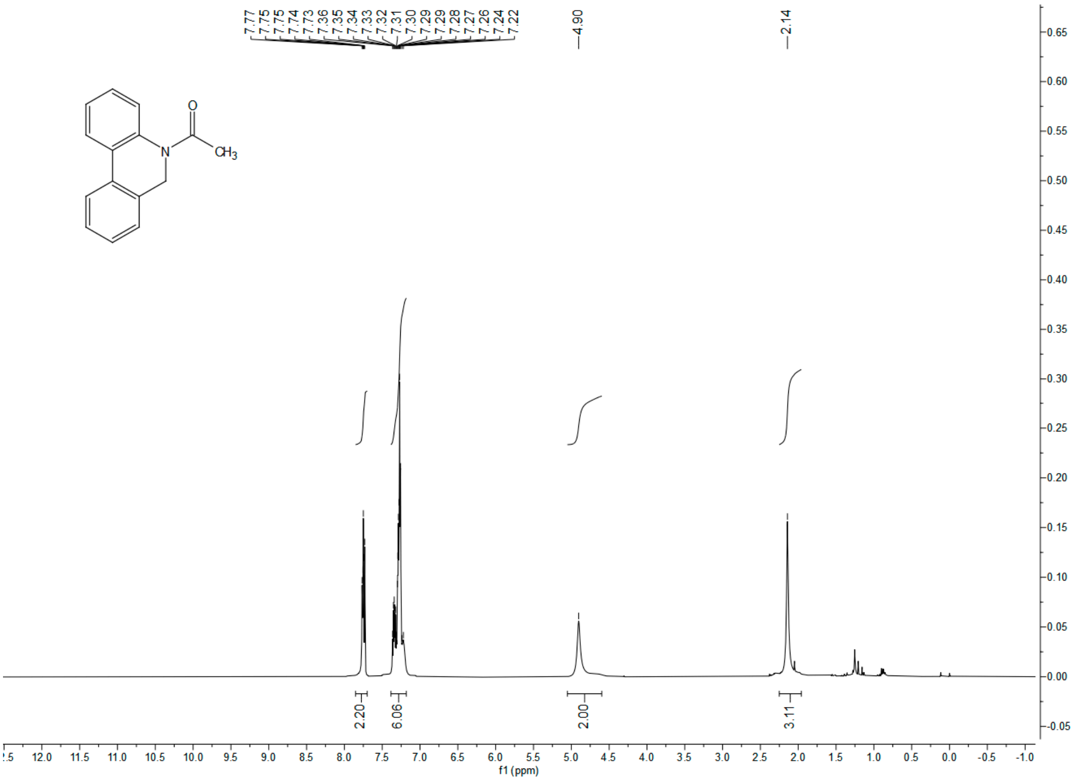

^{13}C NMR spectra of compound **5**

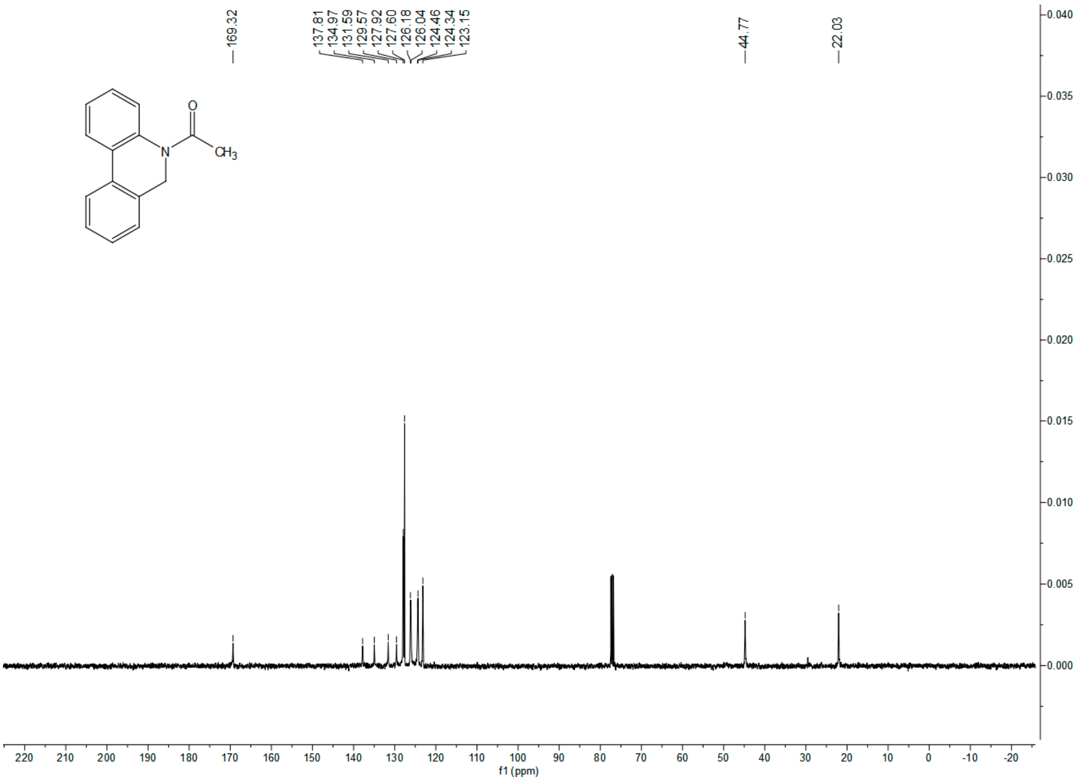

^{1}H NMR spectra of compound **6**

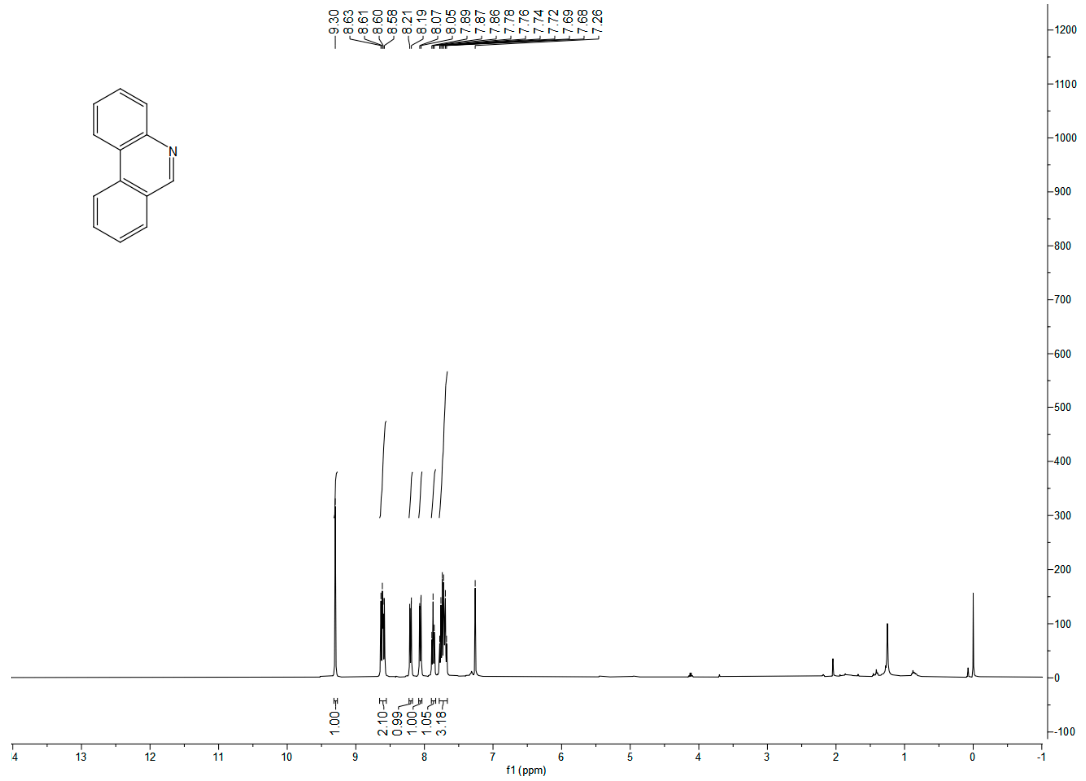

^{13}C NMR spectra of compound **6**

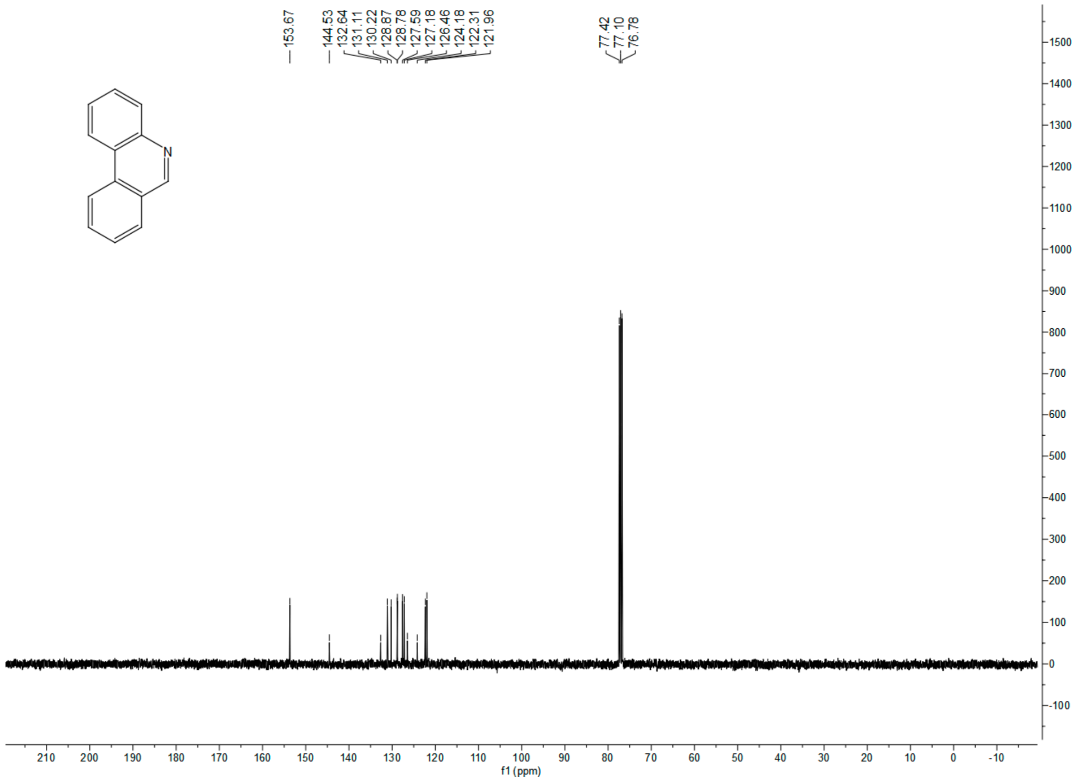

^{1}H NMR spectra of compound **7**

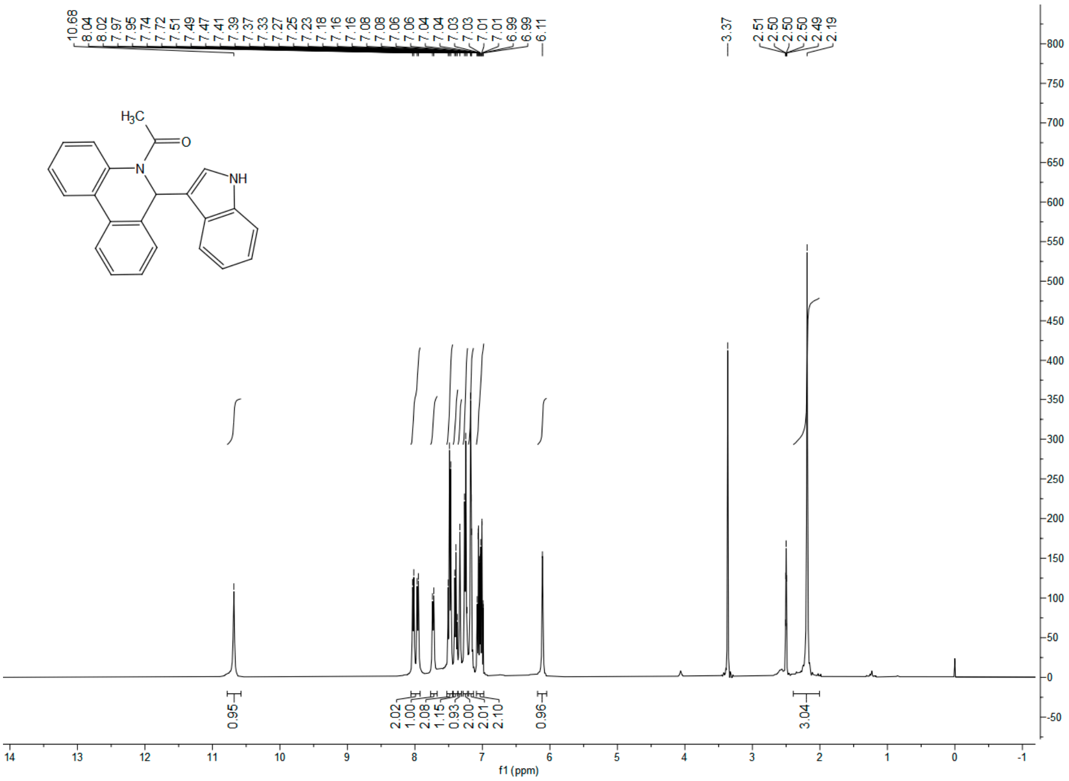

^{13}C NMR spectra of compound **7**

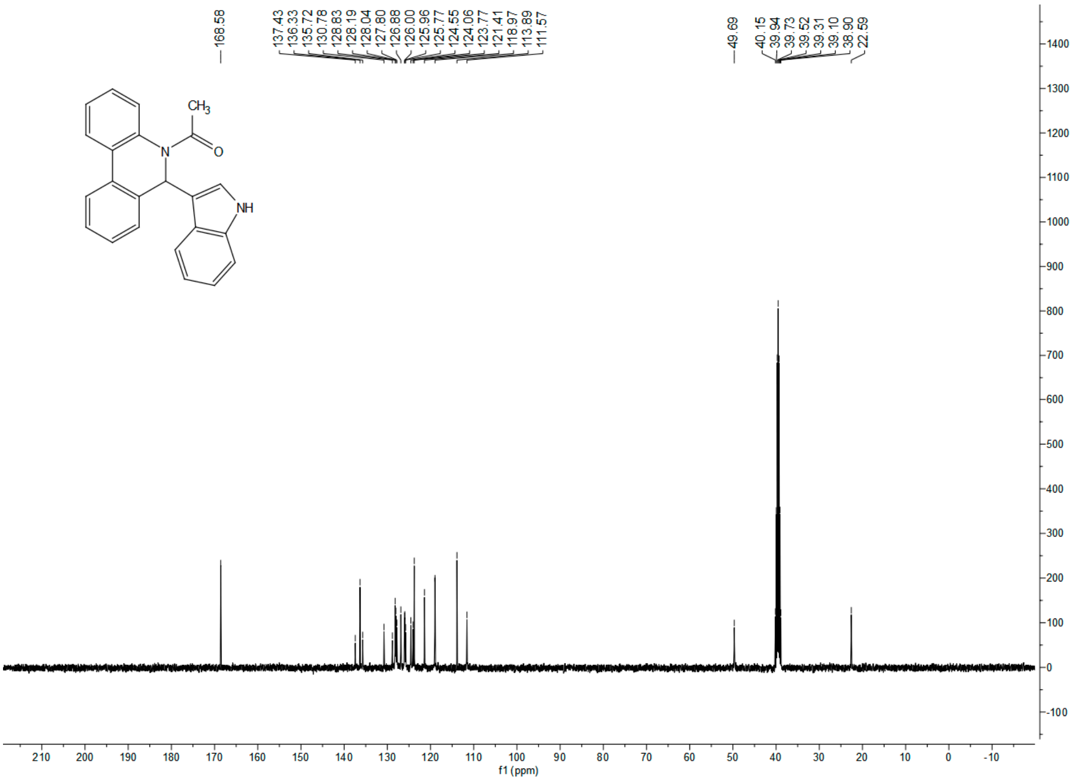

7. X-ray Crystal Structure of Compounds **2a** (CCDC: 1985688)

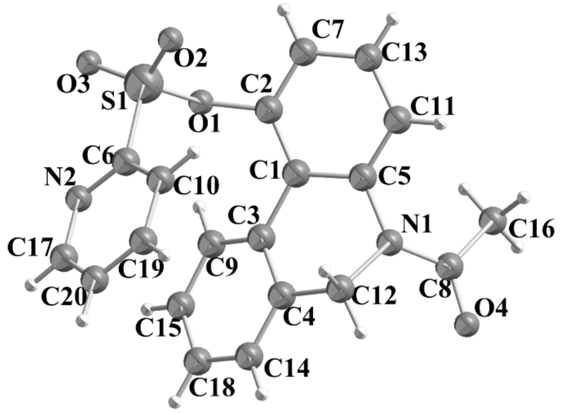

Figure S2. Crystallographic structure data parameters of 2a

Identification code	**2a**
Empirical formula	C20 H16 N2 O4 S
Formula weight	380.41
Temperature	296.15 K
Wavelength	0.71073 A
Crystal system, space group	Monoclinic, P 1 21/c 1
Unit cell dimensions	a = 7.5334(15) A alpha = 90 deg.
	b = 20.396(4) A beta = 105.500(4) deg
	c = 12.054(2) A gamma = 90 deg.
Volume	1784.8(6) A^3
Z, Calculated density	4, 1.416 Mg/m^3
Absorption coefficient	0.211 mm^-1
F(000)	792
Crystal size	0.52 x 0.43 x 0.40 mm
Theta range for data collection	1.997 to 25.497 deg.
Limiting indices	-9<=h<=8, -22<=k<=24, -11<=l<=14
Reflections collected / unique	9383 / 3308 [R(int) = 0.1193]
Completeness to theta = 25.242	99.7 %
Absorption correction	None
Refinement method	Full-matrix least-squares on F^2

Data / restraints / parameters	3308 / 0 / 245
Goodness-of-fit on F^2	0.918
Final R indices [I>2sigma(I)]	R1 = 0.0558, wR2 = 0.1340
R indices (all data)	R1 = 0.0915, wR2 = 0.1526
Largest diff. peak and hole	0.789 and -0.372 e.A^-3
